# From sequence to enzyme mechanism using multi-label machine learning

**DOI:** 10.1186/1471-2105-15-150

**Published:** 2014-05-19

**Authors:** Luna De Ferrari, John BO Mitchell

**Affiliations:** 1 Biomedical Sciences Research Complex and EaStCHEM School of Chemistry, Purdie Building, University of St Andrews, North Haugh, St Andrews, Scotland KY16 9ST, UK

## Abstract

**Background:**

In this work we predict enzyme function at the level of chemical mechanism, providing a finer granularity of annotation than traditional Enzyme Commission (EC) classes. Hence we can predict not only whether a putative enzyme in a newly sequenced organism has the potential to perform a certain reaction, but how the reaction is performed, using which cofactors and with susceptibility to which drugs or inhibitors, details with important consequences for drug and enzyme design. Work that predicts enzyme catalytic activity based on 3D protein structure features limits the prediction of mechanism to proteins already having either a solved structure or a close relative suitable for homology modelling.

**Results:**

In this study, we evaluate whether sequence identity, InterPro or Catalytic Site Atlas sequence signatures provide enough information for bulk prediction of enzyme mechanism. By splitting MACiE (Mechanism, Annotation and Classification in Enzymes database) mechanism labels to a finer granularity, which includes the role of the protein chain in the overall enzyme complex, the method can predict at 96% accuracy (and 96% micro-averaged precision, 99.9% macro-averaged recall) the MACiE mechanism definitions of 248 proteins available in the MACiE, EzCatDb (Database of Enzyme Catalytic Mechanisms) and SFLD (Structure Function Linkage Database) databases using an off-the-shelf K-Nearest Neighbours multi-label algorithm.

**Conclusion:**

We find that InterPro signatures are critical for accurate prediction of enzyme mechanism. We also find that incorporating Catalytic Site Atlas attributes does not seem to provide additional accuracy. The software code (ml2db), data and results are available online at http://sourceforge.net/projects/ml2db/ and as supplementary files.

## Background

Previous research has already been very successful in predicting enzymatic function at the level of the chemical reaction performed, for example in the form of Enzyme Commission numbers (EC) or Gene Ontology terms. A much less researched problem is to predict *by which mechanism* an enzyme carries out a reaction. Differentiating enzymatic mechanism has important applications not only for biology and medicine, but also for pharmaceutical and industrial processes which include enzymatic catalysis. For example, biological and pharmaceutical research could leverage different mechanisms in host and pathogen for drug design, or to evaluate if antibiotic resistance is likely to appear in certain micro-organisms. And enzymes that perform the same reaction but require less costly cofactors can be more interesting candidates for industrial processes. Predicting the existence of a mechanism of interest in a newly sequenced extremophile, for example, could lead to applications in medicine or industry and to significant cost savings over non-biological industrial synthesis.

An enzyme is any protein able to catalyse a chemical reaction. In this work we do not focus on the questions associated with defining or assigning enzyme mechanisms, but rather take our definitions and assignments directly from the MACiE (Mechanism, Annotation and Classification in Enzymes) database [1-3]. Version 3.0 of the MACiE database contains detailed information about 335 different enzymatic mechanisms. Thanks to this information manually derived from literature, it is possible in MACiE to compare exemplars of enzymes that accept the same substrate and produce the same product, but do so using a different chemical mechanism, intermediate activation step or cofactor. Unfortunately, relatively few proteins are annotated with MACiE identifiers because confirming the exact mechanism of an enzyme requires significant effort by experimentalists and study of the literature by annotators.

Given the limited available examples, the aim of this work is to verify whether prediction of enzyme mechanism using machine learning is possible, and to evaluate which attributes best discriminate between mechanisms. The input is exclusively a protein sequence. The output, or predicted class labels, comprises zero or more MACiE mechanism identifiers, while the attributes used are sequence identity, InterPro [4] sequence signatures and Catalytic Site Atlas (CSA) site matches [5].

InterPro sequence signatures are computational representations of evolutionarily conserved sequence patterns. They vary from short, substitution-strict sets of amino acids representing binding sites to longer and substitution-relaxed models of entire functional domains or protein families. The Catalytic Site Atlas sites are akin to InterPro patterns, but they do not provide an evolutionary trace, more a record of an individual catalytic machinery, derived from a single Protein Data Bank [6] 3D structure which is transformed into a strict sequence pattern containing only the catalytic amino acids.

Only three proteins in our data have more than one mechanism label, because the current dataset privileges simple, one catalytic site enzymes. However, here we use a multi-label (and not only multi-class) machine learning scheme to be able to predict real life enzymes with multiple active sites or alternative mechanisms. Multi-label learning also provides flexibility by allowing seamless integration of additional labelling schemes. For example, Enzyme Commission numbers or Gene Ontology terms could be predicted together with mechanism. We evaluate the method by training a classifier on enzymes with known mechanisms. The classifier learns from the available attributes (for example sequence signatures) and then attempts to predict the mechanisms of a previously unseen test sequence. The quality of the predictions on the test set is evaluated using a number of metrics such as accuracy, precision, recall and specificity.

### Previous work

To our knowledge, no previous research has attempted bulk prediction of enzymatic *mechanism* from sequence. However, past research has proved that the Enzyme Commission class of enzymes can be successfully predicted even for distantly related sequences using exclusively InterPro signatures [7-9]. Traube *et al.*[10] used QSAR and enzyme mechanism to predict and design covalent inhibitors for serine and cysteine proteases. Their method, like ours, does not require a solved protein structure, but its mechanism predictions are aimed at drug design and not easily portable to enzymes other than proteases. Choi *et al.*[11] use sequence to predict the existence and position of probable catalytic sites (grouped and aligned by Enzyme Commission number) with about 25% accuracy (approximately 8% better than random) but their prediction does not specify which *mechanism* the enzyme might be using in that active site. Other work tried to predict whether an amino acid is catalytic, and could in principle lead towards mechanism identification, but in practice has not been used to infer mechanism, only enzyme reaction. Using 3D structural information, Chea *et al.*[12] used graph theory to predict whether an amino acid is catalytic, followed by filtering using solvent accessibility and compatibility of residue identity since some amino acids are less likely to be involved in active catalysis. But their output is a binary label (catalytic or not) and not a prediction of mechanism. Using only sequence, Mistry *et al.*[13] have developed a strict set of rules to transfer experimentally determined active site residues to other Pfam family proteins, achieving a 3% FP rate, 82% specificity and 62% sensitivity. However, again, they do not link the active site residues to the mechanism performed.

## Methods

### Database sources and datasets

Data were taken from MACiE (Mechanism, Annotation and Classification in Enzymes database) [3] version 3.0, EzCatDb (Enzyme Catalytic-mechanism Database) [14], SFLD (Structure Function Linkage Database) [15], UniProtKB [16], InterPro [4] and Expasy Enzyme [17] in September 2013.

The complete data set includes 540 proteins that have been manually annotated with a MACiE mechanism in either MACiE, EzCatDb or SFLD, corresponding to 335 different MACiE mechanisms and 321 Enzyme Commission numbers. Three of these enzymes, the beta lactamases having UniProt entry name BLAB_SERMA from *Serratia marcescens* (beta-lactamase IMP-1, UniProt accession P5269), BLA1_STEMA from *Stenotrophomonas maltophilia* (metallo-beta-lactamase L1, P52700) and BLAB_BACFG from *Bacteroides fragilis* (beta-lactamase type II, P25910) have two MACiE mechanism labels in our dataset, due to the fact that EzCatDb does not distinguish between MACiE mechanisms M0015 and M0258. Both mechanisms are class B beta lactamase reactions, but performed with different catalytic machinery: M0015 uses an Asn residue, while M0258 uses Asp and Tyr. So the need for multi-label prediction is not strong for our dataset, however, multi-label classification is essential for mechanism prediction of real life multi-domain proteins. UniProt Swiss-Prot already contains 12,456 enzymes with more than one Enzyme Commission number. As just one example, the replicase polyprotein 1ab of the bat coronavirus (UniProt name R1AB_BC279 or accession number P0C6V) is cleaved into fifteen different chains, several of which are enzymes with one or more EC numbers, thus totalling nine Enzyme Commission numbers for a single transcript, varying from cysteine endopeptidase to RNA-directed RNA polymerase activities.

### Class labels

An instance in our datasets is composed of a protein identifier (a UniProt accession number), a set of attributes (for example, the absence or presence of a sequence feature or the sequence identity with other sequences), and zero or more class labels representing the MACiE mechanisms of the enzyme, where available. Several MACiE mechanism entries can exist for one Enzyme Commission number. A MACiE mechanism identifier corresponds to a detailed mechanism entry modelled on one PDB [18] 3D structure and its associated literature. The entry describes not only the enzyme reaction, but also the catalytic machinery (reactive amino acids, organic and metal cofactors) used to perform the catalysis, down to the role of the individual amino acids, cofactor and molecular intermediates in each reaction step (such as proton or electron donor or acceptor and others) and the chemical mechanism steps (such as bond breaking, bond formation, electron transfer, proton transfer, tautomerisation and others) in temporal order.

A detailed analysis of the false positives generated by an initial prediction test highlighted the presence of distinct and diverse enzyme moieties labelled with the same MACiE mechanism code. For example, MACiE code M0013 (amine dehydrogenase) is used in MACiE only to annotate the methylamine dehydrogenase *light* chain of *Paracoccus denitrificans* (DHML_PARDE, P22619). However, in the database EzCatDb, the Paracoccus denitrificans *heavy* chain (DHMH_PARDE, P29894) is also annotated with MACiE code M0013, possibly because the holoenzyme is a tetramer of two light and two heavy chains (with the light chain hosting the active site). There is little or no similarity between each light and heavy chain (sequence identity < 12%), while the light chains are highly conserved within related organisms (sequence identity > 90%).

We thus proceeded to examine our training set to decide when the original MACiE mechanism code could be enriched with two or more sub-labels providing a better description of the underlying organisation of the enzyme chains. For all MACiE labels we did the following: 1. if the label annotates two or more proteins, we examined the "subunit structure" section of each UniProt protein, 2. if the section contained words such as heterodimer, heterotetramer or complex, we proceeded to split the MACiE label into two or more labels according to the enzyme complex subunits, and 3. we then re-annotated each protein with one of the new and more appropriate MACiE + subunit labels. We would like to stress that during this process the original MACiE mechanism annotations remain *unchanged*. The additional subunit information improves the learning, but, if the user so wishes, can easily be ignored simply by discarding any text beyond the 5th character (thus transforming, for example, M0314_component_I into M0314).

To give an example of the procedure to generate the new labels, MACiE label M0314 (anthranilate synthase) annotates two proteins in MACiE: TRPE_SULSO from the bacterium *Sulfolobus solfataricus* (anthranilate synthase component I, Q06128) and TRPG_SULSO (anthranilate synthase component II, Q06129) also from *Sulfolobus solfataricus*. In addition, the database EzCatDb uses the same MACiE label to annotate the corresponding component I and II of another bacterium, *Serratia marcescens* (EzCatDb identifier D00526, UniProt accessions TRPE_SERMA, P00897 and TRPG_SERMA, P00900). The "subunit structure" section of these four proteins in UniProt specifies: "Subunit structure: tetramer of two components I and two components II". We thus proceed to re-annotate the four proteins as M0314_component_I (*Sulfolobus* Q06128 and *Serratia* P00897, both described as anthranilate synthase component I) and M0314_component_II (*Sulfolobus* Q06129 and *Serratia* P00900, both described as anthranilate synthase component II).

The set of the old MACiE labels which did not require splitting and the new split labels (such as M0314_component_I, M0314_component_II, M0013_light_chain, M0013_heavy_chain etc.) is referred to as MACiE + subunit labels or simply mechanism labels.

As previously noted, in our current data most mechanisms only have one annotated protein exemplar and hence cannot be used for cross-validation or leave-one-out validation: the protein would always be either exclusively in the training set or exclusively in the testing set. This leaves us with only 82 MACiE + subunit mechanisms (corresponding to 73 classic MACiE mechanisms) having at least two protein examples, thus providing 248 enzyme sequences usable for cross-validation. This dataset is from now on referred to as the *mechanism* dataset.

However, the proteins belonging to mechanisms having only one exemplar can still be pooled together and used as negative examples for the other mechanisms (*negative* dataset), and the resulting false positive predictions can be analysed to assess why the method makes certain mistakes.

Also, in nearest neighbours algorithms, an instance must necessarily have a closest neighbour. An instance having no attributes in common with any other instance will "gravitate" towards the shortest available instance in the set (the instance with the fewest attributes). In order to avoid these artefacts, two empty instances (instances with no attributes and no class labels) have been added to the *mechanism* dataset for the training-testing experiments.

The set of UniProt Swiss-Prot proteins lacking Enzyme Commission annotation has also been used (*swissprot-non-EC*) as a "negative" test set. This set contains 226,213 proteins (as of September 2013) which are most probably non-enzymes (or have a yet unknown catalytic activity or an enzymatic activity which was mistakenly overlooked by curators). Of these, only 68,677 share at least one InterPro signature with a protein in the *mechanism* or *negative* datasets and could hence be mispredicted as enzymes (all the other proteins in the *swissprot-non-EC* set are, by definition, automatically and correctly predicted as not having a mechanism when using the *InterPro* attributes).

### Attributes

Once defined the mechanism class labels to be predicted, we analysed which sequence-based attributes or features could be used for learning. More specifically, we have compared the accuracy of enzyme mechanism predictions when various different sets of attributes are used. The *InterPro* set of attributes includes the presence (1) or absence (0) of each InterPro signature for each sequence in the given protein dataset. InterPro is an extensive database of conserved sequence signatures and domains [4] that can be computed from sequence data alone and for any sequence using the publicly available InterProScan algorithm [4,19]. The 248 proteins in the *mechanism* dataset, for example, have 444 distinct InterPro attribute values, with an average of 4.4 InterPro signatures per protein.

InterPro signatures are composed of one or several sub-signatures provided by its repositories: GENE3D [20], HAMAP [21], PANTHER [22], Pfam [23], PIRSF [24], PRINTS [25], ProDom [26], PROSITE patterns and profiles [27], SMART [28], SUPERFAMILY [29] and TIGRFAM [30]. One or more of these sub-signatures usually correspond to one InterPro signature. However, some of these sub-signatures have not been integrated into InterPro because they provide too many false positives, do not have enough coverage or do not pass other criteria fixed by InterPro. We have tried using all these sub-signatures (integrated or not) as attributes for learning, to understand if they could provide a more powerful and finely grained alternative to the classic InterPro signatures.

Another set of attributes represents the presence (or absence) of a sequence match versus one of the Catalytic Site Atlas active sites (CSA 2D or simply *CSA* attributes). Each CSA 2D site is a tuple of active amino acids that must match the given sequence both for position and amino acid type.

In order to compare learning by sequence with learning based on structure, we matched our dataset also against the Catalytic Site Atlas three dimensional templates [31] (CSA 3D). CSA templates store the geometrical position of exclusively the atoms of the residues involved in a catalytic site. A residue is considered catalytic if it is chemically involved in the catalysis, if it alters the pKa of another residue or water molecule, if it stabilises a transition state or if it activates a substrate, but not if it is involved solely in ligand binding. Each CSA template is matched against the protein structure using the JESS algorithm [32].

To generate CSA 3D templates matches we first selected an exemplar (best) PDB X-ray structure for each UniProt protein in the *mechanism* dataset. To select the exemplar structure we collected all PDB structures for each UniProt record and chose the structures that covered the longest stretch of the protein sequence. If several structures of identical coverage and resolution existed, we chose the structure(s) with the best (highest) resolution. If several structures still existed, we chose the last when ordered alphabetically by PDB structure identifier. We then used the ProFunc service [33] to scan each exemplar PDB against CSA 3D templates (*CSA 3D* data set). For evaluation we also compare "best" matches against the MACiE dataset (having an E value below 0.1) versus all matches provided by ProFunc (E value below 10.0).

The various sets of attributes above have been evaluated, alone or in combination, for their ability to predict enzyme mechanism in the datasets presented. Combining attribute sets such as *InterPro* and *CSA* (as in the *InterPro+CSA* attribute set) means that the dataset matrix will have, for each protein row, all CSA columns and all InterPro columns filled with either 1 (signature match) or 0 (no match). This provides a sparse data matrix particularly suitable for large datasets of millions of protein sequences.

Considering though that our current dataset is not large, we have also created two more computationally intensive attribute sets. The first set (*minimum Euclidean distance*) involves calculating the Euclidean distance in the *InterPro* space between the protein of interest and all other proteins (sets of *InterPro* attributes). An attribute vector is then built with as many values as there are mechanisms. As each attribute value (that is, for each mechanism) we keep only the minimum Euclidean distance between the protein of interest and the proteins having that mechanism, giving:

$$a={\left(a_{m}\right)}_{m\in M},a_{m}=\underset{p_{m}\in m}{\text{min}}\text{Euclidean distance}(p,p_{m})$$

where ***a*** is the vector of attribute values composed of one value *a*_*m*_ for each of the *M* mechanisms in the data, *p* is the protein of interest and *p*_*m*_ is a protein having a mechanism *m*. The function *Euclidean distance*(*p*,*p*_*m*_) returns the Euclidean distance between the *InterPro* set of signatures of protein *p* and the *InterPro* set of signatures of another protein *p*_*m*_ having mechanism *m*. We can also note that the k-Nearest Neighbour algorithm must calculate Euclidean distances, but, with the simpler aim of finding the closest instances, it does not usually need to store and manipulate the distances for every protein and mechanism combination.

The second set of attributes (*maximum sequence identity*) is even more computationally intensive because it substitutes distance with sequence identity. It thus requires an alignment between each pair of proteins in the dataset. The sequence identity of each protein versus every other protein in the *mechanism* and *negative* datasets was calculated by downloading the FASTA sequences from UniProt in September 2013 and aligning each pair using the Emboss [34] implementation of the Needleman-Wunsch algorithm [35]. The algorithm was run with the default substitution matrix EBLOSUM62 with gap opening penalty of 10 and gap extension penalty of 0.5. The resulting *maximum sequence identity* vector of attributes is given by:

$$b={\left(b_{m}\right)}_{m\in M},b_{m}=\underset{p_{m}\in m}{\text{max}}\text{sequence identity}(p,p_{m})$$

where ***b*** is the vector of attribute values in the data (composed of one value *b*_*m*_ for each of the *M* mechanisms), *p* is the protein of interest and *p*_*m*_ is a protein having a mechanism *m*. The function *sequence identity*(*p*,*p*_*m*_) returns the sequence identity between the protein sequence *p* and another protein sequence *p*_*m*_ having mechanism *m* (the emitted value can span from zero, if no amino acids could be aligned, to one, if the two sequences are identical).

### Algorithm

Several algorithms [36-51] were evaluated by comparing their precision, recall, accuracy and run time on a leave-one-out prediction of the *mechanism* dataset (see Additional file 1 for full results). The top two algorithms for accuracy (about 96%) and speed (about 24 seconds for 248 instances) are instance-based learning algorithms (Mulan’s [46] BRkNN [45] and Weka’s [50] IBk [36] with a label powerset multi-label wrapper). The Mulan Hierarchical Multi Label Classifier (HMC) [47] also performs well (96% accuracy, 28 seconds). Support vector machine [39,42,43] and Homer (Hierarchy Of Multi-labEl leaRners) [47] are only slightly less accurate (about 95%), but significantly slower (from 13 to 90 minutes), and they are followed by random forest [37] with about 94% accuracy and between 1 and 44 minutes run time.

We have thus used throughout this work the BRkNN [45] nearest neighbours implementation (as in our previous work on predicting Enzyme Commission classes [9]), using the implementation available in the Mulan software library version 1.4 [46]. The nearest neighbours algorithm also provides an immediate visual representation of the clustering of the protein labels and their attributes. BRkNN is a multi-label adaptation of the classic k-Nearest Neighbour algorithm. The best parametrisation for the data is *k* = 1, that is, only the closest ring of neighbour instances are used to predict the label of an instance. This suggests a pattern of local similarity among the instances causing efficient but local learning. Our ml2db Java code uses queries to generate a Mulan datafile from MySQL database. A Mulan datafile consists of an XML file for the class labels and a Weka ARFF (Attribute Relation File Format) file for the protein instances and their attributes. Where possible, a sparse ARFF format, parsimonious of disk space and computational power, was used. This was possible for the *InterPro*, *CSA* and *InterPro+CSA* attribute sets, given that most attribute values are zero for these attributes (most signatures have no match in a given sequence).

We present results produced using the Euclidean distance in the chosen attribute space. Instances with exactly the same attribute set will have distance 0 (for example, two proteins having exactly the same InterPro features, if the attribute set of choice is *InterPro* signatures). If the instances differ in one attribute they will have a distance of one; if the two instance differ in *x* attributes, they will have a distance of . The Jaccard distance [52] was also used but produces slightly worse accuracy (data not shown).

### Evaluation

Due to the limited number of examples available, we performed leave-one-out validation on the *mechanism* dataset (*n*-fold cross-validation with *n* equal to the number of instances). In short, we trained on all proteins but one, predicted the mechanism for the omitted protein, and then compared the predicted label(s) with the protein’s true label(s). Considering the known shortcomings of leave one out validation (causing high variance when few instances are available for each class label [53]), in a second experiment the entire *mechanism* dataset has also been used for training followed by testing on the *negative* set to examine the false positive cases in more detail. Also, the *mechanism* dataset together with all the non-enzymes in Swiss-Prot (*swissprot-non-EC* set) have been used in two-fold cross validation.

To compare the predictive strength of the various attribute sets, we present the average value of the *classification accuracy* (also called subset accuracy), a strict measure of prediction success, as it requires the predicted set of class labels to be an *exact match* of the true set of labels [49]:

(1) $$\text{Classification Accuracy}(h,D)=\frac{1}{\left|D\right|}\overset{\left|D\right|}{\underset{i=1}{\sum }}I(Z_{i}=Y_{i})$$

where *I*(*true*) = 1, *I*(*false*) = 0 and *D* is a dataset with |*D*| multi-label examples (proteins), each with a set *Y*_*i*_ of labels (enzyme mechanisms) taken from the set of all labels (MACiE mechanisms) *L*: (*x*_*i*_,*Y*_*i*_), *i* = 1…|*D*|, *Y*_*i*_ ⊆ *L*. If we define as *Z*_*i*_ the set of mechanisms predicted by the model *h* (for example the BRkNN classifier or direct assignment rule) for the *i*^*th*^ protein (*x*_*i*_): *Z*_*i*_ = *h*(*x*_*i*_), then the classification accuracy represents the percentage of proteins for which the model predicted the *true, whole set* of mechanisms.

We also report *micro* and *macro* metrics (precision and recall) for completeness since the mechanism classes are long tail distributed. Consider a binary evaluation measure *M*(*TP*,*FP*,*TN*,*FN*) that is calculated based on the number of true positives (*TP*), false positives (*FP*), true negatives (*TN*), and false negatives (*FN*), such as $\text{Precision}=\frac{\text{TP}}{\text{TP}+\text{FP}}$ or $\text{Recall},\text{Sensitivity}=\frac{\text{TP}}{\text{TP}+\text{FN}}$. Let *TP*_*λ*_, *FP*_*λ*_, *TN*_*λ*_ and *FN*_*λ*_ be the number of TP, FP, TN and FN after binary evaluation for a label *λ*. The macro-averaged and micro-averaged versions of measure *M* become:

(2) $$M_{\text{macro}}=\frac{1}{\left|L\right|}\overset{\left|L\right|}{\underset{\lambda =1}{\sum }}M({\text{TP}}_{\lambda },{\text{FP}}_{\lambda },{\text{TN}}_{\lambda },{\text{FN}}_{\lambda })$$

(3) $$M_{\text{micro}}=M\left(\overset{\left|L\right|}{\underset{\lambda =1}{\sum }}{\text{TP}}_{\lambda },\overset{\left|L\right|}{\underset{\lambda =1}{\sum }}{\text{FP}}_{\lambda },\overset{\left|L\right|}{\underset{\lambda =1}{\sum }}{\text{TN}}_{\lambda },\overset{\left|L\right|}{\underset{\lambda =1}{\sum }}{\text{FN}}_{\lambda }\right)$$

In this context *micro* averaging (averaging over the entire confusion matrix) favours more frequent mechanisms, while *macro* averaging gives equal relevance to both rare and frequent mechanism classes. Hence a protein will affect the macro-averaged metrics more if it belongs to a rare mechanism. Micro and macro *specificity* are not presented because these metrics never fall below 99.7%. For binary classification, $\text{Specificity}=\frac{\text{TN}}{\text{FP}+\text{TN}}$, hence, because of the hundreds of possible mechanism labels, most prediction methods provide a very high proportion of true negatives in comparison with false positives, making specificity very close to 100% for any reasonable method and thus not particularly informative. All metrics are further defined and discussed in [46,49,54]. The best achievable value of all these measures is 100% when all instances are correctly classified.

### Software code and graph layout

All experiments were run under a Linux operating system (Ubuntu 12.04 Precise Pangolin) using Oracle Java version 1.7, Python 2.7 and MySQL 5.5. All the Java code (ml2db) and data files used in this paper are available online at http://sourceforge.net/projects/ml2db/ and as Additional file 2 (code) and Additional file 3 (ARFF and XML data files). The full MySQL database dump of all the data and results is available on request. The graphs in Additional file 4 and Additional file 5 have been generated with PyGraphviz, a Python programming language interface to the Graphviz graph layout and visualization package, coded by Aric Hagberg, Dan Schult and Manos Renieri.

## Results

### Data statistics

**Figure 1:**
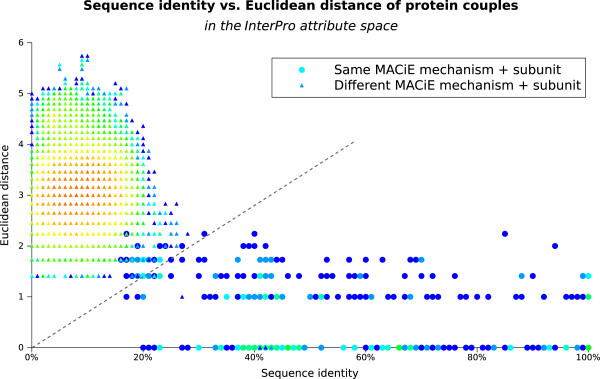
**The sequence identity and Euclidean distance of enzymes with the same and different mechanism.** The diagram presents, for every pair of proteins in the *mechanism* + *negative* datasets, the percentage of identity between the two proteins’ sequences and also the Euclidean distance between their signature sets (in the *InterPro* attribute space). Protein couples having the same MACiE mechanism are represented as circles, while those with different MACiE mechanisms as triangles. The colour scale is logarithmic increasing from blue (for one instance) to light blue (2-3 instances), green (4-9), yellow (70-100), orange (250) and red (up to 433 instances) and represents the number of protein couples having that sequence identity and Euclidean distance. The dashed grey line shown, with equation Euclidean distance = 7 × sequence identity, separates most same-mechanism couples (on its right) from an area dense with different-mechanism couples on its left.

Table 1 summarises the composition of the data sets used in terms of number of instances, attributes and class labels. As already described in the Methods section, each sequence in the *mechanism* + *negative* dataset (all the available MACiE mechanism annotations) was aligned with every other sequence and the percentage of sequence identity calculated. The resulting 126,499 couples are presented in Figure 1, which provides an overview of the sequence identity and Euclidean distance (in the *InterPro* attribute space) for each protein couple. As expected, most protein couples have low sequence identity (between 0% and 30%) and Euclidean distance between two and four, that is, have between four and sixteen differences in their InterPro signatures. This area seems to represent a very frequent sequence distance for protein couples with different function (triangle markers), but also contains a few couples of enzymes having the same mechanism (circle markers).

**Table 1:** Datasets statistics

***Dataset***	***Instances***	***Attributes***	***Class labels***
*Mechanism* set with CSA	248	134	82
*Mechanism* set with Maximum sequence identity	248	82	82
*Mechanism* set with Minimum Euclidean distance (InterPro)	248	82	82
*Mechanism* set with InterPro + CSA	248	456	82
*Mechanism* set with Max seq. Id. + min Eucl. Dist. (InterPro)	248	162	82
*Mechanism* set with all InterPro sub-signature matches	248	743	82
*Mechanism* set with InterPro signatures	248	322	82
*Negative* set with InterPro attributes	290	917	290
*Mechanism* set + Swiss-Prot non-EC with InterPro attributes	35,171	4418	82
*Swiss-Prot non-EC* set with InterPro attributes	68,667 (226,213)	4,825	0

The table presents the number of instances (proteins), attributes (signatures or sequence identity values) and class values (mechanisms) for the datasets used in this work; for the *swissprot-non-EC* set we present the instances that need prediction (the ones sharing a signature with the *mechanism* set), while the total number of instances is shown between parentheses.

The figure shows how enzymes having different mechanisms (triangle markers) concentrate in the upper left area of the plot, mostly having both low sequence identity (<30%) and high Euclidean distance between their signature sets (1.4 to 6, between 2 and 36 different signatures). In contrast, enzymes having the same mechanism form a long band across the figure, showing an extensive range of sequence identity, from about 18% to 100% but a lower and less varied Euclidean distance (0 to 2.2, that is, from having the same signatures to having 5 different signatures).

### Mechanism prediction from sequence identity and Euclidean distance

Using the data in Figure 1 we evaluated whether a simple line separator could tell when a protein has the same label as another protein. To evaluate this simple form of learning (binary predictions in the form "same mechanism" or "different mechanism") we used a line passing through the origin and we varied the angle of the line between zero and ninety degrees, recording the number of correct and incorrect predictions for each line. As it is often the case, there is no absolute best line, some maximise precision, others recall. However, to give an example, the line passing through the origin with equation: *Euclidean distance* = 7 × *sequence identity* provides a recall of 93.5%, while still conserving an accuracy of 99.8% and a precision of 93.2%. For this binary case accuracy is calculated with the usual formula $\frac{\text{TP}+\text{TN}}{\text{TP}+\text{FP}+\text{TN}+\text{FN}}$, precision is $\frac{\text{TP}}{\text{TP}+\text{FP}}$, and recall (or sensitivity) is $\frac{\text{TP}}{\text{TP}+\text{FN}}$.

Another way to read the equation *Euclidean distance* = 7 × *sequence identity* is that for two proteins differing in two signatures, at least about 20% sequence identity is necessary for the proteins to have the same mechanism (about 25% sequence identity for three differences, 29% for four differences and so on). In addition, while the equation suggests that proteins having exactly the same signatures can have any level of sequence identity, in practice the sequence identity for couples having the same mechanism never falls below 18% in the data, possibly because two random sequences (of approximately the same length as our sequences) will have a minimum number of identical amino acids by chance alone. The couples having the same mechanism are almost homogeneously scattered above this 18% threshold, but with several couples having about 40% sequence identity and few having very high sequence identity (80% to 100%). The same result structure holds when sequence similarity is used instead of sequence identity (data not shown).

### Mechanism prediction with InterPro and Catalytic Site Atlas sequence attributes

**Figure 2:**
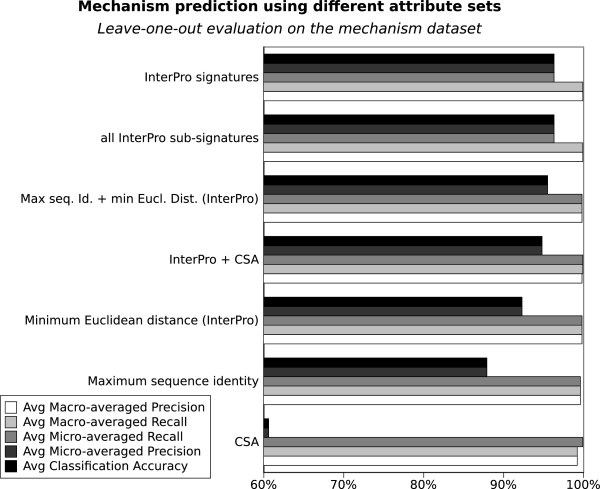
**Predicting mechanism using InterPro and Catalytic Site Atlas attributes.** A comparison of the predictive performance of various sets of attributes in a leave one out evaluation of the *mechanism* dataset. The x axis starts at 60% to better highlight the small differences between the top methods.

In this section we use machine learning (k-Nearest Neighbour) to compare the ability of InterPro signatures and Catalytic Site Atlas (CSA) matches to predict enzyme mechanism on the basic *mechanism* dataset. Figure 2 presents an overview of the performance of different set of attributes in predicting the *mechanism* dataset. As an indicative baseline for prediction we used the labels predicted when mechanism is assigned simply by the presence of a certain set of InterPro domains (*InterProdirect transfer*). For example, protein ODPB_GEOSE of *Geobacillus stearothermophilus* (pyruvate dehydrogenase E1 component subunit beta, P21874) is part of the dataset and has MACiE mechanism M0106 (pyruvate dehydrogenase) and InterPro IPR005475, IPR005476, IPR009014 and IPR015941. Hence, if we use direct transfer of mechanism labels, another protein such as ODBB_HUMAN (2-oxoisovalerate dehydrogenase subunit beta mitochondrial, P21953) which has *exactly the same InterPro signatures* will receive a M0106 label, thereby introducing an error, since ODBB_HUMAN’s mechanism is in fact M0280 (or 3-methyl-2-oxobutanoate dehydrogenase). If several proteins in the training set have exactly the same InterPro attributes, the given test protein will be assigned all of their mechanism labels. The direct transfer method achieves 99.9% accuracy and 95.7% precision on the *mechanism* set, but only 76.6% recall. That is, when it assigns a label, it tends to be correct, but about a quarter of the proteins do not find another protein with exactly the same InterPro signatures in the training set, and so do not receive a prediction. The low recall is thus mainly caused by false negatives.

If we use the BRkNN algorithm instead, as described in the Methods section, Figure 2 shows that *InterPro* attributes alone are very good predictors of mechanism and achieve 96.3% classification accuracy and micro-averaged precision, and with a 99.9% macro-averaged recall. Using all InterPro signatures (including the so called "non-integrated" signatures) does not significantly improve nor degrade the overall *InterPro* result. *CSA* attributes are significantly worse than *InterPro* attributes at predicting mechanism on this dataset (60.6% classification accuracy and micro-averaged precision, 99.2% macro-averaged recall). Combining CSA attributes with InterPro attributes (*InterPro+CSA* attribute set) causes a slight degradation compared with using *InterPro* alone, achieving only 94.8% accuracy.

### Mechanism prediction from three-dimensional structure

**Figure 3:**
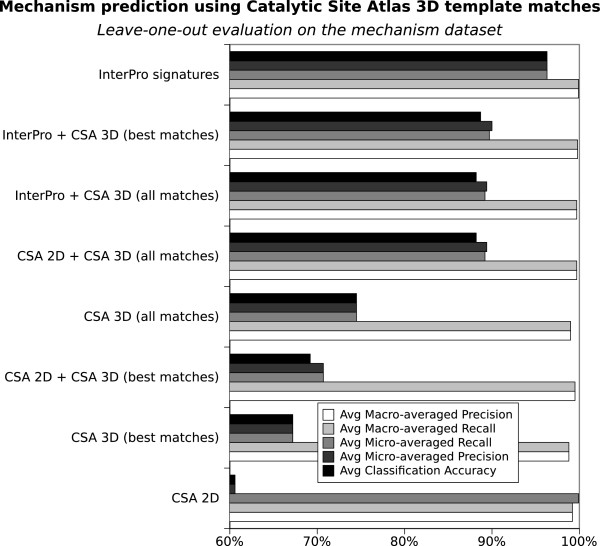
**Predicting mechanism using Catalytic Site Atlas 3D attributes.** A comparison of the predictive performance of various sets of sequence based (2D) and structure based (3D) Catalytic Site Atlas attributes in a leave one out evaluation of the *mechanism* dataset. The x axis starts at 60%.

Figure 3 presents an evaluation of predicting mechanism using Catalytic Site Atlas 3D template matches (CSA 3D), either alone or in combination with sequence based attributes. We note that CSA 3D attributes appear more accurate than CSA sequence attributes (CSA 2D) and that the integration of CSA sequence and 3D attributes generally improves prediction compared with using CSA 2D or CSA 3D alone. However, adding CSA 3D attributes to InterPro attributes does not provide an advantage and indeed degrades prediction.

The predictions based on CSA 3D templates mainly suffer from lack of coverage. The method generally predicts well, with few false positives, but it produces a high number of false negatives. This limitation is partly overcome by using all possible matches instead of only best matches (see Figure 3), but at the current state the method still appears to be less accurate than InterPro based methods. However, the current extension of CSA to CSA 2.0 [31], and any future extension in the number of 3D templates may improve its performance.

### Statistical significance of the results

In order to define whether a set of attributes is a significantly better predictor than another set, we can imagine a random machine with characteristics similar to one of our predictors. Let us consider a method that emits either correct predictions with probability *P* or incorrect predictions with probability 1 - *P*. This method’s percentage of correct predictions will have mean 100 × *P* and standard deviation $100\sqrt{\frac{P(1-P)}{N}}$. If the machine predicts *N* = 250 protein-class label couples with *P* = 96.3*%* (1 - *P* = 3.7*%*) then the standard deviation equals $100\sqrt{\frac{0.963\times 0.037}{250}}=1.19\%$. We can thus consider results with accuracy between 93.9% to 98.7% as being within two standard deviations and hence *not* significantly different.

### Sequence identity and minimum Euclidean distance

Using only the maximum sequence identities as attributes (the maximum identity of the protein to be predicted when compared with the set of proteins having each mechanism) achieves 87.9% classification accuracy and micro-averaged precision and 99.6% macro-averaged recall. The results moderately improve when the minimum Euclidean distance is used (the minimum distance between the set of InterPro signatures of the protein to be predicted and the signatures of the proteins having each mechanism). The classification accuracy and micro-averaged precision grow from 87.9% to 92.3% and the macro-averaged recall from 99.6% to 99.8%. But it is the combination of the maximum sequence identity and minimum Euclidean distance that provides the best results within this style of data schema, with classification accuracy and micro-averaged precision reaching 95.5% while the macro-averaged recall remains at 99.8%. These results are not significantly worse than the results achieved by simply using InterPro signatures, but the method is much more computationally intensive.

### Testing on negative sets

Here we assess the predictive performance of the best method (*InterPro* attributes + k-Nearest Neighbour) on a separate test set and we examine the type of false positive mistakes that the method produces. We use here the *negative* set, which contains 290 enzymes with known MACiE labels, but impossible to use for cross validation as they have only one protein per label. We thus train on the *mechanism* set plus the non-enzymes in Swiss-Prot (*swissprot-non-EC*), to provide training examples for both proteins having and not having the mechanisms of interest and we test on the separate *negative* set. If the method behaved in an ideal way, all the enzymes in the *negative* set would be predicted to be without labels, because none of the labels available in the training set is appropriate for the *negative* enzymes.

We also randomly partition the *mechanism* dataset into two folds (*mech-fold1* and *mech-fold2*). Because many mechanisms in the *mechanism* set only have two proteins, we could not generate more than two folds without causing a further loss of mechanism labels and proteins. When training on fold 1 (*mech-fold1* + half of *swissprot-non-EC*) and testing on fold 2 (*mech-fold2* + the other half of *swissprot-non-EC*) there are only twelve false positive and twenty-three false negative predictions. Reversing the folds causes only six false positive and twenty-one false negative predictions. Thus even in such a vast test set, the *mechanism* training set only generates eighteen false positive predictions over more than 220,000 proteins. In addition, many of these false predictions are indeed very close to the mark. For example, *Canis familiaris*’ Inactive Pancreatic Lipase-related Protein 1 (LIPR1_CANFA, P06857) is predicted as having MACiE mechanism M0218_pancreatic_lipase. In fact, as recorded in Swiss-Prot’s annotation, this protein was originally thought to be a pancreatic lipase [55,56], but has been shown to lack lipase activity [57]. The same is true for the inactive pancreatic lipase-related proteins of *Homo sapiens*, *Mus musculus* and *Rattus norvegicus* which are also all predicted as M0218_pancreatic_lipase (UniProt accessions LIPR1_HUMAN/P54315, LIPR1_MOUSE/Q5BKQ4 and LIPR1_RAT, P54316 respectively). The method also predicts *Legionella pneumophila*’s Protein DlpA (DLPA_LEGPH, Q48806) as citrate synthase (MACiE M0078), and the protein is in fact highly related to the citrate synthase family, but lacks the conserved active His at position 264 which is replaced by an Asn residue.

## Discussion

### Sequence identity and Euclidean distance

The good accuracy, precision and recall obtained by the method are very encouraging but also highlight how similar in sequence many of the proteins belonging to one MACiE code are (as shown in Figure 1). This might be caused by strong conservation of many of these essential enzymes or, more prosaically, by a conservative manual annotation, which favours the transfer of labels among closely related orthologs. The consequence is a trusted but unchallenging data set for the methods presented.

In addition, even the performance of a simple line partition is reasonably high, provided that the Euclidean distance in the InterPro attributes space is used to further separate proteins, confirming the importance of using sequence signatures in addition to measures of sequence identity or similarity. Concluding, the InterPro based data schema seems to be essential to the good performance of: 1. machine learning over a sparse matrix (as presented using the k-Nearest Neighbour algorithm), 2. machine learning over a full matrix of sequence identity and Euclidean distance and even 3. simple regression (for example using the lines Euclidean distance = n × sequence identity).

At the current state of annotation, the small size of the training set makes the *minimum Euclidean distance* method look like a possible option for prediction. It is important to note though that a significant growth of the test or training sets will make a system based on alignments used to calculate the sequence identity (plus Euclidean distance calculation) much more computationally intensive than a machine learning algorithm (such as nearest neighbours) which relies on Euclidean distance alone.

### Prediction quality

Additional file 4 is a graph of all enzymes in the *mechanism* dataset with their *InterPro* attributes and MACiE mechanism. The graph clearly shows that most clusters (proteins sharing a number of signatures) only have one MACiE mechanism, making predictions by k-Nearest Neighbour reasonably straightforward, as confirmed by the high accuracy, precision and recall of the leave one out evaluation on the *mechanism* dataset.

In fact, no false positive predictions appear when training on the *negative* dataset and testing on the *mechanism* dataset, but a small number of false positives (sixteen) appear when training on the *mechanism* set and testing on the *negative*, as shown in Table 2, which summarises the prediction errors for the training and testing evaluation experiments presented (a full list of the individual predictions can be found in Additional file 6).

**Table 2:** Prediction statistics

***Training set***	***Test set***	***False positive***	***False negative***
*mechanism* +*swissprot-non-EC* fold 1	*mechanism* +*swissprot-non-EC* fold 2	12	23
*mechanism* +*swissprot-non-EC* fold 2	*mechanism* +*swissprot-non-EC* fold 1	6	21
*mechanism*	*negative*	16	n/a
*negative*	*mechanism*	0	n/a

This table presents the number of false positive and false negative predictions for the training + testing evaluations (using *InterPro* attributes), a detailed list of the predictions is available in Additional file 6.

Additional file 5 contains a graph showing these sixteen false positive predictions in more detail. The clusters graphically show which protein neighbours caused the misprediction, and the signatures that these proteins share with the falsely predicted protein. For example, protein PABB_ECOLI has mechanism M0283: aminodeoxychorismate synthase (shown as a green oval), but it is predicted as M0314_component1_I: anthranilate synthase instead (shown as a red oval). The causes of the misprediction are signatures Anth_synth_I, ADC_synthase, Chorismate-bd_C and Anth_synth_I_N, which protein PABB_ECOLI shares with two anthranilate synthases (TRPE_SERMA and TRPE_SULSO). The two protein families are in fact very similar and the reactions’ Enzyme Commission numbers only differ in the last (substrate) digit: anthranilate synthase has EC 4.1.3.27 and aminodeoxychorismate lyase has EC 4.1.3.38.

One way to tease out the influence of these signatures that overlap across families is to introduce a larger sample of "negative" sequences. And this is what has been done in the two-fold cross evaluation experiment (*mechanism* plus *swissprot-non-EC* data sets). And, indeed, adding the non-enzymes keeps the number of false positive predictions extremely low (only 18 over 226,213 non-enzymes), but the split also somehow dilutes the informative signal of the *mechanism* dataset, causing a slightly larger number of false negative predictions (44 over 248 proteins).

Hence, in general, the methods seem to perform well. For the use of enzyme researchers we thus provide a list of all mechanism predictions for all Swiss-Prot enzymes (proteins having an Enzyme Commission number) as Additional file 7.

To provide correct neighbours for the instances currently receiving false negative or false positive predictions we would need to have either additional, more specific signatures in the set, or more proteins with the same signatures as the available instances. A detail of note is that the two best methods (*InterPro* and *maximum sequence identity*) label different proteins as false positives and false negatives. Hence by combining the predictions of the two methods (that is, accepting a label even if only one of the methods predicts it) we could reduce the number of false negatives to zero, but the number of false positive predictions would remain the same.

## Conclusions

The machine learning method proposed can be applied to any sequenced protein and can assign a mechanism that cannot be immediately inferred from the InterPro signatures present in the sequence.

As future work it would be of interest to compare this approach with other representations of proteins, for example as discussed in [58] where protein sequences are described by fixed-length patterns with allowance for mutations, and the resulting mismatch string kernel is used with support vector machines to detect remote homology. These or other sequence features could be learned directly using a nearest neighbours algorithm or used as a kernel matrix for a support vector machine classifier, using a publicly available library such as libSVM which also allows for multi-label predictions.

The method presented is currently limited only by the lack of available data. Only 335 mechanisms have been described in detail in MACiE, the richest publicly available mechanism database, out of the more than 4,000 existing fourth level Enzyme Commission numbers, each of which could have one or more different mechanisms existing in nature. And only 540 proteins have been annotated with a specific MACiE mechanism. Additionally, most mechanisms only have one protein exemplar annotated within the MACiE, SFLD or EzCatDb databases, and cannot therefore be used for cross-validation.

Further validation will be needed when the dataset has grown, to clarify whether the best and fastest method remains the one we identified (*InterPro* attributes with k-Nearest Neighbour). However, the general indication is that mechanism prediction through sequence is possible, quick, accurate and produces a very limited number of false positives (just 0.00007% of 226,213 proteins) setting the foundations for further improvements to the methodology.

## Competing interests

The authors declare that they have no competing interests.

## Authors’ contributions

LDF and JBOM designed the work and analysed the results. LDF collected the data, ran the experiments and wrote the initial draft of the manuscript. Both authors read and approved the final manuscript.

## Supplementary Material

Additional file 1 **Comparison of machine learning algorithms.** Additional file comparison_of_machine_learning_algorithms.csv contains several evaluation metrics and run time for various machine learning algorithms when executing a leave-one-out experiment on the *mechanism* dataset. Click here for file

Additional file 2 **Java code of ml2db.** Additional file ml2db_code.tar.gz contains the Java source code to run the multi-label machine learning experiments and save the results to database. The code’s Javadoc is included. Click here for file

Additional file 3 **ARFF and XML data files.** Additional file arff_xml_files.tar.gz contains the ARFF and XML data files used in the machine learning experiments presented. Click here for file

Additional file 4 **Neighbours clusters in the the mechanism dataset.** Additional file mechanism_set_neighbours.pdf is a graph of all enzymes in the *mechanism* dataset with *InterPro* attributes. The proteins are shown as blue squares (containing their UniProt entry name) connected to their signatures (black ovals containing the InterPro signature short name) and their mechanisms (green ovals containing the MACiE entry number and name). The graph was generated with PyGraphviz, a Python interface to Graphviz. Click here for file

Additional file 5 **Neighbours clusters of the false positive predictions when training on the** ***mechanism* set and testing on the** ***negative* set.** Additional file graph_training_on_mechanism_testing_on_negative.pdf is a graph showing as red squares the proteins’ false positive labels when training on the mechanism set and testing on the negative set (using InterPro attributes, shown here as green rectangles). The green ovals represent the protein’s true mechanism, while the red ovals are the mistaken predictions. The yellow squares are neighbour proteins (proteins sharing some of the attributes) which caused the misprediction. The graph was generated with PyGraphviz, a Python interface to Graphviz. Click here for file

Additional file 6 **List of false positive and false negative predictions.** Additional file false_positive_and_negative_predictions.csv contains a comma separated set of tables with one row for each false positive or false negative prediction in the main training-testing experiments. The tables include each protein’s UniProt accession, entry name and species and the true and predicted mechanism identifiers and description (where relevant). Click here for file

Additional file 7 **Mechanism predictions for all UniProt Swiss-Prot enzymes.** Additional file swissprot_enzymes_mechanism_predictions.csv.tar.gz contains a comma separated file of the mechanism predictions for all Swiss-Prot enzymes (proteins having an Enzyme Commission number). The training set used for prediction includes all mechanism labelled proteins (the *mechanism* and *negative* sets) and all Swiss-Prot non-enzymes (the *swissprot-non-EC* set) with their InterPro attributes and MACiE mechanism class labels. The predicted mechanism is presented alongside the protein’s name, organism and Enzyme Commission number(s) taken from UniProt. Click here for file

## References

[B1] Holliday GL Bartlett GJ Almonacid DE O’Boyle NM Murray-Rust P Thornton JM Mitchell JBO MACiE: a database of enzyme reaction mechanisms Bioinformatics 2005 21 23 4315 4316 [ http://dx.doi.org/10.1093/bioinformatics/bti693] 10.1093/bioinformatics/bti693 16188925 PMC2748267

[B2] Holliday GL Almonacid DE Bartlett GJ O’Boyle NM Torrance JW Murray-Rust P Mitchell JBO Thornton JM MACiE (mechanism, annotation and classification in enzymes): novel tools for searching catalytic mechanisms Nucleic Acids Res 2007 35 Database issue D515 D520 [ http://dx.doi.org/10.1093/nar/gkl774] 17082206 10.1093/nar/gkl774 PMC1634735

[B3] Holliday GL Andreini C Fischer JD Rahman SA Almonacid DE Williams ST Pearson WR MACiE: exploring the diversity of biochemical reactions Nucleic Acids Res 2012 40 Database issue D783 D789 [ http://dx.doi.org/10.1093/nar/gkr799] 22058127 10.1093/nar/gkr799 PMC3244993

[B4] Hunter S Jones P Mitchell A Apweiler R Attwood TK Bateman A Bernard T Binns D Bork P Burge S de Castro E Coggill P Corbett M Das U Daugherty L Duquenne L Finn RD Fraser M Gough J Haft D Hulo N Kahn D Kelly E Letunic I Lonsdale D Lopez R Madera M Maslen J McAnulla C McDowall J InterPro in 2011: new developments in the family and domain prediction database Nucleic Acids Res 2012 40 Database issue D306 D312 [ http://dx.doi.org/10.1093/nar/gkr948] 22096229 10.1093/nar/gkr948 PMC3245097

[B5] Porter CT Bartlett GJ Thornton JM The Catalytic Site Atlas: a resource of catalytic sites and residues identified in enzymes using structural data Nucleic Acids Res 2004 32 Database issue D129 D133 [ http://dx.doi.org/10.1093/nar/gkh028] 14681376 10.1093/nar/gkh028 PMC308762

[B6] Rose PW Bi C Bluhm WF Christie CH Dimitropoulos D Dutta S Green RK Goodsell DS Prlic A Quesada M Quinn GB Ramos AG Westbrook JD Young J Zardecki C Berman HM Bourne PE The RCSB protein data bank: new resources for research and education Nucleic Acids Res 2013 41 Database issue D475 D482 [ http://dx.doi.org/10.1093/nar/gks1200] 23193259 10.1093/nar/gks1200 PMC3531086

[B7] Cai CZ Han LY Ji ZL Chen X Chen YZ SVM-Prot: Web-based support vector machine software for functional classification of a protein from its primary sequence Nucleic Acids Res 2003 31 13 3692 3697 10.1093/nar/gkg600 12824396 PMC169006

[B8] Cai CZ Han LY Ji ZL Chen YZ Enzyme family classification by support vector machines Proteins 2004 55 66 76 [ http://dx.doi.org/10.1002/prot.20045] 10.1002/prot.20045 14997540

[B9] De Ferrari L Aitken S van Hemert J Goryanin I EnzML: Multi-label prediction of enzyme classes using InterPro signatures BMC Bioinformatics 2012 13 61 10.1186/1471-2105-13-61 22533924 PMC3483700

[B10] Traube T Vijayakumar S Hirsch M Uritsky N Shokhen M Albeck A EMBM - a new enzyme mechanism-based method for rational design of chemical sites of covalent inhibitors J Chem Inf Model 2010 50 12 2256 2265 [ http://dx.doi.org/10.1021/ci100330y] 10.1021/ci100330y 21090595 PMC3010454

[B11] Choi K Kim S Sequence-based enzyme catalytic domain prediction using clustering and aggregated mutual information content J Bioinform Comput Biol 2011 9 5 597 611 10.1142/S0219720011005677 21976378

[B12] Chea E Livesay DR: How accurate and statistically robust are catalytic site predictions based on closeness centrality? BMC Bioinformatics 2007 8 153 [ http://dx.doi.org/10.1186/1471-2105-8-153] 10.1186/1471-2105-8-153 17498304 PMC1876251

[B13] Mistry J Bateman A Finn RD Predicting active site residue annotations in the Pfam database BMC Bioinformatics 2007 8 298 [ http://dx.doi.org/10.1186/1471-2105-8-298] 10.1186/1471-2105-8-298 17688688 PMC2025603

[B14] Nagano N EzCatDB: the enzyme catalytic-mechanism database Nucleic Acids Res 2005 33 Database issue D407 D412 [ http://dx.doi.org/10.1093/nar/gki080] 15608227 10.1093/nar/gki080 PMC540034

[B15] Brown S Babbitt P Using the structure-function linkage database to characterize functional domains in enzymes Curr Protoc Bioinformatics 2006 Chapter 2 Unit 2.10 [ http://dx.doi.org/10.1002/0471250953.bi0210s13] 18428763 10.1002/0471250953.bi0210s13

[B16] Consortium U Update on activities at the universal protein resource (UniProt) in 2013 Nucleic Acids Res 2013 41 Database issue D43 D47 23161681 10.1093/nar/gks1068 PMC3531094

[B17] Artimo P Jonnalagedda M Arnold K Baratin D Csardi G de Castro E Duvaud S Flegel V Fortier A Gasteiger E Grosdidier A Hernandez C Ioannidis V Kuznetsov D Liechti R Moretti S Mostaguir K Redaschi N Rossier G Xenarios I Stockinger H ExPASy: SIB bioinformatics resource portal Nucleic Acids Res 2012 40 Web Server issue W597 W603 [ http://dx.doi.org/10.1093/nar/gks400] 22661580 10.1093/nar/gks400 PMC3394269

[B18] Bernstein FC Koetzle TF Williams GJ Meyer EF Brice MD Rodgers JR Kennard O Shimanouchi T Tasumi M The protein data bank: a computer-based archival file for macromolecular structures J Mol Biol 1977 112 3 535 542 10.1016/S0022-2836(77)80200-3 875032

[B19] Mulder N Apweiler R InterPro and InterProScan: tools for protein sequence classification and comparison Methods Mol Biol 2007 396 59 70 10.1007/978-1-59745-515-2_5 18025686

[B20] Lees JG Lee D Studer RA Dawson NL Sillitoe I Das S Yeats C Dessailly BH Rentzsch R Orengo CA Gene3D: Multi-domain annotations for protein sequence and comparative genome analysis Nucleic Acids Res 2014 42 D240 D245 [ http://dx.doi.org/10.1093/nar/gkt1205] 10.1093/nar/gkt1205 24270792 PMC3965083

[B21] Pedruzzi I Rivoire C Auchincloss AH Coudert E Keller G de Castro E Baratin D Cuche BA Bougueleret L Poux S Redaschi N Xenarios I Bridge A Consortium U HAMAP in 2013, new developments in the protein family classification and annotation system Nucleic Acids Res 2013 41 Database issue D584 D589 23193261 10.1093/nar/gks1157 PMC3531088

[B22] Mi H Muruganujan A Thomas PD PANTHER in 2013: modeling the evolution of gene function, and other gene attributes, in the context of phylogenetic trees Nucleic Acids Res 2013 41 Database issue D377 D386 [ http://dx.doi.org/10.1093/nar/gks1118] 23193289 10.1093/nar/gks1118 PMC3531194

[B23] Finn RD Bateman A Clements J Coggill P Eberhardt RY Eddy SR Heger A Hetherington K Holm L Mistry J Sonnhammer ELL Tate J Punta M Pfam: the protein families database Nucleic Acids Res 2014 42 D222 D230 [ http://dx.doi.org/10.1093/nar/gkt1223] 10.1093/nar/gkt1223 24288371 PMC3965110

[B24] Nikolskaya AN Arighi CN Huang H Barker WC Wu CH PIRSF family classification system for protein functional and evolutionary analysis Evol Bioinform Online 2006 2 197 209 19455212 PMC2674652

[B25] Attwood TK Coletta A Muirhead G Pavlopoulou A Philippou PB Popov I Romá-Mateo C Theodosiou A Mitchell AL The PRINTS database: a fine-grained protein sequence annotation and analysis resource–its status in 2012 Database (Oxford) 2012 2012 bas019 [ http://dx.doi.org/10.1093/database/bas019] 22508994 10.1093/database/bas019 PMC3326521

[B26] Bru C Courcelle E CarrÃĺre S Beausse Y Dalmar S Kahn D The ProDom database of protein domain families: more emphasis on 3D Nucleic Acids Res 2005 33 Database issue D212 D215 [ http://dx.doi.org/10.1093/nar/gki034] 15608179 10.1093/nar/gki034 PMC539988

[B27] Sigrist CJA de Castro E Cerutti L Cuche BA Hulo N Bridge A Bougueleret L Xenarios I New and continuing developments at PROSITE Nucleic Acids Res 2013 41 D1 D344 D347 [ http://dx.doi.org/10.1093/nar/gks1067] 10.1093/nar/gks1067 23161676 PMC3531220

[B28] Letunic I Doerks T Bork P SMART 7: recent updates to the protein domain annotation resource Nucleic Acids Res 2012 40 Database issue D302 D305 [ http://dx.doi.org/10.1093/nar/gkr931] 22053084 10.1093/nar/gkr931 PMC3245027

[B29] Gough J Karplus K Hughey R Chothia C Assignment of homology to genome sequences using a library of hidden Markov models that represent all proteins of known structure J Mol Biol 2001 313 4 903 919 [ http://dx.doi.org/10.1006/jmbi.2001.5080] 10.1006/jmbi.2001.5080 11697912

[B30] Haft DH Selengut JD Richter RA Harkins D Basu MK Beck E TIGRFAMs and genome properties in 2013 Nucleic Acids Res 2013 41 Database issue D387 D395 [ http://dx.doi.org/10.1093/nar/gks1234] 23197656 10.1093/nar/gks1234 PMC3531188

[B31] Furnham N Holliday GL de Beer TAP Jacobsen JOB Pearson WR Thornton JM The Catalytic Site Atlas 2.0: cataloging catalytic sites and residues identified in enzymes Nucleic Acids Res 2014 42 Database issue D485 D489 [ http://dx.doi.org/10.1093/nar/gkt1243] 24319146 10.1093/nar/gkt1243 PMC3964973

[B32] Barker JA Thornton JM An algorithm for constraint-based structural template matching: application to 3D templates with statistical analysis Bioinformatics 2003 19 13 1644 1649 10.1093/bioinformatics/btg226 12967960

[B33] Laskowski RA Watson JD Thornton JM ProFunc: a server for predicting protein function from 3D structure Nucleic Acids Res 2005 33 Web Server issue W89 W93 [ http://dx.doi.org/10.1093/nar/gki414] 15980588 10.1093/nar/gki414 PMC1160175

[B34] Rice P Longden I Bleasby A EMBOSS: the European molecular biology open software suite Trends Genet 2000 16 6 276 277 10.1016/S0168-9525(00)02024-2 10827456

[B35] Needleman SB Wunsch CD A general method applicable to the search for similarities in the amino acid sequence of two proteins J Mol Biol 1970 48 3 443 453 [ http://www.sciencedirect.com/science/article/pii/0022283670900574] 10.1016/0022-2836(70)90057-4 5420325

[B36] Aha D Kibler D Instance-based learning algorithms Mach Learn 1991 6 37 66

[B37] Breiman L Random Forests Mach Learn 2001 45 5 32 10.1023/A:1010933404324

[B38] Fuernkranz J Huellermeier E Loza Mencia E Brinker K Multilabel classification via calibrated label ranking Mach Learn 2008 73 2 133 153 10.1007/s10994-008-5064-8

[B39] Hastie T Tibshirani R Classification by pairwise coupling Advances in Neural Information Processing Systems, Volume 10. Edited by Jordan MI, Kearns MJ, Solla SA 1998 Cambridge, Massachusetts: MIT Press

[B40] Holte RC Very simple classification rules perform well on most commonly used datasets Mach Learn 1993 11 63 90 10.1023/A:1022631118932

[B41] John GH Langley P Estimating Continuous Distributions in Bayesian Classifiers Eleventh Conference on Uncertainty in Artificial Intelligence 1995 San Mateo: Morgan Kaufmann 338 345

[B42] Keerthi SS Shevade SK Bhattacharyya C Murthy KRK Improvements to Platt’s SMO Algorithm for SVM classifier design Neural Comput 2001 13 3 637 649 [ http://dx.doi.org/10.1162/089976601300014493] 10.1162/089976601300014493

[B43] Platt J Fast training of support vector machines using sequential minimal optimization Advances in Kernel Methods - Support Vector Learning. Edited by Schoelkopf B, Burges C, Smola A 1998 Cambridge, Massachusetts: MIT Press [ http://research.microsoft.com/en-us/um/people/jplatt/smo-book.pdf]

[B44] Quinlan R C4.5: Programs for Machine Learning 1993 San Mateo, CA: Morgan Kaufmann Publishers

[B45] Spyromitros E Tsoumakas G Vlahavas I An empirical study of lazy multilabel classification algorithms Artificial Intelligence: Theories, Models and Applications 2008 Berlin Heidelberg: Springer 401 406 [ http://dx.doi.org/10.1007/978-3-540-87881-0_40]

[B46] Tsoumakas G Katakis I Vlahavas I Mining Multi-label Data 2010 US: Springer [ http://mlkd.csd.auth.gr/publication_details.asp?publicationID=290]

[B47] Tsoumakas G Katakis I Vlahavas I Effective and efficient multilabel classification in domains with large number of labels Proc. ECML/PKDD 2008 Workshop on Mining Multidimensional Data (MMD 2008) 2008 30 44

[B48] Tsoumakas G Spyromitros-Xioufis E Vilcek J Vlahavas I MULAN: A Java Library for Multi-Label Learning J Mach Learn Res 2011 12 Jul 2411 2414

[B49] Tsoumakas G Vlahavas I Random k -Labelsets: an ensemble method for multilabel classification 2007 [ http://citeseerx.ist.psu.edu/viewdoc/download?doi=10.1.1.97.5044&rep=rep1&type=pdf]

[B50] Witten IH Frank E Data Mining - Practical machine learning tools and techniques with Java implementations 2005 Morgan Kaufmann: San Francisco

[B51] Zhang ML Zhou ZH Multilabel neural networks with applications to functional genomics and text categorization Knowl Data Eng IEEE Trans on 2006 18 10 1338 1351

[B52] Jaccard P The distribution of the flora in the alpine zone 1 New Phytologist 1912 11 2 37 50 [ http://dx.doi.org/10.1111/j.1469-8137.1912.tb05611.x] 10.1111/j.1469-8137.1912.tb05611.x

[B53] Dekel O Shamir O Multiclass-multilabel classification with more classes than examples Proceedings of the 13 th International Conference on Artificial Intelligence and Statistics (AISTATS) 2010. Volume 9 of JMLR: W&CP 2010 Chia Laguna Resort, Sardinia, Italy 137 144 [ http://machinelearning.wustl.edu/mlpapers/paper_files/AISTATS2010_DekelS10.pdf]

[B54] Sokolova M Lapalme G A systematic analysis of performance measures for classification tasks Inf Process Manag 2009 45 4 427 437 [ http://www.sciencedirect.com/science/article/pii/S0306457309000259] 10.1016/j.ipm.2009.03.002

[B55] Kerfelec B LaForge KS Puigserver A Scheele G Primary structures of canine pancreatic lipase and phospholipase A2 messenger RNAs Pancreas 1986 1 5 430 437 10.1097/00006676-198609000-00007 3562437

[B56] Mickel FS Weidenbach F Swarovsky B LaForge KS Scheele GA Structure of the canine pancreatic lipase gene J Biol Chem 1989 264 22 12895 12901 2502543

[B57] Roussel A de Caro J Bezzine S Gastinel L de Caro A Carrière F Leydier S Verger R Cambillau C Reactivation of the totally inactive pancreatic lipase RP1 by structure-predicted point mutations Proteins 1998 32 4 523 531 10.1002/(SICI)1097-0134(19980901)32:4<523::AID-PROT10>3.0.CO;2-E 9726421

[B58] Leslie CS Eskin E Cohen A Weston J Noble WS Mismatch string kernels for discriminative protein classification Bioinformatics 2004 20 4 467 476 [ http://bioinformatics.oxfordjournals.org/content/20/4/467.abstract] 10.1093/bioinformatics/btg431 14990442

