# Supplementary material for: From sequence to enzyme mechanism using multi-label machine learning
Source: BMC Bioinformatics. 2014 May 19;15:150. doi: 10.1186/1471-2105-15-150 (PMC4229970; doi:10.1186/1471-2105-15-150)
Supplement: Additional file 2 — Java code of ml2db. Additional file ml2db_code.tar.gz contains the Java source code to run the multi-label machine learning experiments and save the results to database. The code’s Javadoc is included. [file 1471-2105-15-150-S2.zip › additional file 2/ml2db/ecmulan/doc/constant-values.html]

Constant Field Values


JavaScript is disabled on your browser.


- Overview
- Package
- Class
- Use
- Tree
- Deprecated
- Index
- Help

- Prev
- Next

- Frames
- No Frames

- All Classes

# Constant Field Values

## Contents

- uk.ac.\*

## uk.ac.\*

- uk.ac.ed.inf.mulanxml.MulanXml

  | Modifier and Type | Constant Field | Value |
  |  |  |  |
  | --- | --- | --- |
  | `public static final java.lang.String` | `LABEL_NAME_ATTRIBUTE` | `"name"` |
  | `public static final java.lang.String` | `LABEL_XML_TAG` | `"label"` |
  | `public static final java.lang.String` | `MULAN_XML_ROOT_TAG` | `"labels"` |
  | `public static final java.lang.String` | `MULAN_XML_ROOT_TAG_ATTRIBUTE_NAME` | `"xmlns"` |
  | `public static final java.lang.String` | `MULAN_XML_ROOT_TAG_ATTRIBUTE_VALUE` | `"http://mulan.sourceforge.net/labels"` |

- uk.ac.ed.inf.mulanxml.ec.EcDbWriter

  | Modifier and Type | Constant Field | Value |
  |  |  |  |
  | --- | --- | --- |
  | `public static final java.lang.String` | `ANCESTOR_FIELD_NAME` | `"ancestor"` |
  | `public static final java.lang.String` | `EC_DATA_TYPE` | `"VARCHAR(13)"` |
  | `public static final java.lang.String` | `EC_FIELD_NAME` | `"ec"` |
- uk.ac.ed.inf.mulanxml.ec.EcNumberGenerator

  | Modifier and Type | Constant Field | Value |
  |  |  |  |
  | --- | --- | --- |
  | `public static final java.lang.String` | `DASH` | `"-"` |
  | `public static final java.lang.String` | `DOT` | `"."` |
  | `public static final int` | `FULL_HIERARCHY_LENGHT` | `4` |
  | `public static final java.lang.String` | `MAX_LEVEL1_CLASS` | `"6"` |
  | `public static final java.lang.String` | `MAX_LEVEL1_REGEXP` | `"[1-6]\\."` |
  | `public static final java.lang.String` | `MAX_LEVEL2_CLASS` | `"99"` |
  | `public static final java.lang.String` | `MAX_LEVEL2_REGEXP` | `"[0-9]{1,2}\\."` |
  | `public static final java.lang.String` | `MAX_LEVEL3_CLASS` | `"99"` |
  | `public static final java.lang.String` | `MAX_LEVEL3_REGEXP` | `"[0-9]{1,2}\\."` |
  | `public static final java.lang.String` | `MAX_LEVEL4_CLASS` | `"999"` |
  | `public static final java.lang.String` | `MAX_LEVEL4_REGEXP` | `"n?[0-9]{1,3}"` |
- uk.ac.ed.inf.mulanxml.ec.EcTable

  | Modifier and Type | Constant Field | Value |
  |  |  |  |
  | --- | --- | --- |
  | `public static final java.lang.String` | `EC_TABLE_NAME` | `"ec_ancestors"` |

- uk.ac.ed.inf.mulanxml.test.LocalDbReaderTest

  | Modifier and Type | Constant Field | Value |
  |  |  |  |
  | --- | --- | --- |
  | `public static final java.lang.String` | `GET_EC_QUERY_1` | `"SELECT distinct ec FROM ec_status where status = \'ok\' "` |
  | `public static final java.lang.String` | `GET_EC_QUERY_2` | `"SELECT distinct ancestor FROM ec_ancestors_no0_nodash"` |
  | `public static final java.lang.String` | `TEST_DB_CONN_PATH` | `"src/uk/ac/ed/inf/mulanxml/dbconnection_test.props"` |

- Overview
- Package
- Class
- Use
- Tree
- Deprecated
- Index
- Help

- Prev
- Next

- Frames
- No Frames

- All Classes
