# Supplementary material for: From sequence to enzyme mechanism using multi-label machine learning
Source: BMC Bioinformatics. 2014 May 19;15:150. doi: 10.1186/1471-2105-15-150 (PMC4229970; doi:10.1186/1471-2105-15-150)
Supplement: Additional file 2 — Java code of ml2db. Additional file ml2db_code.tar.gz contains the Java source code to run the multi-label machine learning experiments and save the results to database. The code’s Javadoc is included. [file 1471-2105-15-150-S2.zip › additional file 2/ml2db/ecmulan/doc/index-files/index-1.html]

A-Index


JavaScript is disabled on your browser.


- Overview
- Package
- Class
- Use
- Tree
- Deprecated
- Index
- Help

- Prev Letter
- Next Letter

- Frames
- No Frames

- All Classes

A C D E F G I L M S T U W X 


## A

addChildElement(MulanLabel) - Method in class uk.ac.ed.inf.mulanxml.MulanLabel
:   Add a child label to a label

addChildElement(String) - Method in class uk.ac.ed.inf.mulanxml.MulanLabel
:   Add a child label to a label

AllTests - Class in uk.ac.ed.inf.mulanxml.test
:   Tests for Enzyme Commission number utility code.

AllTests() - Constructor for class uk.ac.ed.inf.mulanxml.test.AllTests


ANCESTOR\_FIELD\_NAME - Static variable in class uk.ac.ed.inf.mulanxml.ec.EcDbWriter
:   the name of the field to contain the ec number's ancestors

A C D E F G I L M S T U W X

- Overview
- Package
- Class
- Use
- Tree
- Deprecated
- Index
- Help

- Prev Letter
- Next Letter

- Frames
- No Frames

- All Classes
