# Supplementary material for: From sequence to enzyme mechanism using multi-label machine learning
Source: BMC Bioinformatics. 2014 May 19;15:150. doi: 10.1186/1471-2105-15-150 (PMC4229970; doi:10.1186/1471-2105-15-150)
Supplement: Additional file 2 — Java code of ml2db. Additional file ml2db_code.tar.gz contains the Java source code to run the multi-label machine learning experiments and save the results to database. The code’s Javadoc is included. [file 1471-2105-15-150-S2.zip › additional file 2/ml2db/ecmulan/doc/index-files/index-10.html]

S-Index


JavaScript is disabled on your browser.


- Overview
- Package
- Class
- Use
- Tree
- Deprecated
- Index
- Help

- Prev Letter
- Next Letter

- Frames
- No Frames

- All Classes

A C D E F G I L M S T U W X 


## S

saveRow(String, String) - Method in class uk.ac.ed.inf.mulanxml.ec.EcTable
:   Write one ec number + ancestor couple to table

saveToFile(String) - Method in class uk.ac.ed.inf.mulanxml.XmlCreator


setUp() - Method in class uk.ac.ed.inf.mulanxml.test.ec.EcDbWriterTest


setUp() - Method in class uk.ac.ed.inf.mulanxml.test.ec.EcFullXmlCreatorTest


setUp() - Method in class uk.ac.ed.inf.mulanxml.test.ec.EcMulanXmlCreatorTest


setUp() - Method in class uk.ac.ed.inf.mulanxml.test.ec.EcNumberGeneratorTest


setUp() - Method in class uk.ac.ed.inf.mulanxml.test.MulanLabelTest


setUp() - Method in class uk.ac.ed.inf.mulanxml.test.MulanXmlTest


STATUS\_FIELD - Static variable in class uk.ac.ed.inf.mulanxml.test.LocalDbReaderTest


suite() - Static method in class uk.ac.ed.inf.mulanxml.test.AllTests

A C D E F G I L M S T U W X

- Overview
- Package
- Class
- Use
- Tree
- Deprecated
- Index
- Help

- Prev Letter
- Next Letter

- Frames
- No Frames

- All Classes
