# Supplementary material for: From sequence to enzyme mechanism using multi-label machine learning
Source: BMC Bioinformatics. 2014 May 19;15:150. doi: 10.1186/1471-2105-15-150 (PMC4229970; doi:10.1186/1471-2105-15-150)
Supplement: Additional file 2 — Java code of ml2db. Additional file ml2db_code.tar.gz contains the Java source code to run the multi-label machine learning experiments and save the results to database. The code’s Javadoc is included. [file 1471-2105-15-150-S2.zip › additional file 2/ml2db/ecmulan/doc/index-files/index-11.html]

T-Index


JavaScript is disabled on your browser.


- Overview
- Package
- Class
- Use
- Tree
- Deprecated
- Index
- Help

- Prev Letter
- Next Letter

- Frames
- No Frames

- All Classes

A C D E F G I L M S T U W X 


## T

tearDown() - Method in class uk.ac.ed.inf.mulanxml.test.LocalDbReaderTest


TEST\_DB\_CONN\_PATH - Static variable in class uk.ac.ed.inf.mulanxml.test.LocalDbReaderTest
:   the test database

testAddChildToNode() - Method in class uk.ac.ed.inf.mulanxml.test.MulanLabelTest


testCompareTo() - Method in class uk.ac.ed.inf.mulanxml.test.ec.EcNumberTest


testCouldBeDashedEc() - Method in class uk.ac.ed.inf.mulanxml.test.ec.EcNumberGeneratorTest


testCouldBeEc() - Method in class uk.ac.ed.inf.mulanxml.test.ec.EcNumberGeneratorTest


testCreateEcTable() - Method in class uk.ac.ed.inf.mulanxml.test.ec.EcDbWriterTest


testData() - Method in class uk.ac.ed.inf.mulanxml.test.ec.EcDbWriterTest


testEcFullXmlCreator() - Method in class uk.ac.ed.inf.mulanxml.test.ec.EcFullXmlCreatorTest


testEcMulanXmlCreator() - Method in class uk.ac.ed.inf.mulanxml.test.ec.EcMulanXmlCreatorTest


testGetBlock1() - Method in class uk.ac.ed.inf.mulanxml.test.ec.EcNumberTest


testGetBlock2() - Method in class uk.ac.ed.inf.mulanxml.test.ec.EcNumberTest


testGetBlock3() - Method in class uk.ac.ed.inf.mulanxml.test.ec.EcNumberTest


testGetBlock4() - Method in class uk.ac.ed.inf.mulanxml.test.ec.EcNumberTest


testGetEcList() - Method in class uk.ac.ed.inf.mulanxml.test.LocalDbReaderTest


testGetHierarchy() - Method in class uk.ac.ed.inf.mulanxml.test.ec.EcNumberTest


testGetHierarchyLevel() - Method in class uk.ac.ed.inf.mulanxml.test.ec.EcNumberTest


testGetLabels() - Method in class uk.ac.ed.inf.mulanxml.XmlCreatorManagerTest
:   Test method for `XmlCreatorManager.getLabels()`.

testGetParent() - Method in class uk.ac.ed.inf.mulanxml.test.ec.EcNumberTest


testGetRoot() - Method in class uk.ac.ed.inf.mulanxml.test.MulanXmlTest


testGetXmlString() - Method in class uk.ac.ed.inf.mulanxml.XmlCreatorTest
:   Test method for `XmlCreator.getXml()`.

testIsComplete() - Method in class uk.ac.ed.inf.mulanxml.test.ec.EcNumberTest


testIsParent() - Method in class uk.ac.ed.inf.mulanxml.test.ec.EcNumberTest


testLabelNode() - Method in class uk.ac.ed.inf.mulanxml.test.MulanLabelTest


testLog() - Method in class uk.ac.ed.inf.mulanxml.test.ec.EcFullXmlCreatorTest


testSplitString() - Method in class uk.ac.ed.inf.mulanxml.test.ec.EcNumberTest


testValidateBlock() - Method in class uk.ac.ed.inf.mulanxml.test.ec.EcNumberGeneratorTest


toString() - Method in class uk.ac.ed.inf.mulanxml.MulanXml
:   public MulanLabel removeNode(String label) { // find node MulanLabel node
    = this.findNode(label); // get parent MulanLabel parent = node.

A C D E F G I L M S T U W X

- Overview
- Package
- Class
- Use
- Tree
- Deprecated
- Index
- Help

- Prev Letter
- Next Letter

- Frames
- No Frames

- All Classes
