# Supplementary material for: From sequence to enzyme mechanism using multi-label machine learning
Source: BMC Bioinformatics. 2014 May 19;15:150. doi: 10.1186/1471-2105-15-150 (PMC4229970; doi:10.1186/1471-2105-15-150)
Supplement: Additional file 2 — Java code of ml2db. Additional file ml2db_code.tar.gz contains the Java source code to run the multi-label machine learning experiments and save the results to database. The code’s Javadoc is included. [file 1471-2105-15-150-S2.zip › additional file 2/ml2db/ecmulan/doc/index-files/index-14.html]

X-Index


JavaScript is disabled on your browser.


- Overview
- Package
- Class
- Use
- Tree
- Deprecated
- Index
- Help

- Prev Letter
- Next Letter

- Frames
- No Frames

- All Classes

A C D E F G I L M S T U W X 


## X

XmlCreator - Class in uk.ac.ed.inf.mulanxml
:   Class

XmlCreator(XmlCreatorManager, TreeSet<String>) - Constructor for class uk.ac.ed.inf.mulanxml.XmlCreator
:   Get labels from database

XmlCreatorManager - Class in uk.ac.ed.inf.mulanxml
:   Checks the nature of the data: if the labels are EC numbers, it generates an
    ECNumber xml creator, if not, it generates a plain xml creator.

XmlCreatorManager(String, String) - Constructor for class uk.ac.ed.inf.mulanxml.XmlCreatorManager


XmlCreatorManagerTest - Class in uk.ac.ed.inf.mulanxml
:   Class

XmlCreatorManagerTest() - Constructor for class uk.ac.ed.inf.mulanxml.XmlCreatorManagerTest


XmlCreatorTest - Class in uk.ac.ed.inf.mulanxml
:   Class

XmlCreatorTest() - Constructor for class uk.ac.ed.inf.mulanxml.XmlCreatorTest

A C D E F G I L M S T U W X

- Overview
- Package
- Class
- Use
- Tree
- Deprecated
- Index
- Help

- Prev Letter
- Next Letter

- Frames
- No Frames

- All Classes
