# Supplementary material for: From sequence to enzyme mechanism using multi-label machine learning
Source: BMC Bioinformatics. 2014 May 19;15:150. doi: 10.1186/1471-2105-15-150 (PMC4229970; doi:10.1186/1471-2105-15-150)
Supplement: Additional file 2 — Java code of ml2db. Additional file ml2db_code.tar.gz contains the Java source code to run the multi-label machine learning experiments and save the results to database. The code’s Javadoc is included. [file 1471-2105-15-150-S2.zip › additional file 2/ml2db/ecmulan/doc/index-files/index-2.html]

C-Index


JavaScript is disabled on your browser.


- Overview
- Package
- Class
- Use
- Tree
- Deprecated
- Index
- Help

- Prev Letter
- Next Letter

- Frames
- No Frames

- All Classes

A C D E F G I L M S T U W X 


## C

compareTo(EcNumber) - Method in class uk.ac.ed.inf.mulanxml.ec.EcNumber
:   Order by EC string

couldBeDashedEc(String) - Static method in class uk.ac.ed.inf.mulanxml.ec.EcNumberGenerator
:   Checks whether the string could contain a validly formatted ec number
    with dashes (1.-.-.-, 1.2.3.- etc., but not 1.-.3.4)

couldBeEc(String) - Static method in class uk.ac.ed.inf.mulanxml.ec.EcNumberGenerator
:   Checks if the string could contain a validly formatted EC number.

createTestTable() - Method in class uk.ac.ed.inf.mulanxml.test.LocalDbReaderTest
:   Create a test EC table in the test database

createXmlString() - Method in class uk.ac.ed.inf.mulanxml.ec.EcFullXmlCreator
:   Generate an XML representation of the Enzyme Commission number hierarchy
    in Mulan format.

createXmlString() - Method in class uk.ac.ed.inf.mulanxml.ec.EcMulanXmlCreator


createXmlString() - Method in class uk.ac.ed.inf.mulanxml.XmlCreator
:   Generate a flat XML representation of the labels in Mulan format.

A C D E F G I L M S T U W X

- Overview
- Package
- Class
- Use
- Tree
- Deprecated
- Index
- Help

- Prev Letter
- Next Letter

- Frames
- No Frames

- All Classes
