# Supplementary material for: From sequence to enzyme mechanism using multi-label machine learning
Source: BMC Bioinformatics. 2014 May 19;15:150. doi: 10.1186/1471-2105-15-150 (PMC4229970; doi:10.1186/1471-2105-15-150)
Supplement: Additional file 2 — Java code of ml2db. Additional file ml2db_code.tar.gz contains the Java source code to run the multi-label machine learning experiments and save the results to database. The code’s Javadoc is included. [file 1471-2105-15-150-S2.zip › additional file 2/ml2db/ecmulan/doc/index-files/index-4.html]

E-Index


JavaScript is disabled on your browser.


- Overview
- Package
- Class
- Use
- Tree
- Deprecated
- Index
- Help

- Prev Letter
- Next Letter

- Frames
- No Frames

- All Classes

A C D E F G I L M S T U W X 


## E

EC\_DATA\_TYPE - Static variable in class uk.ac.ed.inf.mulanxml.ec.EcDbWriter
:   the sql data type for ec numbers

EC\_FIELD - Static variable in class uk.ac.ed.inf.mulanxml.test.LocalDbReaderTest


EC\_FIELD\_NAME - Static variable in class uk.ac.ed.inf.mulanxml.ec.EcDbWriter
:   the name of the field to contain the ec number

EC\_TABLE\_NAME - Static variable in class uk.ac.ed.inf.mulanxml.ec.EcTable
:   the name of the table for ec and ancestors

EcDbWriter - Class in uk.ac.ed.inf.mulanxml.ec
:   Given a database and a list of Enzyme commission numbers, writes a 2 columns
    table containing: in column 1: the Ec number, in column 2: all the ancestors
    of that EC number, including itself.

EcDbWriter(DbManager, String) - Constructor for class uk.ac.ed.inf.mulanxml.ec.EcDbWriter
:   Reads ec numbers from database and writes them and their ancestors to a
    new table

EcDbWriterTest - Class in uk.ac.ed.inf.mulanxml.test.ec
:   Class to test ecdbwriter and ectable

EcDbWriterTest() - Constructor for class uk.ac.ed.inf.mulanxml.test.ec.EcDbWriterTest


EcFullXmlCreator - Class in uk.ac.ed.inf.mulanxml.ec
:   Creates a full XML hierarchical representation of Enzyme Commission numbers
    in Mulan format.

EcFullXmlCreator(XmlCreatorManager, TreeSet<String>) - Constructor for class uk.ac.ed.inf.mulanxml.ec.EcFullXmlCreator
:   Get EC numbers from database

EcFullXmlCreatorTest - Class in uk.ac.ed.inf.mulanxml.test.ec
:   Class

EcFullXmlCreatorTest() - Constructor for class uk.ac.ed.inf.mulanxml.test.ec.EcFullXmlCreatorTest


EcMulanXmlCreator - Class in uk.ac.ed.inf.mulanxml.ec
:   Creates a full XML hierarchical representation of Enzyme Commission numbers
    in Mulan format.

EcMulanXmlCreator(XmlCreatorManager, TreeSet<String>) - Constructor for class uk.ac.ed.inf.mulanxml.ec.EcMulanXmlCreator


EcMulanXmlCreatorTest - Class in uk.ac.ed.inf.mulanxml.test.ec
:   Class

EcMulanXmlCreatorTest() - Constructor for class uk.ac.ed.inf.mulanxml.test.ec.EcMulanXmlCreatorTest


EcNumber - Class in uk.ac.ed.inf.mulanxml.ec
:   A class representing an Enzyme Commission (EC) number.

EcNumber(String) - Constructor for class uk.ac.ed.inf.mulanxml.ec.EcNumber


EcNumberGenerator - Class in uk.ac.ed.inf.mulanxml.ec
:   Given a string such as 1.2.3.4 or 1.2.-.-.- checks if it is a valid Enzyme
    commission number and generates the Java EcNumber object (including its
    parent EC numbers)

EcNumberGenerator(String) - Constructor for class uk.ac.ed.inf.mulanxml.ec.EcNumberGenerator


EcNumberGeneratorTest - Class in uk.ac.ed.inf.mulanxml.test.ec
:   Class

EcNumberGeneratorTest() - Constructor for class uk.ac.ed.inf.mulanxml.test.ec.EcNumberGeneratorTest


EcNumberTest - Class in uk.ac.ed.inf.mulanxml.test.ec
:   Class

EcNumberTest() - Constructor for class uk.ac.ed.inf.mulanxml.test.ec.EcNumberTest


EcTable - Class in uk.ac.ed.inf.mulanxml.ec
:   The table to store Enzyme commission numbers and their ancestors.

EcTable(DbManager) - Constructor for class uk.ac.ed.inf.mulanxml.ec.EcTable

A C D E F G I L M S T U W X

- Overview
- Package
- Class
- Use
- Tree
- Deprecated
- Index
- Help

- Prev Letter
- Next Letter

- Frames
- No Frames

- All Classes
