# Supplementary material for: From sequence to enzyme mechanism using multi-label machine learning
Source: BMC Bioinformatics. 2014 May 19;15:150. doi: 10.1186/1471-2105-15-150 (PMC4229970; doi:10.1186/1471-2105-15-150)
Supplement: Additional file 2 — Java code of ml2db. Additional file ml2db_code.tar.gz contains the Java source code to run the multi-label machine learning experiments and save the results to database. The code’s Javadoc is included. [file 1471-2105-15-150-S2.zip › additional file 2/ml2db/ecmulan/doc/index-files/index-6.html]

G-Index


JavaScript is disabled on your browser.


- Overview
- Package
- Class
- Use
- Tree
- Deprecated
- Index
- Help

- Prev Letter
- Next Letter

- Frames
- No Frames

- All Classes

A C D E F G I L M S T U W X 


## G

generateEcNumber(String) - Static method in class uk.ac.ed.inf.mulanxml.ec.EcNumberGenerator


get0dashEcA() - Static method in class uk.ac.ed.inf.mulanxml.test.ec.EcNumberTest


get0dashEcB() - Static method in class uk.ac.ed.inf.mulanxml.test.ec.EcNumberTest


get1dashEc() - Static method in class uk.ac.ed.inf.mulanxml.test.ec.EcNumberTest


get2dashEc() - Static method in class uk.ac.ed.inf.mulanxml.test.ec.EcNumberTest


get3dashEc() - Static method in class uk.ac.ed.inf.mulanxml.test.ec.EcNumberTest


get4dashEc() - Static method in class uk.ac.ed.inf.mulanxml.test.ec.EcNumberTest


GET\_EC\_QUERY\_1 - Static variable in class uk.ac.ed.inf.mulanxml.test.LocalDbReaderTest


GET\_EC\_QUERY\_2 - Static variable in class uk.ac.ed.inf.mulanxml.test.LocalDbReaderTest


getAncestorsStrings() - Method in class uk.ac.ed.inf.mulanxml.ec.EcNumber
:   Get a complete hierarchy of ancestor for the ec number.

getBlocks() - Method in class uk.ac.ed.inf.mulanxml.ec.EcNumber


getColumnDefinitions() - Static method in class uk.ac.ed.inf.mulanxml.ec.EcTable
:   Creates the table for the ec numbers and ancestors

getDbReader() - Method in class uk.ac.ed.inf.mulanxml.XmlCreatorManager


getEcDbWriter() - Static method in class uk.ac.ed.inf.mulanxml.test.ec.EcDbWriterTest


getEcNumbers() - Method in class uk.ac.ed.inf.mulanxml.ec.EcFullXmlCreator


getEcSqlQuery() - Method in class uk.ac.ed.inf.mulanxml.LocalDbReader


getEcString() - Method in class uk.ac.ed.inf.mulanxml.ec.EcNumber


getEcStringFromBlocks(String[]) - Static method in class uk.ac.ed.inf.mulanxml.ec.EcNumber


getEcTable() - Method in class uk.ac.ed.inf.mulanxml.ec.EcDbWriter


getHierarchyLevel() - Method in class uk.ac.ed.inf.mulanxml.ec.EcNumber


getLabelList() - Method in class uk.ac.ed.inf.mulanxml.LocalDbReader


getLabels() - Method in class uk.ac.ed.inf.mulanxml.XmlCreator


getLabels() - Method in class uk.ac.ed.inf.mulanxml.XmlCreatorManager


getManager() - Method in class uk.ac.ed.inf.mulanxml.XmlCreator


getMulanLabel() - Static method in class uk.ac.ed.inf.mulanxml.test.MulanLabelTest


getMulanLabelXml() - Static method in class uk.ac.ed.inf.mulanxml.test.MulanXmlTest


getParent() - Method in class uk.ac.ed.inf.mulanxml.ec.EcNumber


getParentString() - Method in class uk.ac.ed.inf.mulanxml.ec.EcNumber


getRoot() - Method in class uk.ac.ed.inf.mulanxml.MulanXml


getSet() - Static method in class uk.ac.ed.inf.mulanxml.test.ec.EcFullXmlCreatorTest


getXml() - Method in class uk.ac.ed.inf.mulanxml.XmlCreator


getXmlCreator() - Method in class uk.ac.ed.inf.mulanxml.XmlCreatorManager


getXmlCreator() - Static method in class uk.ac.ed.inf.mulanxml.XmlCreatorTest
:   Test method for `XmlCreator.XmlCreator(uk.ac.ed.inf.mulanxml.XmlCreatorManager, java.util.TreeSet)`.

getXmlCreator0dashA() - Static method in class uk.ac.ed.inf.mulanxml.test.ec.EcFullXmlCreatorTest


getXmlCreator1dash() - Static method in class uk.ac.ed.inf.mulanxml.test.ec.EcFullXmlCreatorTest


getXmlCreator2dash() - Static method in class uk.ac.ed.inf.mulanxml.test.ec.EcFullXmlCreatorTest


getXmlCreator3dash() - Static method in class uk.ac.ed.inf.mulanxml.test.ec.EcFullXmlCreatorTest


getXmlCreator3EcNumbers() - Static method in class uk.ac.ed.inf.mulanxml.test.ec.EcFullXmlCreatorTest


getXmlCreator4dash() - Static method in class uk.ac.ed.inf.mulanxml.test.ec.EcFullXmlCreatorTest


getXmlCreator5() - Static method in class uk.ac.ed.inf.mulanxml.test.ec.EcMulanXmlCreatorTest


getXmlCreatorManager1() - Static method in class uk.ac.ed.inf.mulanxml.XmlCreatorManagerTest
:   Test method for `XmlCreatorManager.XmlCreatorManager(java.lang.String, java.lang.String)`.

getXmlCreatorWithDbConn() - Static method in class uk.ac.ed.inf.mulanxml.test.ec.EcFullXmlCreatorTest


getXmlString() - Method in class uk.ac.ed.inf.mulanxml.XmlCreator
:   Adds a log to the xml file (timestamp, database and query to the data
    etc.)

A C D E F G I L M S T U W X

- Overview
- Package
- Class
- Use
- Tree
- Deprecated
- Index
- Help

- Prev Letter
- Next Letter

- Frames
- No Frames

- All Classes
