# Supplementary material for: From sequence to enzyme mechanism using multi-label machine learning
Source: BMC Bioinformatics. 2014 May 19;15:150. doi: 10.1186/1471-2105-15-150 (PMC4229970; doi:10.1186/1471-2105-15-150)
Supplement: Additional file 2 — Java code of ml2db. Additional file ml2db_code.tar.gz contains the Java source code to run the multi-label machine learning experiments and save the results to database. The code’s Javadoc is included. [file 1471-2105-15-150-S2.zip › additional file 2/ml2db/ecmulan/doc/index-files/index-7.html]

I-Index


JavaScript is disabled on your browser.


- Overview
- Package
- Class
- Use
- Tree
- Deprecated
- Index
- Help

- Prev Letter
- Next Letter

- Frames
- No Frames

- All Classes

A C D E F G I L M S T U W X 


## I

isComplete() - Method in class uk.ac.ed.inf.mulanxml.ec.EcNumber


isParent(EcNumber) - Method in class uk.ac.ed.inf.mulanxml.ec.EcNumber
:   Returns true if the node given is a direct child of this node: e.g:
    1.2.3.4 is direct child of 1.2.3.-, but it is not direct child of 1.2.-.-

isValidEcBlock(String) - Static method in class uk.ac.ed.inf.mulanxml.ec.EcNumberGenerator
:   True if the string contains a dash '-' or a positive integer or a
    positive integer preceded by 'n' (new uniprot ec numbers)

A C D E F G I L M S T U W X

- Overview
- Package
- Class
- Use
- Tree
- Deprecated
- Index
- Help

- Prev Letter
- Next Letter

- Frames
- No Frames

- All Classes
