# Supplementary material for: From sequence to enzyme mechanism using multi-label machine learning
Source: BMC Bioinformatics. 2014 May 19;15:150. doi: 10.1186/1471-2105-15-150 (PMC4229970; doi:10.1186/1471-2105-15-150)
Supplement: Additional file 2 — Java code of ml2db. Additional file ml2db_code.tar.gz contains the Java source code to run the multi-label machine learning experiments and save the results to database. The code’s Javadoc is included. [file 1471-2105-15-150-S2.zip › additional file 2/ml2db/ecmulan/doc/index-files/index-9.html]

M-Index


JavaScript is disabled on your browser.


- Overview
- Package
- Class
- Use
- Tree
- Deprecated
- Index
- Help

- Prev Letter
- Next Letter

- Frames
- No Frames

- All Classes

A C D E F G I L M S T U W X 


## M

m\_ecSqlQuery - Variable in class uk.ac.ed.inf.mulanxml.LocalDbReader


m\_root - Variable in class uk.ac.ed.inf.mulanxml.MulanXml
:   the xml tree root

main(String[]) - Static method in class uk.ac.ed.inf.mulanxml.ec.EcDbWriter
:   Main for recreating table

main(String[]) - Static method in class uk.ac.ed.inf.mulanxml.ec.EcFullXmlCreator


main(String[]) - Static method in class uk.ac.ed.inf.mulanxml.ec.EcMulanXmlCreator


main(String[]) - Static method in class uk.ac.ed.inf.mulanxml.test.AllTests


MAX\_LEVEL1\_CLASS - Static variable in class uk.ac.ed.inf.mulanxml.ec.EcNumberGenerator
:   maximum value for level 1 class: currently 6.

MAX\_LEVEL1\_REGEXP - Static variable in class uk.ac.ed.inf.mulanxml.ec.EcNumberGenerator
:   regexp from 1 to 6

MAX\_LEVEL2\_CLASS - Static variable in class uk.ac.ed.inf.mulanxml.ec.EcNumberGenerator
:   maximum value for level 2 class: 99 in this implementation.

MAX\_LEVEL2\_REGEXP - Static variable in class uk.ac.ed.inf.mulanxml.ec.EcNumberGenerator
:   regexp from 0 to 99

MAX\_LEVEL3\_CLASS - Static variable in class uk.ac.ed.inf.mulanxml.ec.EcNumberGenerator
:   maximum value for level 3 class: 99 in this implementation.

MAX\_LEVEL3\_REGEXP - Static variable in class uk.ac.ed.inf.mulanxml.ec.EcNumberGenerator
:   regexp from 0 to 99

MAX\_LEVEL4\_CLASS - Static variable in class uk.ac.ed.inf.mulanxml.ec.EcNumberGenerator
:   maximum value for level 4 class: 999 in this implementation.

MAX\_LEVEL4\_REGEXP - Static variable in class uk.ac.ed.inf.mulanxml.ec.EcNumberGenerator
:   regexp from 0 to 999, potentially prefixed with "n" in uniprot ("new" ec numbers)

MULAN\_XML\_ROOT\_TAG - Static variable in class uk.ac.ed.inf.mulanxml.MulanXml


MULAN\_XML\_ROOT\_TAG\_ATTRIBUTE\_NAME - Static variable in class uk.ac.ed.inf.mulanxml.MulanXml


MULAN\_XML\_ROOT\_TAG\_ATTRIBUTE\_VALUE - Static variable in class uk.ac.ed.inf.mulanxml.MulanXml


MulanLabel - Class in uk.ac.ed.inf.mulanxml
:   A node in the Mulan XML (a label for machine learning) \*

MulanLabel(String) - Constructor for class uk.ac.ed.inf.mulanxml.MulanLabel


MulanLabelTest - Class in uk.ac.ed.inf.mulanxml.test
:   Class

MulanLabelTest() - Constructor for class uk.ac.ed.inf.mulanxml.test.MulanLabelTest


MulanXml - Class in uk.ac.ed.inf.mulanxml
:   Generates an XML file for labels in the Mulan format
    http://mulan.sourceforge.net/ http://mlkd.csd.auth.gr/multilabel.html

MulanXml() - Constructor for class uk.ac.ed.inf.mulanxml.MulanXml


MulanXmlTest - Class in uk.ac.ed.inf.mulanxml.test
:   Test Generates an XML file for labels in the Mulan format
    http://mulan.sourceforge.net/ http://mlkd.csd.auth.gr/multilabel.html

MulanXmlTest() - Constructor for class uk.ac.ed.inf.mulanxml.test.MulanXmlTest

A C D E F G I L M S T U W X

- Overview
- Package
- Class
- Use
- Tree
- Deprecated
- Index
- Help

- Prev Letter
- Next Letter

- Frames
- No Frames

- All Classes
