# Supplementary material for: From sequence to enzyme mechanism using multi-label machine learning
Source: BMC Bioinformatics. 2014 May 19;15:150. doi: 10.1186/1471-2105-15-150 (PMC4229970; doi:10.1186/1471-2105-15-150)
Supplement: Additional file 2 — Java code of ml2db. Additional file ml2db_code.tar.gz contains the Java source code to run the multi-label machine learning experiments and save the results to database. The code’s Javadoc is included. [file 1471-2105-15-150-S2.zip › additional file 2/ml2db/ecmulan/doc/overview-tree.html]

Class Hierarchy


JavaScript is disabled on your browser.


- Overview
- Package
- Class
- Use
- Tree
- Deprecated
- Index
- Help

- Prev
- Next

- Frames
- No Frames

- All Classes

# Hierarchy For All Packages

Package Hierarchies:

- uk.ac.ed.inf.mulanxml,
- uk.ac.ed.inf.mulanxml.ec,
- uk.ac.ed.inf.mulanxml.test,
- uk.ac.ed.inf.mulanxml.test.ec

## Class Hierarchy

- java.lang.Object
  - uk.ac.ed.inf.mulanxml.test.AllTests
  - junit.framework.Assert
    - junit.framework.TestCase (implements junit.framework.Test)
      - uk.ac.ed.inf.mulanxml.test.ec.EcDbWriterTest
      - uk.ac.ed.inf.mulanxml.test.ec.EcFullXmlCreatorTest
      - uk.ac.ed.inf.mulanxml.test.ec.EcMulanXmlCreatorTest
      - uk.ac.ed.inf.mulanxml.test.ec.EcNumberGeneratorTest
      - uk.ac.ed.inf.mulanxml.test.ec.EcNumberTest
      - uk.ac.ed.inf.mulanxml.test.LocalDbReaderTest
      - uk.ac.ed.inf.mulanxml.test.MulanLabelTest
      - uk.ac.ed.inf.mulanxml.test.MulanXmlTest
      - uk.ac.ed.inf.mulanxml.XmlCreatorManagerTest
      - uk.ac.ed.inf.mulanxml.XmlCreatorTest
  - uk.ac.standrews.utils.main.database.DbManaged
    - uk.ac.standrews.utils.main.database.DbReader
      - uk.ac.ed.inf.mulanxml.LocalDbReader
  - uk.ac.ed.inf.mulanxml.ec.EcNumber (implements java.lang.Comparable<T>)
  - uk.ac.ed.inf.mulanxml.ec.EcNumberGenerator
  - uk.ac.standrews.utils.main.database.Manager
    - uk.ac.standrews.utils.main.database.DbManager (implements java.io.Serializable)
      - uk.ac.ed.inf.mulanxml.ec.EcDbWriter
  - uk.ac.ed.inf.mulanxml.MulanXml
  - uk.ac.standrews.utils.main.database.Table
    - uk.ac.ed.inf.mulanxml.ec.EcTable
  - uk.ac.ed.inf.mulanxml.XmlCreator
    - uk.ac.ed.inf.mulanxml.ec.EcFullXmlCreator
      - uk.ac.ed.inf.mulanxml.ec.EcMulanXmlCreator
  - uk.ac.ed.inf.mulanxml.XmlCreatorManager
  - uk.ac.standrews.utils.main.webutils.simpledomparser.XmlNode
    - uk.ac.ed.inf.mulanxml.MulanLabel

- Overview
- Package
- Class
- Use
- Tree
- Deprecated
- Index
- Help

- Prev
- Next

- Frames
- No Frames

- All Classes
