# Supplementary material for: From sequence to enzyme mechanism using multi-label machine learning
Source: BMC Bioinformatics. 2014 May 19;15:150. doi: 10.1186/1471-2105-15-150 (PMC4229970; doi:10.1186/1471-2105-15-150)
Supplement: Additional file 2 — Java code of ml2db. Additional file ml2db_code.tar.gz contains the Java source code to run the multi-label machine learning experiments and save the results to database. The code’s Javadoc is included. [file 1471-2105-15-150-S2.zip › additional file 2/ml2db/ecmulan/doc/serialized-form.html]

Serialized Form


JavaScript is disabled on your browser.


- Overview
- Package
- Class
- Use
- Tree
- Deprecated
- Index
- Help

- Prev
- Next

- Frames
- No Frames

- All Classes

# Serialized Form

- ## Package uk.ac.ed.inf.mulanxml.ec

  - ### Class uk.ac.ed.inf.mulanxml.ec.EcDbWriter extends uk.ac.standrews.utils.main.database.DbManager implements Serializable

    serialVersionUID:
    :   786521491699853235L

    - ### Serialized Fields

      - #### m\_debug

        ```
        boolean m_debug
        ```

        Debug: set to true when debugging (prints stuff to screen)
      - #### m\_ecNumbers

        ```
        java.util.Stack<E> m_ecNumbers
        ```

        the ec numbers. The list should be updated to include subclasses too.
        That is, the couples ([column1 value, column2 value]) such as [1234,
        1234][1234, 123-][1234, 12--][1234, 1---] should be written. But also the
        couples [123-, 123-][123-, 12--][123-, 1---] and [12--, 12--][12--, 1---]
        and [1---, 1---]
      - #### m\_ecTable

        ```
        EcTable m_ecTable
        ```

- Overview
- Package
- Class
- Use
- Tree
- Deprecated
- Index
- Help

- Prev
- Next

- Frames
- No Frames

- All Classes
