# Supplementary material for: From sequence to enzyme mechanism using multi-label machine learning
Source: BMC Bioinformatics. 2014 May 19;15:150. doi: 10.1186/1471-2105-15-150 (PMC4229970; doi:10.1186/1471-2105-15-150)
Supplement: Additional file 2 — Java code of ml2db. Additional file ml2db_code.tar.gz contains the Java source code to run the multi-label machine learning experiments and save the results to database. The code’s Javadoc is included. [file 1471-2105-15-150-S2.zip › additional file 2/ml2db/ecmulan/doc/uk/ac/ed/inf/ec/class-use/EcFullXmlCreator.html]

Uses of Class uk.ac.ed.inf.ec.EcFullXmlCreator


---


|  |  |  |  |  |  |  |  |  |  |  |
| --- | --- | --- | --- | --- | --- | --- | --- | --- | --- | --- |
| |  |  |  |  |  |  |  |  | | --- | --- | --- | --- | --- | --- | --- | --- | | **Overview** | **Package** | **Class** | **Use** | **Tree** | **Deprecated** | **Index** | **Help** | | |  |
| PREV   NEXT | **FRAMES**    **NO FRAMES**     **All Classes** |


---


## **Uses of Class uk.ac.ed.inf.ec.EcFullXmlCreator**

| Packages that use EcFullXmlCreator | |
| --- | --- |
| **uk.ac.ed.inf.ec** |  |
| **uk.ac.ed.inf.ec.test** |  |

| Uses of EcFullXmlCreator in uk.ac.ed.inf.ec | |
| --- | --- |

| Subclasses of EcFullXmlCreator in uk.ac.ed.inf.ec | |
| --- | --- |
| `class` | `EcMulanXmlCreator`             Creates a full XML hierarchical representation of Enzyme Commission numbers in Mulan format. |

| Uses of EcFullXmlCreator in uk.ac.ed.inf.ec.test | |
| --- | --- |

| Methods in uk.ac.ed.inf.ec.test that return EcFullXmlCreator | |
| --- | --- |
| `static EcFullXmlCreator` | `EcFullXmlCreatorTest.getXmlCreator0dashA()` |
| `static EcFullXmlCreator` | `EcFullXmlCreatorTest.getXmlCreator1dash()` |
| `static EcFullXmlCreator` | `EcFullXmlCreatorTest.getXmlCreator2dash()` |
| `static EcFullXmlCreator` | `EcFullXmlCreatorTest.getXmlCreator3dash()` |
| `static EcFullXmlCreator` | `EcFullXmlCreatorTest.getXmlCreator3EcNumbers()` |
| `static EcFullXmlCreator` | `EcFullXmlCreatorTest.getXmlCreator4dash()` |
| `static EcFullXmlCreator` | `EcFullXmlCreatorTest.getXmlCreatorWithDbConn()` |

---


|  |  |  |  |  |  |  |  |  |  |  |
| --- | --- | --- | --- | --- | --- | --- | --- | --- | --- | --- |
| |  |  |  |  |  |  |  |  | | --- | --- | --- | --- | --- | --- | --- | --- | | **Overview** | **Package** | **Class** | **Use** | **Tree** | **Deprecated** | **Index** | **Help** | | |  |
| PREV   NEXT | **FRAMES**    **NO FRAMES**     **All Classes** |


---
