# Supplementary material for: From sequence to enzyme mechanism using multi-label machine learning
Source: BMC Bioinformatics. 2014 May 19;15:150. doi: 10.1186/1471-2105-15-150 (PMC4229970; doi:10.1186/1471-2105-15-150)
Supplement: Additional file 2 — Java code of ml2db. Additional file ml2db_code.tar.gz contains the Java source code to run the multi-label machine learning experiments and save the results to database. The code’s Javadoc is included. [file 1471-2105-15-150-S2.zip › additional file 2/ml2db/ecmulan/doc/uk/ac/ed/inf/ec/class-use/EcNumber.html]

Uses of Class uk.ac.ed.inf.ec.EcNumber


---


|  |  |  |  |  |  |  |  |  |  |  |
| --- | --- | --- | --- | --- | --- | --- | --- | --- | --- | --- |
| |  |  |  |  |  |  |  |  | | --- | --- | --- | --- | --- | --- | --- | --- | | **Overview** | **Package** | **Class** | **Use** | **Tree** | **Deprecated** | **Index** | **Help** | | |  |
| PREV   NEXT | **FRAMES**    **NO FRAMES**     **All Classes** |


---


## **Uses of Class uk.ac.ed.inf.ec.EcNumber**

| Packages that use EcNumber | |
| --- | --- |
| **uk.ac.ed.inf.ec** |  |
| **uk.ac.ed.inf.ec.test** |  |

| Uses of EcNumber in uk.ac.ed.inf.ec | |
| --- | --- |

| Methods in uk.ac.ed.inf.ec that return EcNumber | |
| --- | --- |
| `static EcNumber` | `EcNumberGenerator.generateEcNumber(java.lang.String ec)` |
| `EcNumber` | `EcNumber.getParent()` |

| Methods in uk.ac.ed.inf.ec that return types with arguments of type EcNumber | |
| --- | --- |
| `java.util.TreeSet<EcNumber>` | `EcFullXmlCreator.getEcNumbers()` |

| Methods in uk.ac.ed.inf.ec with parameters of type EcNumber | |
| --- | --- |
| `int` | `EcNumber.compareTo(EcNumber otherEc)` |
| `boolean` | `EcNumber.isParent(EcNumber possibleChild)`             Returns true if the node given is a direct child of this node: e.g: 1.2.3.4 is direct child of 1.2.3.-, but it is not direct child of 1.2.-.- |

| Uses of EcNumber in uk.ac.ed.inf.ec.test | |
| --- | --- |

| Methods in uk.ac.ed.inf.ec.test that return EcNumber | |
| --- | --- |
| `static EcNumber` | `EcNumberTest.get0dashEcA()` |
| `static EcNumber` | `EcNumberTest.get0dashEcB()` |
| `static EcNumber` | `EcNumberTest.get1dashEc()` |
| `static EcNumber` | `EcNumberTest.get2dashEc()` |
| `static EcNumber` | `EcNumberTest.get3dashEc()` |
| `static EcNumber` | `EcNumberTest.get4dashEc()` |

---


|  |  |  |  |  |  |  |  |  |  |  |
| --- | --- | --- | --- | --- | --- | --- | --- | --- | --- | --- |
| |  |  |  |  |  |  |  |  | | --- | --- | --- | --- | --- | --- | --- | --- | | **Overview** | **Package** | **Class** | **Use** | **Tree** | **Deprecated** | **Index** | **Help** | | |  |
| PREV   NEXT | **FRAMES**    **NO FRAMES**     **All Classes** |


---
