# Supplementary material for: From sequence to enzyme mechanism using multi-label machine learning
Source: BMC Bioinformatics. 2014 May 19;15:150. doi: 10.1186/1471-2105-15-150 (PMC4229970; doi:10.1186/1471-2105-15-150)
Supplement: Additional file 2 — Java code of ml2db. Additional file ml2db_code.tar.gz contains the Java source code to run the multi-label machine learning experiments and save the results to database. The code’s Javadoc is included. [file 1471-2105-15-150-S2.zip › additional file 2/ml2db/ecmulan/doc/uk/ac/ed/inf/ec/class-use/MulanLabel.html]

Uses of Class uk.ac.ed.inf.ec.MulanLabel


---


|  |  |  |  |  |  |  |  |  |  |  |
| --- | --- | --- | --- | --- | --- | --- | --- | --- | --- | --- |
| |  |  |  |  |  |  |  |  | | --- | --- | --- | --- | --- | --- | --- | --- | | **Overview** | **Package** | **Class** | **Use** | **Tree** | **Deprecated** | **Index** | **Help** | | |  |
| PREV   NEXT | **FRAMES**    **NO FRAMES**     **All Classes** |


---


## **Uses of Class uk.ac.ed.inf.ec.MulanLabel**

| Packages that use MulanLabel | |
| --- | --- |
| **uk.ac.ed.inf.ec** |  |
| **uk.ac.ed.inf.ec.test** |  |

| Uses of MulanLabel in uk.ac.ed.inf.ec | |
| --- | --- |

| Fields in uk.ac.ed.inf.ec declared as MulanLabel | |
| --- | --- |
| `MulanLabel` | `MulanXml.m_root`             the xml tree root |

| Methods in uk.ac.ed.inf.ec that return MulanLabel | |
| --- | --- |
| `MulanLabel` | `MulanXml.findNode(java.lang.String label)`             Find a node by label |
| `MulanLabel` | `MulanXml.getRoot()` |

| Methods in uk.ac.ed.inf.ec with parameters of type MulanLabel | |
| --- | --- |
| `void` | `MulanLabel.addChildElement(MulanLabel label)`             Add a child label to a label |

| Uses of MulanLabel in uk.ac.ed.inf.ec.test | |
| --- | --- |

| Methods in uk.ac.ed.inf.ec.test that return MulanLabel | |
| --- | --- |
| `static MulanLabel` | `MulanLabelTest.getMulanLabel()` |

---


|  |  |  |  |  |  |  |  |  |  |  |
| --- | --- | --- | --- | --- | --- | --- | --- | --- | --- | --- |
| |  |  |  |  |  |  |  |  | | --- | --- | --- | --- | --- | --- | --- | --- | | **Overview** | **Package** | **Class** | **Use** | **Tree** | **Deprecated** | **Index** | **Help** | | |  |
| PREV   NEXT | **FRAMES**    **NO FRAMES**     **All Classes** |


---
