# Supplementary material for: From sequence to enzyme mechanism using multi-label machine learning
Source: BMC Bioinformatics. 2014 May 19;15:150. doi: 10.1186/1471-2105-15-150 (PMC4229970; doi:10.1186/1471-2105-15-150)
Supplement: Additional file 2 — Java code of ml2db. Additional file ml2db_code.tar.gz contains the Java source code to run the multi-label machine learning experiments and save the results to database. The code’s Javadoc is included. [file 1471-2105-15-150-S2.zip › additional file 2/ml2db/ecmulan/doc/uk/ac/ed/inf/ec/EcDbReader.html]

EcDbReader


---


|  |  |  |  |  |  |  |  |  |  |  |
| --- | --- | --- | --- | --- | --- | --- | --- | --- | --- | --- |
| |  |  |  |  |  |  |  |  | | --- | --- | --- | --- | --- | --- | --- | --- | | **Overview** | **Package** | **Class** | **Use** | **Tree** | **Deprecated** | **Index** | **Help** | | |  |
| PREV CLASS   **NEXT CLASS** | **FRAMES**    **NO FRAMES**     **All Classes** |
| SUMMARY: NESTED | FIELD | CONSTR | METHOD | DETAIL: FIELD | CONSTR | METHOD |


---


## uk.ac.ed.inf.ec Class EcDbReader

```
java.lang.Object
  uk.ac.ed.inf.utils.database.DbManaged
      uk.ac.ed.inf.utils.database.DbReader
          uk.ac.ed.inf.ec.EcDbReader
```

---

``` public class EcDbReader extends uk.ac.ed.inf.utils.database.DbReader ```

Reads the full list of Enzyme Commission numbers from database

**Version:**
:   5 May 2010

**Author:**
:   Luna De Ferrari luna.deferrari-at-ed.ac.uk

---

| **Field Summary** | |
| --- | --- |
| `java.lang.String` | `m_ecSqlQuery` |


| **Constructor Summary** | |
| --- | --- |
| `EcDbReader(uk.ac.ed.inf.utils.database.DbManager dbManager, java.lang.String ecSqlQuery)` |


| **Method Summary** | |
| --- | --- |
| `java.util.TreeSet<java.lang.String>` | `getEcList()` |
| `java.lang.String` | `getEcSqlQuery()` |

| **Methods inherited from class uk.ac.ed.inf.utils.database.DbReader** |
| --- |
| `executeQuery, getColumnValues, getDbMetaData, getListOfDbTables, getMap, getMap, getStatement, getTableReader, queryResultsColumnToCollection, queryResultsColumnToSet, queryResultsColumnToVector, tableExistsInDb` |

| **Methods inherited from class uk.ac.ed.inf.utils.database.DbManaged** |
| --- |
| `getDbConnection, getDbManager` |

| **Methods inherited from class java.lang.Object** |
| --- |
| `equals, getClass, hashCode, notify, notifyAll, toString, wait, wait, wait` |

| **Field Detail** |
| --- |

### m\_ecSqlQuery

```
public java.lang.String m_ecSqlQuery
```


| **Constructor Detail** |
| --- |

### EcDbReader

```
public EcDbReader(uk.ac.ed.inf.utils.database.DbManager dbManager,
                  java.lang.String ecSqlQuery)
```


| **Method Detail** |
| --- |

### getEcList

```
public java.util.TreeSet<java.lang.String> getEcList()
```

---


### getEcSqlQuery

```
public java.lang.String getEcSqlQuery()
```


---


|  |  |  |  |  |  |  |  |  |  |  |
| --- | --- | --- | --- | --- | --- | --- | --- | --- | --- | --- |
| |  |  |  |  |  |  |  |  | | --- | --- | --- | --- | --- | --- | --- | --- | | **Overview** | **Package** | **Class** | **Use** | **Tree** | **Deprecated** | **Index** | **Help** | | |  |
| PREV CLASS   **NEXT CLASS** | **FRAMES**    **NO FRAMES**     **All Classes** |
| SUMMARY: NESTED | FIELD | CONSTR | METHOD | DETAIL: FIELD | CONSTR | METHOD |


---
