# Supplementary material for: From sequence to enzyme mechanism using multi-label machine learning
Source: BMC Bioinformatics. 2014 May 19;15:150. doi: 10.1186/1471-2105-15-150 (PMC4229970; doi:10.1186/1471-2105-15-150)
Supplement: Additional file 2 — Java code of ml2db. Additional file ml2db_code.tar.gz contains the Java source code to run the multi-label machine learning experiments and save the results to database. The code’s Javadoc is included. [file 1471-2105-15-150-S2.zip › additional file 2/ml2db/ecmulan/doc/uk/ac/ed/inf/ec/EcDbWriter.html]

EcDbWriter


---


|  |  |  |  |  |  |  |  |  |  |  |
| --- | --- | --- | --- | --- | --- | --- | --- | --- | --- | --- |
| |  |  |  |  |  |  |  |  | | --- | --- | --- | --- | --- | --- | --- | --- | | **Overview** | **Package** | **Class** | **Use** | **Tree** | **Deprecated** | **Index** | **Help** | | |  |
| **PREV CLASS**   **NEXT CLASS** | **FRAMES**    **NO FRAMES**     **All Classes** |
| SUMMARY: NESTED | FIELD | CONSTR | METHOD | DETAIL: FIELD | CONSTR | METHOD |


---


## uk.ac.ed.inf.ec Class EcDbWriter

```
java.lang.Object
  uk.ac.ed.inf.utils.database.DbManaged
      uk.ac.ed.inf.ec.EcDbWriter
```

---

``` public class EcDbWriter extends uk.ac.ed.inf.utils.database.DbManaged ```

Given a database and a list of Enzyme commission numbers, writes a 2 columns
table containing: in column 1: the Ec number, in column 2: all the ancestors
of that EC number, including itself. (It also adds the EC number ancestors to
column 1, so that their subclasses are included in column 2 too)

**Version:**
:   3 Jun 2010

**Author:**
:   Luna De Ferrari luna.deferrari-at-ed.ac.uk

---

| **Field Summary** | |
| --- | --- |
| `static java.lang.String` | `ANCESTOR_FIELD_NAME`             the name of the field to contain the ec number's ancestors |
| `static java.lang.String` | `EC_DATA_TYPE`             the sql data type for ec numbers |
| `static java.lang.String` | `EC_FIELD_NAME`             the name of the field to contain the ec number |
| `static java.lang.String` | `EC_TABLE_NAME`             the name of the table for ec and ancestors |


| **Constructor Summary** | |
| --- | --- |
| `EcDbWriter(uk.ac.ed.inf.utils.database.DbManager manager, java.lang.String getEcSqlQuery)`             Reads ec numbers from database and writes them and their ancestors to a new table |


| **Method Summary** | |
| --- | --- |
| `uk.ac.ed.inf.utils.database.Table` | `getEcTable()`             Creates the table for the ec numbers and ancestors |
| `uk.ac.ed.inf.utils.database.TableManager` | `getTableManager()` |
| `static void` | `main(java.lang.String[] args)`             Main for recreating table |
| `void` | `writeEcAndAncestorsToTable()` |

| **Methods inherited from class uk.ac.ed.inf.utils.database.DbManaged** |
| --- |
| `getDbConnection, getDbManager` |

| **Methods inherited from class java.lang.Object** |
| --- |
| `equals, getClass, hashCode, notify, notifyAll, toString, wait, wait, wait` |

| **Field Detail** |
| --- |

### ANCESTOR\_FIELD\_NAME

```
public static final java.lang.String ANCESTOR_FIELD_NAME
```

:   the name of the field to contain the ec number's ancestors

    **See Also:**: Constant Field Values

---


### EC\_DATA\_TYPE

```
public static final java.lang.String EC_DATA_TYPE
```

:   the sql data type for ec numbers

    **See Also:**: Constant Field Values

---


### EC\_FIELD\_NAME

```
public static final java.lang.String EC_FIELD_NAME
```

:   the name of the field to contain the ec number

    **See Also:**: Constant Field Values

---


### EC\_TABLE\_NAME

```
public static final java.lang.String EC_TABLE_NAME
```

:   the name of the table for ec and ancestors

    **See Also:**: Constant Field Values


| **Constructor Detail** |
| --- |

### EcDbWriter

```
public EcDbWriter(uk.ac.ed.inf.utils.database.DbManager manager,
                  java.lang.String getEcSqlQuery)
```

:   Reads ec numbers from database and writes them and their ancestors to a
    new table


| **Method Detail** |
| --- |

### getEcTable

```
public uk.ac.ed.inf.utils.database.Table getEcTable()
```

:   Creates the table for the ec numbers and ancestors

    :   **Returns:**: the table

---


### getTableManager

```
public uk.ac.ed.inf.utils.database.TableManager getTableManager()
```

---


### writeEcAndAncestorsToTable

```
public void writeEcAndAncestorsToTable()
```

---


### main

```
public static void main(java.lang.String[] args)
                 throws java.sql.SQLException
```

:   Main for recreating table

    :   **Parameters:**: `args` - **Throws:**: `java.sql.SQLException`


---


|  |  |  |  |  |  |  |  |  |  |  |
| --- | --- | --- | --- | --- | --- | --- | --- | --- | --- | --- |
| |  |  |  |  |  |  |  |  | | --- | --- | --- | --- | --- | --- | --- | --- | | **Overview** | **Package** | **Class** | **Use** | **Tree** | **Deprecated** | **Index** | **Help** | | |  |
| **PREV CLASS**   **NEXT CLASS** | **FRAMES**    **NO FRAMES**     **All Classes** |
| SUMMARY: NESTED | FIELD | CONSTR | METHOD | DETAIL: FIELD | CONSTR | METHOD |


---
