# Supplementary material for: From sequence to enzyme mechanism using multi-label machine learning
Source: BMC Bioinformatics. 2014 May 19;15:150. doi: 10.1186/1471-2105-15-150 (PMC4229970; doi:10.1186/1471-2105-15-150)
Supplement: Additional file 2 — Java code of ml2db. Additional file ml2db_code.tar.gz contains the Java source code to run the multi-label machine learning experiments and save the results to database. The code’s Javadoc is included. [file 1471-2105-15-150-S2.zip › additional file 2/ml2db/ecmulan/doc/uk/ac/ed/inf/ec/EcFullXmlCreator.html]

EcFullXmlCreator


---


|  |  |  |  |  |  |  |  |  |  |  |
| --- | --- | --- | --- | --- | --- | --- | --- | --- | --- | --- |
| |  |  |  |  |  |  |  |  | | --- | --- | --- | --- | --- | --- | --- | --- | | **Overview** | **Package** | **Class** | **Use** | **Tree** | **Deprecated** | **Index** | **Help** | | |  |
| **PREV CLASS**   **NEXT CLASS** | **FRAMES**    **NO FRAMES**     **All Classes** |
| SUMMARY: NESTED | FIELD | CONSTR | METHOD | DETAIL: FIELD | CONSTR | METHOD |


---


## uk.ac.ed.inf.ec Class EcFullXmlCreator

```
java.lang.Object
  uk.ac.ed.inf.ec.EcFullXmlCreator
```

**Direct Known Subclasses:**: EcMulanXmlCreator

---

``` public class EcFullXmlCreator extends java.lang.Object ```

Creates a full XML hierarchical representation of Enzyme Commission numbers
in Mulan format. Note: it creates also EC numbers that might not be present
in the original list, but are ancestors of the given numbers. The ancestor of
all EC numbers is the pseudo-EC numbers "-.-.-.-" (4 dashes)

**Version:**
:   4 Nov 2010

**Author:**
:   Luna De Ferrari luna.deferrari-at-ed.ac.uk

---

| **Constructor Summary** | |
| --- | --- |
| `EcFullXmlCreator(java.lang.String dbConnPath, java.lang.String getEcQuery)`             Get EC numbers from database |
| `EcFullXmlCreator(java.util.TreeSet<java.lang.String> ecNumberStrings)`             Get EC numbers from list |


| **Method Summary** | |
| --- | --- |
| `java.lang.String` | `createXmlString()`             Generate an XML representation of the Enzyme Commission number hierarchy in Mulan format. |
| `EcDbReader` | `getDbReader()` |
| `java.util.TreeSet<EcNumber>` | `getEcNumbers()` |
| `java.lang.String` | `getXmlString()`             Adds a log to the xml file (timestamp, database and query to the data etc.) |
| `static void` | `main(java.lang.String[] args)` |
| `void` | `saveToFile(java.lang.String filePath)` |

| **Methods inherited from class java.lang.Object** |
| --- |
| `equals, getClass, hashCode, notify, notifyAll, toString, wait, wait, wait` |

| **Constructor Detail** |
| --- |

### EcFullXmlCreator

```
public EcFullXmlCreator(java.lang.String dbConnPath,
                        java.lang.String getEcQuery)
```

:   Get EC numbers from database

    **Parameters:**: `dbConnPropsPath` -

---


### EcFullXmlCreator

```
public EcFullXmlCreator(java.util.TreeSet<java.lang.String> ecNumberStrings)
```

:   Get EC numbers from list

    **Parameters:**: `ecNumberStrings` -


| **Method Detail** |
| --- |

### createXmlString

```
public java.lang.String createXmlString()
```

:   Generate an XML representation of the Enzyme Commission number hierarchy
    in Mulan format.

    :   **Returns:**: an XML representation of the Enzyme Commission number hierarchy in Mulan format.

---


### getDbReader

```
public EcDbReader getDbReader()
```

---


### getEcNumbers

```
public java.util.TreeSet<EcNumber> getEcNumbers()
```

---


### getXmlString

```
public java.lang.String getXmlString()
```

:   Adds a log to the xml file (timestamp, database and query to the data
    etc.)

    :   **Returns:**

---


### saveToFile

```
public void saveToFile(java.lang.String filePath)
```

---


### main

```
public static void main(java.lang.String[] args)
```


---


|  |  |  |  |  |  |  |  |  |  |  |
| --- | --- | --- | --- | --- | --- | --- | --- | --- | --- | --- |
| |  |  |  |  |  |  |  |  | | --- | --- | --- | --- | --- | --- | --- | --- | | **Overview** | **Package** | **Class** | **Use** | **Tree** | **Deprecated** | **Index** | **Help** | | |  |
| **PREV CLASS**   **NEXT CLASS** | **FRAMES**    **NO FRAMES**     **All Classes** |
| SUMMARY: NESTED | FIELD | CONSTR | METHOD | DETAIL: FIELD | CONSTR | METHOD |


---
