# Supplementary material for: From sequence to enzyme mechanism using multi-label machine learning
Source: BMC Bioinformatics. 2014 May 19;15:150. doi: 10.1186/1471-2105-15-150 (PMC4229970; doi:10.1186/1471-2105-15-150)
Supplement: Additional file 2 — Java code of ml2db. Additional file ml2db_code.tar.gz contains the Java source code to run the multi-label machine learning experiments and save the results to database. The code’s Javadoc is included. [file 1471-2105-15-150-S2.zip › additional file 2/ml2db/ecmulan/doc/uk/ac/ed/inf/ec/EcMulanXmlCreator.html]

EcMulanXmlCreator


---


|  |  |  |  |  |  |  |  |  |  |  |
| --- | --- | --- | --- | --- | --- | --- | --- | --- | --- | --- |
| |  |  |  |  |  |  |  |  | | --- | --- | --- | --- | --- | --- | --- | --- | | **Overview** | **Package** | **Class** | **Use** | **Tree** | **Deprecated** | **Index** | **Help** | | |  |
| **PREV CLASS**   **NEXT CLASS** | **FRAMES**    **NO FRAMES**     **All Classes** |
| SUMMARY: NESTED | FIELD | CONSTR | METHOD | DETAIL: FIELD | CONSTR | METHOD |


---


## uk.ac.ed.inf.ec Class EcMulanXmlCreator

```
java.lang.Object
  uk.ac.ed.inf.ec.EcFullXmlCreator
      uk.ac.ed.inf.ec.EcMulanXmlCreator
```

---

``` public class EcMulanXmlCreator extends EcFullXmlCreator ```

Creates a full XML hierarchical representation of Enzyme Commission numbers
in Mulan format.

**Version:**
:   2 Jun 2010

**Author:**
:   Luna De Ferrari luna.deferrari-at-ed.ac.uk

---

| **Field Summary** | |
| --- | --- |
| `static java.lang.String` | `DATA_DATABASE` |


| **Constructor Summary** | |
| --- | --- |
| `EcMulanXmlCreator(java.lang.String dbConnPath, java.lang.String getEcQuery)` |
| `EcMulanXmlCreator(java.util.TreeSet<java.lang.String> ecNumberStrings)` |


| **Method Summary** | |
| --- | --- |
| `java.lang.String` | `createXmlString()`             Generate an XML representation of the Enzyme Commission number hierarchy in Mulan format. |
| `static void` | `main(java.lang.String[] args)` |

| **Methods inherited from class uk.ac.ed.inf.ec.EcFullXmlCreator** |
| --- |
| `getDbReader, getEcNumbers, getXmlString, saveToFile` |

| **Methods inherited from class java.lang.Object** |
| --- |
| `equals, getClass, hashCode, notify, notifyAll, toString, wait, wait, wait` |

| **Field Detail** |
| --- |

### DATA\_DATABASE

```
public static java.lang.String DATA_DATABASE
```


| **Constructor Detail** |
| --- |

### EcMulanXmlCreator

```
public EcMulanXmlCreator(java.lang.String dbConnPath,
                         java.lang.String getEcQuery)
```

---


### EcMulanXmlCreator

```
public EcMulanXmlCreator(java.util.TreeSet<java.lang.String> ecNumberStrings)
```


| **Method Detail** |
| --- |

### createXmlString

```
public java.lang.String createXmlString()
```

:   **Description copied from class: `EcFullXmlCreator`**
:   Generate an XML representation of the Enzyme Commission number hierarchy
    in Mulan format.

    :   **Overrides:**: `createXmlString` in class `EcFullXmlCreator`
    :   **Returns:**: an XML representation of the Enzyme Commission number hierarchy in Mulan format.

---


### main

```
public static void main(java.lang.String[] args)
```


---


|  |  |  |  |  |  |  |  |  |  |  |
| --- | --- | --- | --- | --- | --- | --- | --- | --- | --- | --- |
| |  |  |  |  |  |  |  |  | | --- | --- | --- | --- | --- | --- | --- | --- | | **Overview** | **Package** | **Class** | **Use** | **Tree** | **Deprecated** | **Index** | **Help** | | |  |
| **PREV CLASS**   **NEXT CLASS** | **FRAMES**    **NO FRAMES**     **All Classes** |
| SUMMARY: NESTED | FIELD | CONSTR | METHOD | DETAIL: FIELD | CONSTR | METHOD |


---
