# Supplementary material for: From sequence to enzyme mechanism using multi-label machine learning
Source: BMC Bioinformatics. 2014 May 19;15:150. doi: 10.1186/1471-2105-15-150 (PMC4229970; doi:10.1186/1471-2105-15-150)
Supplement: Additional file 2 — Java code of ml2db. Additional file ml2db_code.tar.gz contains the Java source code to run the multi-label machine learning experiments and save the results to database. The code’s Javadoc is included. [file 1471-2105-15-150-S2.zip › additional file 2/ml2db/ecmulan/doc/uk/ac/ed/inf/ec/EcNumber.html]

EcNumber


---


|  |  |  |  |  |  |  |  |  |  |  |
| --- | --- | --- | --- | --- | --- | --- | --- | --- | --- | --- |
| |  |  |  |  |  |  |  |  | | --- | --- | --- | --- | --- | --- | --- | --- | | **Overview** | **Package** | **Class** | **Use** | **Tree** | **Deprecated** | **Index** | **Help** | | |  |
| **PREV CLASS**   **NEXT CLASS** | **FRAMES**    **NO FRAMES**     **All Classes** |
| SUMMARY: NESTED | FIELD | CONSTR | METHOD | DETAIL: FIELD | CONSTR | METHOD |


---


## uk.ac.ed.inf.ec Class EcNumber

```
java.lang.Object
  uk.ac.ed.inf.ec.EcNumber
```

**All Implemented Interfaces:**: java.lang.Comparable<EcNumber>

---

``` public class EcNumber extends java.lang.Object implements java.lang.Comparable<EcNumber> ```

A class representing an Enzyme Commission (EC) number. The enzyme commission
has set a standard nomenclature (4 level deep numerical hierarchy) of
enzymatic reactions. More at: http://www.chem.qmul.ac.uk/iubmb/enzyme/
An example of complete EC number is: 1.2.3.4 Incomplete EC numbers are, for
example: 1.-.-.-, 1.2.-.-, 1.2.3.-

**Version:**
:   11 Nov 2010

**Author:**
:   Luna De Ferrari luna.deferrari-at-ed.ac.uk

---

| **Constructor Summary** | |
| --- | --- |
| `EcNumber(java.lang.String ec)` |


| **Method Summary** | |
| --- | --- |
| `int` | `compareTo(EcNumber otherEc)` |
| `java.lang.String[]` | `getAncestorsStrings()`             Get a complete hierarchy of ancestor for the ec number. |
| `java.lang.String[]` | `getBlocks()` |
| `java.lang.String` | `getEcString()` |
| `static java.lang.String` | `getEcStringFromBlocks(java.lang.String[] blocks)` |
| `int` | `getHierarchyLevel()` |
| `EcNumber` | `getParent()` |
| `java.lang.String` | `getParentString()` |
| `boolean` | `isComplete()` |
| `boolean` | `isParent(EcNumber possibleChild)`             Returns true if the node given is a direct child of this node: e.g: 1.2.3.4 is direct child of 1.2.3.-, but it is not direct child of 1.2.-.- |

| **Methods inherited from class java.lang.Object** |
| --- |
| `equals, getClass, hashCode, notify, notifyAll, toString, wait, wait, wait` |

| **Constructor Detail** |
| --- |

### EcNumber

```
public EcNumber(java.lang.String ec)
```


| **Method Detail** |
| --- |

### compareTo

```
public int compareTo(EcNumber otherEc)
```

:   **Specified by:**: `compareTo` in interface `java.lang.Comparable<EcNumber>`

---


### getAncestorsStrings

```
public java.lang.String[] getAncestorsStrings()
```

:   Get a complete hierarchy of ancestor for the ec number. For EC 1.2.3.4 it
    is [1.-.-.-, 1.2.---, 1.2.3.-, 1.2.3.4] For EC 1.2.-.- it is [1.-.-.-,
    1.2.---, null, null]

---


### getBlocks

```
public java.lang.String[] getBlocks()
```

---


### getEcString

```
public java.lang.String getEcString()
```

---


### getHierarchyLevel

```
public int getHierarchyLevel()
```

---


### getParent

```
public EcNumber getParent()
```

---


### getParentString

```
public java.lang.String getParentString()
```

---


### isComplete

```
public boolean isComplete()
```

---


### isParent

```
public boolean isParent(EcNumber possibleChild)
```

:   Returns true if the node given is a direct child of this node: e.g:
    1.2.3.4 is direct child of 1.2.3.-, but it is not direct child of 1.2.-.-

    :   **Parameters:**: `possibleChild` - **Returns:**: true if the given node is a direct child of this node

---


### getEcStringFromBlocks

```
public static java.lang.String getEcStringFromBlocks(java.lang.String[] blocks)
```


---


|  |  |  |  |  |  |  |  |  |  |  |
| --- | --- | --- | --- | --- | --- | --- | --- | --- | --- | --- |
| |  |  |  |  |  |  |  |  | | --- | --- | --- | --- | --- | --- | --- | --- | | **Overview** | **Package** | **Class** | **Use** | **Tree** | **Deprecated** | **Index** | **Help** | | |  |
| **PREV CLASS**   **NEXT CLASS** | **FRAMES**    **NO FRAMES**     **All Classes** |
| SUMMARY: NESTED | FIELD | CONSTR | METHOD | DETAIL: FIELD | CONSTR | METHOD |


---
