# Supplementary material for: From sequence to enzyme mechanism using multi-label machine learning
Source: BMC Bioinformatics. 2014 May 19;15:150. doi: 10.1186/1471-2105-15-150 (PMC4229970; doi:10.1186/1471-2105-15-150)
Supplement: Additional file 2 — Java code of ml2db. Additional file ml2db_code.tar.gz contains the Java source code to run the multi-label machine learning experiments and save the results to database. The code’s Javadoc is included. [file 1471-2105-15-150-S2.zip › additional file 2/ml2db/ecmulan/doc/uk/ac/ed/inf/ec/EcNumberGenerator.html]

EcNumberGenerator


---


|  |  |  |  |  |  |  |  |  |  |  |
| --- | --- | --- | --- | --- | --- | --- | --- | --- | --- | --- |
| |  |  |  |  |  |  |  |  | | --- | --- | --- | --- | --- | --- | --- | --- | | **Overview** | **Package** | **Class** | **Use** | **Tree** | **Deprecated** | **Index** | **Help** | | |  |
| **PREV CLASS**   **NEXT CLASS** | **FRAMES**    **NO FRAMES**     **All Classes** |
| SUMMARY: NESTED | FIELD | CONSTR | METHOD | DETAIL: FIELD | CONSTR | METHOD |


---


## uk.ac.ed.inf.ec Class EcNumberGenerator

```
java.lang.Object
  uk.ac.ed.inf.ec.EcNumberGenerator
```

---

``` public class EcNumberGenerator extends java.lang.Object ```

Given a string such as 1.2.3.4 or 1.2.-.-.- checks if it is a valid Enzyme
commission number and generates the Java EcNumber object (including its
parent EC numbers)

**Version:**
:   8 Jun 2010

**Author:**
:   Luna De Ferrari luna.deferrari-at-ed.ac.uk

---

| **Field Summary** | |
| --- | --- |
| `static java.lang.String` | `DASH`             the substitute for an unknown digit blocks (a dash) |
| `static java.lang.String` | `DOT`             the separator for the digit blocks (dot) |
| `static int` | `FULL_HIERARCHY_LENGHT`             the number of levels in the EC number parent-child hierarchy (if -.-.-.- is included = 5, traditional ec hierarchy = 4 ) |
| `static java.lang.String` | `MAX_LEVEL1_CLASS`             maximum value for level 1 class: currently 6. |
| `static java.lang.String` | `MAX_LEVEL1_REGEXP`             regexp from 1 to 6 |
| `static java.lang.String` | `MAX_LEVEL2_CLASS`             maximum value for level 2 class: 99 in this implementation. |
| `static java.lang.String` | `MAX_LEVEL2_REGEXP`             regexp from 0 to 99 |
| `static java.lang.String` | `MAX_LEVEL3_CLASS`             maximum value for level 3 class: 99 in this implementation. |
| `static java.lang.String` | `MAX_LEVEL3_REGEXP`             regexp from 0 to 99 |
| `static java.lang.String` | `MAX_LEVEL4_CLASS`             maximum value for level 4 class: 999 in this implementation. |
| `static java.lang.String` | `MAX_LEVEL4_REGEXP`             regexp from 0 to 999 |


| **Constructor Summary** | |
| --- | --- |
| `EcNumberGenerator(java.lang.String ec)` |


| **Method Summary** | |
| --- | --- |
| `static boolean` | `couldBeDashedEc(java.lang.String string)`             Checks whether the string could contain a validly formatted ec number with dashes (1.-.-.-, 1.2.3.- etc., but not 1.-.3.4) |
| `static boolean` | `couldBeEc(java.lang.String string)`             Checks if the string could contain a validly formatted EC number. |
| `static EcNumber` | `generateEcNumber(java.lang.String ec)` |
| `static boolean` | `isValidEcBlock(java.lang.String block)`             True if the string contains a dash '-' or a positive integer or a positive integer preceded by 'n' (new uniprot ec numbers) |

| **Methods inherited from class java.lang.Object** |
| --- |
| `equals, getClass, hashCode, notify, notifyAll, toString, wait, wait, wait` |

| **Field Detail** |
| --- |

### DASH

```
public static final java.lang.String DASH
```

:   the substitute for an unknown digit blocks (a dash)

    **See Also:**: Constant Field Values

---


### DOT

```
public static final java.lang.String DOT
```

:   the separator for the digit blocks (dot)

    **See Also:**: Constant Field Values

---


### MAX\_LEVEL1\_CLASS

```
public static final java.lang.String MAX_LEVEL1_CLASS
```

:   maximum value for level 1 class: currently 6. No EC number can start with
    7.-.-.-

    **See Also:**: Constant Field Values

---


### MAX\_LEVEL1\_REGEXP

```
public static final java.lang.String MAX_LEVEL1_REGEXP
```

:   regexp from 1 to 6

    **See Also:**: Constant Field Values

---


### MAX\_LEVEL2\_CLASS

```
public static final java.lang.String MAX_LEVEL2_CLASS
```

:   maximum value for level 2 class: 99 in this implementation. No EC number
    can have 1.100.-.-

    **See Also:**: Constant Field Values

---


### MAX\_LEVEL2\_REGEXP

```
public static final java.lang.String MAX_LEVEL2_REGEXP
```

:   regexp from 0 to 99

    **See Also:**: Constant Field Values

---


### MAX\_LEVEL3\_CLASS

```
public static final java.lang.String MAX_LEVEL3_CLASS
```

:   maximum value for level 3 class: 99 in this implementation. No EC number
    can have 1.1.100.-

    **See Also:**: Constant Field Values

---


### MAX\_LEVEL3\_REGEXP

```
public static final java.lang.String MAX_LEVEL3_REGEXP
```

:   regexp from 0 to 99

    **See Also:**: Constant Field Values

---


### MAX\_LEVEL4\_CLASS

```
public static final java.lang.String MAX_LEVEL4_CLASS
```

:   maximum value for level 4 class: 999 in this implementation.

    **See Also:**: Constant Field Values

---


### MAX\_LEVEL4\_REGEXP

```
public static final java.lang.String MAX_LEVEL4_REGEXP
```

:   regexp from 0 to 999

    **See Also:**: Constant Field Values

---


### FULL\_HIERARCHY\_LENGHT

```
public static final int FULL_HIERARCHY_LENGHT
```

:   the number of levels in the EC number parent-child hierarchy (if -.-.-.-
    is included = 5, traditional ec hierarchy = 4 )

    **See Also:**: Constant Field Values


| **Constructor Detail** |
| --- |

### EcNumberGenerator

```
public EcNumberGenerator(java.lang.String ec)
```

**Parameters:**: `ec` - the ec number string


| **Method Detail** |
| --- |

### couldBeDashedEc

```
public static boolean couldBeDashedEc(java.lang.String string)
```

:   Checks whether the string could contain a validly formatted ec number
    with dashes (1.-.-.-, 1.2.3.- etc., but not 1.-.3.4)

    :   **Parameters:**: `string` - the ec string **Returns:**: true if the format is valid

---


### couldBeEc

```
public static boolean couldBeEc(java.lang.String string)
```

:   Checks if the string could contain a validly formatted EC number.

    :   **Parameters:**: `string` - the ec number string **Returns:**: true if the format is compatible with an EC number

---


### generateEcNumber

```
public static EcNumber generateEcNumber(java.lang.String ec)
```

---


### isValidEcBlock

```
public static boolean isValidEcBlock(java.lang.String block)
```

:   True if the string contains a dash '-' or a positive integer or a
    positive integer preceded by 'n' (new uniprot ec numbers)


---


|  |  |  |  |  |  |  |  |  |  |  |
| --- | --- | --- | --- | --- | --- | --- | --- | --- | --- | --- |
| |  |  |  |  |  |  |  |  | | --- | --- | --- | --- | --- | --- | --- | --- | | **Overview** | **Package** | **Class** | **Use** | **Tree** | **Deprecated** | **Index** | **Help** | | |  |
| **PREV CLASS**   **NEXT CLASS** | **FRAMES**    **NO FRAMES**     **All Classes** |
| SUMMARY: NESTED | FIELD | CONSTR | METHOD | DETAIL: FIELD | CONSTR | METHOD |


---
