# Supplementary material for: From sequence to enzyme mechanism using multi-label machine learning
Source: BMC Bioinformatics. 2014 May 19;15:150. doi: 10.1186/1471-2105-15-150 (PMC4229970; doi:10.1186/1471-2105-15-150)
Supplement: Additional file 2 — Java code of ml2db. Additional file ml2db_code.tar.gz contains the Java source code to run the multi-label machine learning experiments and save the results to database. The code’s Javadoc is included. [file 1471-2105-15-150-S2.zip › additional file 2/ml2db/ecmulan/doc/uk/ac/ed/inf/ec/MulanLabel.html]

MulanLabel


---


|  |  |  |  |  |  |  |  |  |  |  |
| --- | --- | --- | --- | --- | --- | --- | --- | --- | --- | --- |
| |  |  |  |  |  |  |  |  | | --- | --- | --- | --- | --- | --- | --- | --- | | **Overview** | **Package** | **Class** | **Use** | **Tree** | **Deprecated** | **Index** | **Help** | | |  |
| **PREV CLASS**   **NEXT CLASS** | **FRAMES**    **NO FRAMES**     **All Classes** |
| SUMMARY: NESTED | FIELD | CONSTR | METHOD | DETAIL: FIELD | CONSTR | METHOD |


---


## uk.ac.ed.inf.ec Class MulanLabel

```
java.lang.Object
  uk.ac.ed.inf.utils.webutils.simpledomparser.XmlNode
      uk.ac.ed.inf.ec.MulanLabel
```

---

``` public class MulanLabel extends uk.ac.ed.inf.utils.webutils.simpledomparser.XmlNode ```

A node in the Mulan XML (a label for machine learning) \*

```
Version:
:   5 May 2010


Author:
:   Luna De Ferrari luna.deferrari-at-ed.ac.uk


---


| Constructor Summary | |
| --- | --- |
| MulanLabel(java.lang.String labelName) |


| Method Summary | |
| --- | --- |
| void | addChildElement(MulanLabel label)              Add a child label to a label |
| void | addChildElement(java.lang.String labelName)              Add a child label to a label |


| Methods inherited from class uk.ac.ed.inf.utils.webutils.simpledomparser.XmlNode |
| --- |
| addChildNode, getAttributeValue, getChildElements, getTagName, getText, getXmlTreeFromString, getXmlTreeFromUrl, hasChildren, nodeHeadToString, removeAttribute, removeChildNode, setAttribute, setTagName, setText, toString |


| Methods inherited from class java.lang.Object |
| --- |
| equals, getClass, hashCode, notify, notifyAll, wait, wait, wait |


| Constructor Detail |
| --- |


### MulanLabel


```
public MulanLabel(java.lang.String labelName)
```


| Method Detail |
| --- |


### addChildElement


```
public void addChildElement(MulanLabel label)
```


:   Add a child label to a label

    :   Parameters:: label - the child label (with its children etc.)


---


### addChildElement


```
public void addChildElement(java.lang.String labelName)
```


:   Add a child label to a label

    :   Parameters:: labelName - the name of the label to be added


---


|  |  |  |  |  |  |  |  |  |  |  |
| --- | --- | --- | --- | --- | --- | --- | --- | --- | --- | --- |
| |  |  |  |  |  |  |  |  | | --- | --- | --- | --- | --- | --- | --- | --- | | Overview | Package | Class | Use | Tree | Deprecated | Index | Help | | |  |
| PREV CLASS   NEXT CLASS | FRAMES     NO FRAMES          All Classes |
| SUMMARY: NESTED | FIELD | CONSTR | METHOD | DETAIL: FIELD | CONSTR | METHOD |


---
```
