# Supplementary material for: From sequence to enzyme mechanism using multi-label machine learning
Source: BMC Bioinformatics. 2014 May 19;15:150. doi: 10.1186/1471-2105-15-150 (PMC4229970; doi:10.1186/1471-2105-15-150)
Supplement: Additional file 2 — Java code of ml2db. Additional file ml2db_code.tar.gz contains the Java source code to run the multi-label machine learning experiments and save the results to database. The code’s Javadoc is included. [file 1471-2105-15-150-S2.zip › additional file 2/ml2db/ecmulan/doc/uk/ac/ed/inf/ec/MulanXml.html]

MulanXml


---


|  |  |  |  |  |  |  |  |  |  |  |
| --- | --- | --- | --- | --- | --- | --- | --- | --- | --- | --- |
| |  |  |  |  |  |  |  |  | | --- | --- | --- | --- | --- | --- | --- | --- | | **Overview** | **Package** | **Class** | **Use** | **Tree** | **Deprecated** | **Index** | **Help** | | |  |
| **PREV CLASS**   NEXT CLASS | **FRAMES**    **NO FRAMES**     **All Classes** |
| SUMMARY: NESTED | FIELD | CONSTR | METHOD | DETAIL: FIELD | CONSTR | METHOD |


---


## uk.ac.ed.inf.ec Class MulanXml

```
java.lang.Object
  uk.ac.ed.inf.ec.MulanXml
```

---

``` public class MulanXml extends java.lang.Object ```

Generates an XML file for labels in the Mulan format
http://mulan.sourceforge.net/ http://mlkd.csd.auth.gr/multilabel.html

Example of Mulan XML format for labels:

```
 labels xmlns="http://mulan.sourceforge.net/labels"> 
 <label name="label1">  
 	<label name="label12"></label>  
 	<label name="label13"></label>  
 	<label name="label14"></label>  
 	<label name="label15"></label> 
 </label>
 /labels>
```

**Version:**
:   30 Apr 2010

**Author:**
:   Luna De Ferrari luna.deferrari-at-ed.ac.uk

---

| **Field Summary** | |
| --- | --- |
| `static java.lang.String` | `LABEL_NAME_ATTRIBUTE`             the label tag name attribute |
| `static java.lang.String` | `LABEL_XML_TAG`             the label tag |
| `MulanLabel` | `m_root`             the xml tree root |
| `static java.lang.String` | `MULAN_XML_ROOT_TAG` |
| `static java.lang.String` | `MULAN_XML_ROOT_TAG_ATTRIBUTE_NAME` |
| `static java.lang.String` | `MULAN_XML_ROOT_TAG_ATTRIBUTE_VALUE` |


| **Constructor Summary** | |
| --- | --- |
| `MulanXml()` |


| **Method Summary** | |
| --- | --- |
| `MulanLabel` | `findNode(java.lang.String label)`             Find a node by label |
| `MulanLabel` | `getRoot()` |
| `java.lang.String` | `toString()`             public MulanLabel removeNode(String label) { // find node MulanLabel node = this.findNode(label); // get parent MulanLabel parent = node. |

| **Methods inherited from class java.lang.Object** |
| --- |
| `equals, getClass, hashCode, notify, notifyAll, wait, wait, wait` |

| **Field Detail** |
| --- |

### LABEL\_NAME\_ATTRIBUTE

```
public static final java.lang.String LABEL_NAME_ATTRIBUTE
```

:   the label tag name attribute

    **See Also:**: Constant Field Values

---


### LABEL\_XML\_TAG

```
public static final java.lang.String LABEL_XML_TAG
```

:   the label tag

    **See Also:**: Constant Field Values

---


### MULAN\_XML\_ROOT\_TAG

```
public static final java.lang.String MULAN_XML_ROOT_TAG
```

**See Also:**: Constant Field Values

---


### MULAN\_XML\_ROOT\_TAG\_ATTRIBUTE\_NAME

```
public static final java.lang.String MULAN_XML_ROOT_TAG_ATTRIBUTE_NAME
```

**See Also:**: Constant Field Values

---


### MULAN\_XML\_ROOT\_TAG\_ATTRIBUTE\_VALUE

```
public static final java.lang.String MULAN_XML_ROOT_TAG_ATTRIBUTE_VALUE
```

**See Also:**: Constant Field Values

---


### m\_root

```
public MulanLabel m_root
```

:   the xml tree root


| **Constructor Detail** |
| --- |

### MulanXml

```
public MulanXml()
```


| **Method Detail** |
| --- |

### findNode

```
public MulanLabel findNode(java.lang.String label)
```

:   Find a node by label

    :   **Parameters:**: `label` - the node label (the name attribute value), such as 'abc' in ``` <label name="abc"></label> ``` **Returns:**: the node with that label, or null if not found

---


### getRoot

```
public MulanLabel getRoot()
```

---


### toString

```
public java.lang.String toString()
```

:   public MulanLabel removeNode(String label) { // find node MulanLabel node
    = this.findNode(label); // get parent MulanLabel parent = node. // remove
    child node }

    :   **Overrides:**: `toString` in class `java.lang.Object`


---


|  |  |  |  |  |  |  |  |  |  |  |
| --- | --- | --- | --- | --- | --- | --- | --- | --- | --- | --- |
| |  |  |  |  |  |  |  |  | | --- | --- | --- | --- | --- | --- | --- | --- | | **Overview** | **Package** | **Class** | **Use** | **Tree** | **Deprecated** | **Index** | **Help** | | |  |
| **PREV CLASS**   NEXT CLASS | **FRAMES**    **NO FRAMES**     **All Classes** |
| SUMMARY: NESTED | FIELD | CONSTR | METHOD | DETAIL: FIELD | CONSTR | METHOD |


---
