# Supplementary material for: From sequence to enzyme mechanism using multi-label machine learning
Source: BMC Bioinformatics. 2014 May 19;15:150. doi: 10.1186/1471-2105-15-150 (PMC4229970; doi:10.1186/1471-2105-15-150)
Supplement: Additional file 2 — Java code of ml2db. Additional file ml2db_code.tar.gz contains the Java source code to run the multi-label machine learning experiments and save the results to database. The code’s Javadoc is included. [file 1471-2105-15-150-S2.zip › additional file 2/ml2db/ecmulan/doc/uk/ac/ed/inf/ec/package-tree.html]

uk.ac.ed.inf.ec Class Hierarchy


---


|  |  |  |  |  |  |  |  |  |  |  |
| --- | --- | --- | --- | --- | --- | --- | --- | --- | --- | --- |
| |  |  |  |  |  |  |  |  | | --- | --- | --- | --- | --- | --- | --- | --- | | **Overview** | **Package** | Class | Use | **Tree** | **Deprecated** | **Index** | **Help** | | |  |
| PREV   **NEXT** | **FRAMES**    **NO FRAMES**     **All Classes** |


---


## Hierarchy For Package uk.ac.ed.inf.ec

**Package Hierarchies:**: All Packages

---

## Class Hierarchy

- java.lang.Object
  - uk.ac.ed.inf.utils.database.DbManaged
    - uk.ac.ed.inf.utils.database.DbReader<T,U>
      - uk.ac.ed.inf.ec.**EcDbReader**- uk.ac.ed.inf.ec.**EcDbWriter**- uk.ac.ed.inf.ec.**EcFullXmlCreator**
      - uk.ac.ed.inf.ec.**EcMulanXmlCreator**- uk.ac.ed.inf.ec.**EcNumber** (implements java.lang.Comparable<T>)- uk.ac.ed.inf.ec.**EcNumberGenerator**- uk.ac.ed.inf.ec.**MulanXml**- uk.ac.ed.inf.utils.webutils.simpledomparser.XmlNode
              - uk.ac.ed.inf.ec.**MulanLabel**

---


|  |  |  |  |  |  |  |  |  |  |  |
| --- | --- | --- | --- | --- | --- | --- | --- | --- | --- | --- |
| |  |  |  |  |  |  |  |  | | --- | --- | --- | --- | --- | --- | --- | --- | | **Overview** | **Package** | Class | Use | **Tree** | **Deprecated** | **Index** | **Help** | | |  |
| PREV   **NEXT** | **FRAMES**    **NO FRAMES**     **All Classes** |


---
