# Supplementary material for: From sequence to enzyme mechanism using multi-label machine learning
Source: BMC Bioinformatics. 2014 May 19;15:150. doi: 10.1186/1471-2105-15-150 (PMC4229970; doi:10.1186/1471-2105-15-150)
Supplement: Additional file 2 — Java code of ml2db. Additional file ml2db_code.tar.gz contains the Java source code to run the multi-label machine learning experiments and save the results to database. The code’s Javadoc is included. [file 1471-2105-15-150-S2.zip › additional file 2/ml2db/ecmulan/doc/uk/ac/ed/inf/ec/package-use.html]

Uses of Package uk.ac.ed.inf.ec


---


|  |  |  |  |  |  |  |  |  |  |  |
| --- | --- | --- | --- | --- | --- | --- | --- | --- | --- | --- |
| |  |  |  |  |  |  |  |  | | --- | --- | --- | --- | --- | --- | --- | --- | | **Overview** | **Package** | Class | **Use** | **Tree** | **Deprecated** | **Index** | **Help** | | |  |
| PREV   NEXT | **FRAMES**    **NO FRAMES**     **All Classes** |


---


## **Uses of Package uk.ac.ed.inf.ec**

| Packages that use uk.ac.ed.inf.ec | |
| --- | --- |
| **uk.ac.ed.inf.ec** |  |
| **uk.ac.ed.inf.ec.test** |  |

| Classes in uk.ac.ed.inf.ec used by uk.ac.ed.inf.ec | |
| --- | --- |
| ****EcDbReader****             Reads the full list of Enzyme Commission numbers from database |
| ****EcFullXmlCreator****             Creates a full XML hierarchical representation of Enzyme Commission numbers in Mulan format. |
| ****EcNumber****             A class representing an Enzyme Commission (EC) number. |
| ****MulanLabel****             A node in the Mulan XML (a label for machine learning) \* |

| Classes in uk.ac.ed.inf.ec used by uk.ac.ed.inf.ec.test | |
| --- | --- |
| ****EcDbWriter****             Given a database and a list of Enzyme commission numbers, writes a 2 columns table containing: in column 1: the Ec number, in column 2: all the ancestors of that EC number, including itself. |
| ****EcFullXmlCreator****             Creates a full XML hierarchical representation of Enzyme Commission numbers in Mulan format. |
| ****EcMulanXmlCreator****             Creates a full XML hierarchical representation of Enzyme Commission numbers in Mulan format. |
| ****EcNumber****             A class representing an Enzyme Commission (EC) number. |
| ****MulanLabel****             A node in the Mulan XML (a label for machine learning) \* |
| ****MulanXml****             Generates an XML file for labels in the Mulan format http://mulan.sourceforge.net/ http://mlkd.csd.auth.gr/multilabel.html |

---


|  |  |  |  |  |  |  |  |  |  |  |
| --- | --- | --- | --- | --- | --- | --- | --- | --- | --- | --- |
| |  |  |  |  |  |  |  |  | | --- | --- | --- | --- | --- | --- | --- | --- | | **Overview** | **Package** | Class | **Use** | **Tree** | **Deprecated** | **Index** | **Help** | | |  |
| PREV   NEXT | **FRAMES**    **NO FRAMES**     **All Classes** |


---
