# Supplementary material for: From sequence to enzyme mechanism using multi-label machine learning
Source: BMC Bioinformatics. 2014 May 19;15:150. doi: 10.1186/1471-2105-15-150 (PMC4229970; doi:10.1186/1471-2105-15-150)
Supplement: Additional file 2 — Java code of ml2db. Additional file ml2db_code.tar.gz contains the Java source code to run the multi-label machine learning experiments and save the results to database. The code’s Javadoc is included. [file 1471-2105-15-150-S2.zip › additional file 2/ml2db/ecmulan/doc/uk/ac/ed/inf/ec/test/EcFullXmlCreatorTest.html]

EcFullXmlCreatorTest


---


|  |  |  |  |  |  |  |  |  |  |  |
| --- | --- | --- | --- | --- | --- | --- | --- | --- | --- | --- |
| |  |  |  |  |  |  |  |  | | --- | --- | --- | --- | --- | --- | --- | --- | | **Overview** | **Package** | **Class** | **Use** | **Tree** | **Deprecated** | **Index** | **Help** | | |  |
| **PREV CLASS**   **NEXT CLASS** | **FRAMES**    **NO FRAMES**     **All Classes** |
| SUMMARY: NESTED | FIELD | CONSTR | METHOD | DETAIL: FIELD | CONSTR | METHOD |


---


## uk.ac.ed.inf.ec.test Class EcFullXmlCreatorTest

```
java.lang.Object
  junit.framework.Assert
      junit.framework.TestCase
          uk.ac.ed.inf.ec.test.EcFullXmlCreatorTest
```

**All Implemented Interfaces:**: junit.framework.Test

---

``` public class EcFullXmlCreatorTest extends junit.framework.TestCase ```

Class

**Version:**
:   5 May 2010

**Author:**
:   Luna De Ferrari luna.deferrari-at-ed.ac.uk

---

| **Constructor Summary** | |
| --- | --- |
| `EcFullXmlCreatorTest()` |


| **Method Summary** | |
| --- | --- |
| `static java.util.TreeSet<java.lang.String>` | `getSet()` |
| `static EcFullXmlCreator` | `getXmlCreator0dashA()` |
| `static EcFullXmlCreator` | `getXmlCreator1dash()` |
| `static EcFullXmlCreator` | `getXmlCreator2dash()` |
| `static EcFullXmlCreator` | `getXmlCreator3dash()` |
| `static EcFullXmlCreator` | `getXmlCreator3EcNumbers()` |
| `static EcFullXmlCreator` | `getXmlCreator4dash()` |
| `static EcFullXmlCreator` | `getXmlCreatorWithDbConn()` |
| `void` | `setUp()` |
| `void` | `testEcFullXmlCreator()` |
| `void` | `testLog()` |

| **Methods inherited from class junit.framework.TestCase** |
| --- |
| `countTestCases, getName, run, run, runBare, setName, toString` |

| **Methods inherited from class junit.framework.Assert** |
| --- |
| `assertEquals, assertEquals, assertEquals, assertEquals, assertEquals, assertEquals, assertEquals, assertEquals, assertEquals, assertEquals, assertEquals, assertEquals, assertEquals, assertEquals, assertEquals, assertEquals, assertEquals, assertEquals, assertEquals, assertEquals, assertFalse, assertFalse, assertNotNull, assertNotNull, assertNotSame, assertNotSame, assertNull, assertNull, assertSame, assertSame, assertTrue, assertTrue, fail, fail, failNotEquals, failNotSame, failSame, format` |

| **Methods inherited from class java.lang.Object** |
| --- |
| `equals, getClass, hashCode, notify, notifyAll, wait, wait, wait` |

| **Constructor Detail** |
| --- |

### EcFullXmlCreatorTest

```
public EcFullXmlCreatorTest()
```


| **Method Detail** |
| --- |

### setUp

```
public void setUp()
           throws java.lang.Exception
```

:   **Overrides:**: `setUp` in class `junit.framework.TestCase`
:   **Throws:**: `java.lang.Exception`

---


### testEcFullXmlCreator

```
public void testEcFullXmlCreator()
```

---


### testLog

```
public void testLog()
```

---


### getSet

```
public static java.util.TreeSet<java.lang.String> getSet()
```

---


### getXmlCreator0dashA

```
public static EcFullXmlCreator getXmlCreator0dashA()
```

---


### getXmlCreator1dash

```
public static EcFullXmlCreator getXmlCreator1dash()
```

---


### getXmlCreator2dash

```
public static EcFullXmlCreator getXmlCreator2dash()
```

---


### getXmlCreator3dash

```
public static EcFullXmlCreator getXmlCreator3dash()
```

---


### getXmlCreator3EcNumbers

```
public static EcFullXmlCreator getXmlCreator3EcNumbers()
```

---


### getXmlCreator4dash

```
public static EcFullXmlCreator getXmlCreator4dash()
```

---


### getXmlCreatorWithDbConn

```
public static EcFullXmlCreator getXmlCreatorWithDbConn()
```


---


|  |  |  |  |  |  |  |  |  |  |  |
| --- | --- | --- | --- | --- | --- | --- | --- | --- | --- | --- |
| |  |  |  |  |  |  |  |  | | --- | --- | --- | --- | --- | --- | --- | --- | | **Overview** | **Package** | **Class** | **Use** | **Tree** | **Deprecated** | **Index** | **Help** | | |  |
| **PREV CLASS**   **NEXT CLASS** | **FRAMES**    **NO FRAMES**     **All Classes** |
| SUMMARY: NESTED | FIELD | CONSTR | METHOD | DETAIL: FIELD | CONSTR | METHOD |


---
