# Supplementary material for: From sequence to enzyme mechanism using multi-label machine learning
Source: BMC Bioinformatics. 2014 May 19;15:150. doi: 10.1186/1471-2105-15-150 (PMC4229970; doi:10.1186/1471-2105-15-150)
Supplement: Additional file 2 — Java code of ml2db. Additional file ml2db_code.tar.gz contains the Java source code to run the multi-label machine learning experiments and save the results to database. The code’s Javadoc is included. [file 1471-2105-15-150-S2.zip › additional file 2/ml2db/ecmulan/doc/uk/ac/ed/inf/ec/test/EcNumberTest.html]

EcNumberTest


---


|  |  |  |  |  |  |  |  |  |  |  |
| --- | --- | --- | --- | --- | --- | --- | --- | --- | --- | --- |
| |  |  |  |  |  |  |  |  | | --- | --- | --- | --- | --- | --- | --- | --- | | **Overview** | **Package** | **Class** | **Use** | **Tree** | **Deprecated** | **Index** | **Help** | | |  |
| **PREV CLASS**   **NEXT CLASS** | **FRAMES**    **NO FRAMES**     **All Classes** |
| SUMMARY: NESTED | FIELD | CONSTR | METHOD | DETAIL: FIELD | CONSTR | METHOD |


---


## uk.ac.ed.inf.ec.test Class EcNumberTest

```
java.lang.Object
  junit.framework.Assert
      junit.framework.TestCase
          uk.ac.ed.inf.ec.test.EcNumberTest
```

**All Implemented Interfaces:**: junit.framework.Test

---

``` public class EcNumberTest extends junit.framework.TestCase ```

Class

**Version:**
:   30 Apr 2010

**Author:**
:   Luna De Ferrari luna.deferrari-at-ed.ac.uk

---

| **Constructor Summary** | |
| --- | --- |
| `EcNumberTest()` |


| **Method Summary** | |
| --- | --- |
| `static EcNumber` | `get0dashEcA()` |
| `static EcNumber` | `get0dashEcB()` |
| `static EcNumber` | `get1dashEc()` |
| `static EcNumber` | `get2dashEc()` |
| `static EcNumber` | `get3dashEc()` |
| `static EcNumber` | `get4dashEc()` |
| `void` | `testCompareTo()` |
| `void` | `testGetBlock1()` |
| `void` | `testGetBlock2()` |
| `void` | `testGetBlock3()` |
| `void` | `testGetBlock4()` |
| `void` | `testGetHierarchy()` |
| `void` | `testGetHierarchyLevel()` |
| `void` | `testGetParent()` |
| `void` | `testIsComplete()` |
| `void` | `testIsParent()` |
| `void` | `testSplitString()` |

| **Methods inherited from class junit.framework.TestCase** |
| --- |
| `countTestCases, getName, run, run, runBare, setName, toString` |

| **Methods inherited from class junit.framework.Assert** |
| --- |
| `assertEquals, assertEquals, assertEquals, assertEquals, assertEquals, assertEquals, assertEquals, assertEquals, assertEquals, assertEquals, assertEquals, assertEquals, assertEquals, assertEquals, assertEquals, assertEquals, assertEquals, assertEquals, assertEquals, assertEquals, assertFalse, assertFalse, assertNotNull, assertNotNull, assertNotSame, assertNotSame, assertNull, assertNull, assertSame, assertSame, assertTrue, assertTrue, fail, fail, failNotEquals, failNotSame, failSame, format` |

| **Methods inherited from class java.lang.Object** |
| --- |
| `equals, getClass, hashCode, notify, notifyAll, wait, wait, wait` |

| **Constructor Detail** |
| --- |

### EcNumberTest

```
public EcNumberTest()
```


| **Method Detail** |
| --- |

### testCompareTo

```
public void testCompareTo()
```

---


### testGetBlock1

```
public void testGetBlock1()
```

---


### testGetBlock2

```
public void testGetBlock2()
```

---


### testGetBlock3

```
public void testGetBlock3()
```

---


### testGetBlock4

```
public void testGetBlock4()
```

---


### testGetHierarchy

```
public void testGetHierarchy()
```

---


### testGetHierarchyLevel

```
public void testGetHierarchyLevel()
```

---


### testGetParent

```
public void testGetParent()
```

---


### testIsComplete

```
public void testIsComplete()
```

---


### testIsParent

```
public void testIsParent()
```

---


### testSplitString

```
public void testSplitString()
```

---


### get0dashEcA

```
public static EcNumber get0dashEcA()
```

---


### get0dashEcB

```
public static EcNumber get0dashEcB()
```

---


### get1dashEc

```
public static EcNumber get1dashEc()
```

---


### get2dashEc

```
public static EcNumber get2dashEc()
```

---


### get3dashEc

```
public static EcNumber get3dashEc()
```

---


### get4dashEc

```
public static EcNumber get4dashEc()
```


---


|  |  |  |  |  |  |  |  |  |  |  |
| --- | --- | --- | --- | --- | --- | --- | --- | --- | --- | --- |
| |  |  |  |  |  |  |  |  | | --- | --- | --- | --- | --- | --- | --- | --- | | **Overview** | **Package** | **Class** | **Use** | **Tree** | **Deprecated** | **Index** | **Help** | | |  |
| **PREV CLASS**   **NEXT CLASS** | **FRAMES**    **NO FRAMES**     **All Classes** |
| SUMMARY: NESTED | FIELD | CONSTR | METHOD | DETAIL: FIELD | CONSTR | METHOD |


---
