# Supplementary material for: From sequence to enzyme mechanism using multi-label machine learning
Source: BMC Bioinformatics. 2014 May 19;15:150. doi: 10.1186/1471-2105-15-150 (PMC4229970; doi:10.1186/1471-2105-15-150)
Supplement: Additional file 2 — Java code of ml2db. Additional file ml2db_code.tar.gz contains the Java source code to run the multi-label machine learning experiments and save the results to database. The code’s Javadoc is included. [file 1471-2105-15-150-S2.zip › additional file 2/ml2db/ecmulan/doc/uk/ac/ed/inf/ec/test/package-tree.html]

uk.ac.ed.inf.ec.test Class Hierarchy


---


|  |  |  |  |  |  |  |  |  |  |  |
| --- | --- | --- | --- | --- | --- | --- | --- | --- | --- | --- |
| |  |  |  |  |  |  |  |  | | --- | --- | --- | --- | --- | --- | --- | --- | | **Overview** | **Package** | Class | Use | **Tree** | **Deprecated** | **Index** | **Help** | | |  |
| **PREV**   NEXT | **FRAMES**    **NO FRAMES**     **All Classes** |


---


## Hierarchy For Package uk.ac.ed.inf.ec.test

**Package Hierarchies:**: All Packages

---

## Class Hierarchy

- java.lang.Object
  - uk.ac.ed.inf.ec.test.**AllTests**- junit.framework.Assert
      - junit.framework.TestCase (implements junit.framework.Test)
        - uk.ac.ed.inf.ec.test.**EcDbReaderTest**- uk.ac.ed.inf.ec.test.**EcDbWriterTest**- uk.ac.ed.inf.ec.test.**EcFullXmlCreatorTest**- uk.ac.ed.inf.ec.test.**EcMulanXmlCreatorTest**- uk.ac.ed.inf.ec.test.**EcNumberGeneratorTest**- uk.ac.ed.inf.ec.test.**EcNumberTest**- uk.ac.ed.inf.ec.test.**MulanLabelTest**- uk.ac.ed.inf.ec.test.**MulanXmlTest**

---


|  |  |  |  |  |  |  |  |  |  |  |
| --- | --- | --- | --- | --- | --- | --- | --- | --- | --- | --- |
| |  |  |  |  |  |  |  |  | | --- | --- | --- | --- | --- | --- | --- | --- | | **Overview** | **Package** | Class | Use | **Tree** | **Deprecated** | **Index** | **Help** | | |  |
| **PREV**   NEXT | **FRAMES**    **NO FRAMES**     **All Classes** |


---
