# Supplementary material for: From sequence to enzyme mechanism using multi-label machine learning
Source: BMC Bioinformatics. 2014 May 19;15:150. doi: 10.1186/1471-2105-15-150 (PMC4229970; doi:10.1186/1471-2105-15-150)
Supplement: Additional file 2 — Java code of ml2db. Additional file ml2db_code.tar.gz contains the Java source code to run the multi-label machine learning experiments and save the results to database. The code’s Javadoc is included. [file 1471-2105-15-150-S2.zip › additional file 2/ml2db/ecmulan/doc/uk/ac/ed/inf/mulanxml/class-use/MulanLabel.html]

Uses of Class uk.ac.ed.inf.mulanxml.MulanLabel


JavaScript is disabled on your browser.


- Overview
- Package
- Class
- Use
- Tree
- Deprecated
- Index
- Help

- Prev
- Next

- Frames
- No Frames

- All Classes

## Uses of Class uk.ac.ed.inf.mulanxml.MulanLabel

- Packages that use MulanLabel

  | Package | Description |
  |  |  |
  | --- | --- |
  | uk.ac.ed.inf.mulanxml |  |
  | uk.ac.ed.inf.mulanxml.test |  |
- - ### Uses of MulanLabel in uk.ac.ed.inf.mulanxml

    Fields in uk.ac.ed.inf.mulanxml declared as MulanLabel

    | Modifier and Type | Field and Description |
    |  |  |
    | --- | --- |
    | `MulanLabel` | MulanXml.`m_root` the xml tree root |

    Methods in uk.ac.ed.inf.mulanxml that return MulanLabel

    | Modifier and Type | Method and Description |
    |  |  |
    | --- | --- |
    | `MulanLabel` | MulanXml.`findNode(java.lang.String label)` Find a node by label |
    | `MulanLabel` | MulanXml.`getRoot()` |

    Methods in uk.ac.ed.inf.mulanxml with parameters of type MulanLabel

    | Modifier and Type | Method and Description |
    |  |  |
    | --- | --- |
    | `void` | MulanLabel.`addChildElement(MulanLabel label)` Add a child label to a label |
  - ### Uses of MulanLabel in uk.ac.ed.inf.mulanxml.test

    Methods in uk.ac.ed.inf.mulanxml.test that return MulanLabel

    | Modifier and Type | Method and Description |
    |  |  |
    | --- | --- |
    | `static MulanLabel` | MulanLabelTest.`getMulanLabel()` |

- Overview
- Package
- Class
- Use
- Tree
- Deprecated
- Index
- Help

- Prev
- Next

- Frames
- No Frames

- All Classes
