# Supplementary material for: From sequence to enzyme mechanism using multi-label machine learning
Source: BMC Bioinformatics. 2014 May 19;15:150. doi: 10.1186/1471-2105-15-150 (PMC4229970; doi:10.1186/1471-2105-15-150)
Supplement: Additional file 2 — Java code of ml2db. Additional file ml2db_code.tar.gz contains the Java source code to run the multi-label machine learning experiments and save the results to database. The code’s Javadoc is included. [file 1471-2105-15-150-S2.zip › additional file 2/ml2db/ecmulan/doc/uk/ac/ed/inf/mulanxml/class-use/XmlCreator.html]

Uses of Class uk.ac.ed.inf.mulanxml.XmlCreator


JavaScript is disabled on your browser.


- Overview
- Package
- Class
- Use
- Tree
- Deprecated
- Index
- Help

- Prev
- Next

- Frames
- No Frames

- All Classes

## Uses of Class uk.ac.ed.inf.mulanxml.XmlCreator

- Packages that use XmlCreator

  | Package | Description |
  |  |  |
  | --- | --- |
  | uk.ac.ed.inf.mulanxml |  |
  | uk.ac.ed.inf.mulanxml.ec |  |
- - ### Uses of XmlCreator in uk.ac.ed.inf.mulanxml

    Methods in uk.ac.ed.inf.mulanxml that return XmlCreator

    | Modifier and Type | Method and Description |
    |  |  |
    | --- | --- |
    | `static XmlCreator` | XmlCreatorTest.`getXmlCreator()` Test method for `XmlCreator(uk.ac.ed.inf.mulanxml.XmlCreatorManager, java.util.TreeSet)`. |
    | `XmlCreator` | XmlCreatorManager.`getXmlCreator()` |
  - ### Uses of XmlCreator in uk.ac.ed.inf.mulanxml.ec

    Subclasses of XmlCreator in uk.ac.ed.inf.mulanxml.ec

    | Modifier and Type | Class and Description |
    |  |  |
    | --- | --- |
    | `class` | `EcFullXmlCreator` Creates a full XML hierarchical representation of Enzyme Commission numbers in Mulan format. |
    | `class` | `EcMulanXmlCreator` Creates a full XML hierarchical representation of Enzyme Commission numbers in Mulan format. |

- Overview
- Package
- Class
- Use
- Tree
- Deprecated
- Index
- Help

- Prev
- Next

- Frames
- No Frames

- All Classes
