# Supplementary material for: From sequence to enzyme mechanism using multi-label machine learning
Source: BMC Bioinformatics. 2014 May 19;15:150. doi: 10.1186/1471-2105-15-150 (PMC4229970; doi:10.1186/1471-2105-15-150)
Supplement: Additional file 2 — Java code of ml2db. Additional file ml2db_code.tar.gz contains the Java source code to run the multi-label machine learning experiments and save the results to database. The code’s Javadoc is included. [file 1471-2105-15-150-S2.zip › additional file 2/ml2db/ecmulan/doc/uk/ac/ed/inf/mulanxml/class-use/XmlCreatorManager.html]

Uses of Class uk.ac.ed.inf.mulanxml.XmlCreatorManager


JavaScript is disabled on your browser.


- Overview
- Package
- Class
- Use
- Tree
- Deprecated
- Index
- Help

- Prev
- Next

- Frames
- No Frames

- All Classes

## Uses of Class uk.ac.ed.inf.mulanxml.XmlCreatorManager

- Packages that use XmlCreatorManager

  | Package | Description |
  |  |  |
  | --- | --- |
  | uk.ac.ed.inf.mulanxml |  |
  | uk.ac.ed.inf.mulanxml.ec |  |
- - ### Uses of XmlCreatorManager in uk.ac.ed.inf.mulanxml

    Methods in uk.ac.ed.inf.mulanxml that return XmlCreatorManager

    | Modifier and Type | Method and Description |
    |  |  |
    | --- | --- |
    | `XmlCreatorManager` | XmlCreator.`getManager()` |
    | `static XmlCreatorManager` | XmlCreatorManagerTest.`getXmlCreatorManager1()` Test method for `XmlCreatorManager(java.lang.String, java.lang.String)`. |

    Constructors in uk.ac.ed.inf.mulanxml with parameters of type XmlCreatorManager

    | Constructor and Description |
    |  |
    | --- |
    | `XmlCreator(XmlCreatorManager manager, java.util.TreeSet<java.lang.String> labels)` Get labels from database |
  - ### Uses of XmlCreatorManager in uk.ac.ed.inf.mulanxml.ec

    Constructors in uk.ac.ed.inf.mulanxml.ec with parameters of type XmlCreatorManager

    | Constructor and Description |
    |  |
    | --- |
    | `EcFullXmlCreator(XmlCreatorManager manager, java.util.TreeSet<java.lang.String> ecNumberStrings)` Get EC numbers from database |
    | `EcMulanXmlCreator(XmlCreatorManager manager, java.util.TreeSet<java.lang.String> ecNumberStrings)` |

- Overview
- Package
- Class
- Use
- Tree
- Deprecated
- Index
- Help

- Prev
- Next

- Frames
- No Frames

- All Classes
