# Supplementary material for: From sequence to enzyme mechanism using multi-label machine learning
Source: BMC Bioinformatics. 2014 May 19;15:150. doi: 10.1186/1471-2105-15-150 (PMC4229970; doi:10.1186/1471-2105-15-150)
Supplement: Additional file 2 — Java code of ml2db. Additional file ml2db_code.tar.gz contains the Java source code to run the multi-label machine learning experiments and save the results to database. The code’s Javadoc is included. [file 1471-2105-15-150-S2.zip › additional file 2/ml2db/ecmulan/doc/uk/ac/ed/inf/mulanxml/ec/EcDbWriter.html]

EcDbWriter


JavaScript is disabled on your browser.


- Overview
- Package
- Class
- Use
- Tree
- Deprecated
- Index
- Help

- Prev Class
- Next Class

- Frames
- No Frames

- All Classes

- Summary:
- Nested |
- Field |
- Constr |
- Method

- Detail:
- Field |
- Constr |
- Method


uk.ac.ed.inf.mulanxml.ec

## Class EcDbWriter

- java.lang.Object
- - uk.ac.standrews.utils.main.database.Manager
  - - uk.ac.standrews.utils.main.database.DbManager
    - - uk.ac.ed.inf.mulanxml.ec.EcDbWriter

- All Implemented Interfaces:
  :   java.io.Serializable

  ---

    

  ```
  public class EcDbWriter
  extends uk.ac.standrews.utils.main.database.DbManager
  ```

  Given a database and a list of Enzyme commission numbers, writes a 2 columns
  table containing: in column 1: the Ec number, in column 2: all the ancestors
  of that EC number, including itself. (It also adds the EC number ancestors to
  column 1, so that their subclasses are included in column 2 too)

  Version:
  :   3 Jun 2010

  Author:
  :   Luna De Ferrari luna.deferrari-at-ed.ac.uk

  See Also:
  :   Serialized Form

- - ### Field Summary

    Fields

    | Modifier and Type | Field and Description |
    | `static java.lang.String` | `ANCESTOR_FIELD_NAME` the name of the field to contain the ec number's ancestors |
    | `static java.lang.String` | `EC_DATA_TYPE` the sql data type for ec numbers |
    | `static java.lang.String` | `EC_FIELD_NAME` the name of the field to contain the ec number |

    - ### Fields inherited from class uk.ac.standrews.utils.main.database.DbManager

      `m_dbWriter`
  - ### Constructor Summary

    Constructors

    | Constructor and Description |
    | `EcDbWriter(uk.ac.standrews.utils.main.database.DbManager manager, java.lang.String getEcSqlQuery)` Reads ec numbers from database and writes them and their ancestors to a new table |
  - ### Method Summary

    Methods

    | Modifier and Type | Method and Description |
    | `EcTable` | `getEcTable()` |
    | `static void` | `main(java.lang.String[] args)` Main for recreating table |
    | `void` | `writeEcAndAncestorsToTable()` |

    - ### Methods inherited from class uk.ac.standrews.utils.main.database.DbManager

      `closeConnection, connectionIsValid, getDatabaseName, getDbConnection, getDbCreator, getDbReader, getDbWriter, refreshConnection, runAnalyzeTable`
    - ### Methods inherited from class java.lang.Object

      `equals, getClass, hashCode, notify, notifyAll, toString, wait, wait, wait`

- - ### Field Detail


    - #### ANCESTOR\_FIELD\_NAME

      ```
      public static final java.lang.String ANCESTOR_FIELD_NAME
      ```

      the name of the field to contain the ec number's ancestors

      See Also:
      :   Constant Field Values


    - #### EC\_DATA\_TYPE

      ```
      public static final java.lang.String EC_DATA_TYPE
      ```

      the sql data type for ec numbers

      See Also:
      :   Constant Field Values


    - #### EC\_FIELD\_NAME

      ```
      public static final java.lang.String EC_FIELD_NAME
      ```

      the name of the field to contain the ec number

      See Also:
      :   Constant Field Values
  - ### Constructor Detail


    - #### EcDbWriter

      ```
      public EcDbWriter(uk.ac.standrews.utils.main.database.DbManager manager,
                java.lang.String getEcSqlQuery)
      ```

      Reads ec numbers from database and writes them and their ancestors to a
      new table
  - ### Method Detail


    - #### getEcTable

      ```
      public EcTable getEcTable()
      ```


    - #### writeEcAndAncestorsToTable

      ```
      public void writeEcAndAncestorsToTable()
      ```


    - #### main

      ```
      public static void main(java.lang.String[] args)
                       throws java.sql.SQLException
      ```

      Main for recreating table

      Parameters:
      :   `args` -

      Throws:
      :   `java.sql.SQLException`


- Overview
- Package
- Class
- Use
- Tree
- Deprecated
- Index
- Help

- Prev Class
- Next Class

- Frames
- No Frames

- All Classes

- Summary:
- Nested |
- Field |
- Constr |
- Method

- Detail:
- Field |
- Constr |
- Method
