# Supplementary material for: From sequence to enzyme mechanism using multi-label machine learning
Source: BMC Bioinformatics. 2014 May 19;15:150. doi: 10.1186/1471-2105-15-150 (PMC4229970; doi:10.1186/1471-2105-15-150)
Supplement: Additional file 2 — Java code of ml2db. Additional file ml2db_code.tar.gz contains the Java source code to run the multi-label machine learning experiments and save the results to database. The code’s Javadoc is included. [file 1471-2105-15-150-S2.zip › additional file 2/ml2db/ecmulan/doc/uk/ac/ed/inf/mulanxml/ec/EcFullXmlCreator.html]

EcFullXmlCreator


JavaScript is disabled on your browser.


- Overview
- Package
- Class
- Use
- Tree
- Deprecated
- Index
- Help

- Prev Class
- Next Class

- Frames
- No Frames

- All Classes

- Summary:
- Nested |
- Field |
- Constr |
- Method

- Detail:
- Field |
- Constr |
- Method


uk.ac.ed.inf.mulanxml.ec

## Class EcFullXmlCreator

- java.lang.Object
- - uk.ac.ed.inf.mulanxml.XmlCreator
  - - uk.ac.ed.inf.mulanxml.ec.EcFullXmlCreator

- Direct Known Subclasses:
  :   EcMulanXmlCreator

  ---

    

  ```
  public class EcFullXmlCreator
  extends XmlCreator
  ```

  Creates a full XML hierarchical representation of Enzyme Commission numbers
  in Mulan format. Note: it creates also EC numbers that might not be present
  in the original list, but are ancestors of the given numbers. The ancestor of
  all EC numbers is the pseudo-EC numbers "-.-.-.-" (4 dashes)

  Version:
  :   4 Nov 2010

  Author:
  :   Luna De Ferrari luna.deferrari-at-ed.ac.uk

- - ### Constructor Summary

    Constructors

    | Constructor and Description |
    | `EcFullXmlCreator(XmlCreatorManager manager, java.util.TreeSet<java.lang.String> ecNumberStrings)` Get EC numbers from database |
  - ### Method Summary

    Methods

    | Modifier and Type | Method and Description |
    | `java.lang.String` | `createXmlString()` Generate an XML representation of the Enzyme Commission number hierarchy in Mulan format. |
    | `java.util.TreeSet<EcNumber>` | `getEcNumbers()` |
    | `static void` | `main(java.lang.String[] args)` |

    - ### Methods inherited from class uk.ac.ed.inf.mulanxml.XmlCreator

      `getLabels, getManager, getXml, getXmlString, saveToFile`
    - ### Methods inherited from class java.lang.Object

      `equals, getClass, hashCode, notify, notifyAll, toString, wait, wait, wait`

- - ### Constructor Detail


    - #### EcFullXmlCreator

      ```
      public EcFullXmlCreator(XmlCreatorManager manager,
                      java.util.TreeSet<java.lang.String> ecNumberStrings)
      ```

      Get EC numbers from database

      Parameters:
      :   `dbConnPropsPath` -
  - ### Method Detail


    - #### createXmlString

      ```
      public java.lang.String createXmlString()
      ```

      Generate an XML representation of the Enzyme Commission number hierarchy
      in Mulan format.

      **Overrides:**
      :   `createXmlString` in class `XmlCreator`

      Returns:
      :   an XML representation of the Enzyme Commission number hierarchy
          in Mulan format.


    - #### getEcNumbers

      ```
      public java.util.TreeSet<EcNumber> getEcNumbers()
      ```


    - #### main

      ```
      public static void main(java.lang.String[] args)
      ```


- Overview
- Package
- Class
- Use
- Tree
- Deprecated
- Index
- Help

- Prev Class
- Next Class

- Frames
- No Frames

- All Classes

- Summary:
- Nested |
- Field |
- Constr |
- Method

- Detail:
- Field |
- Constr |
- Method
