# Supplementary material for: From sequence to enzyme mechanism using multi-label machine learning
Source: BMC Bioinformatics. 2014 May 19;15:150. doi: 10.1186/1471-2105-15-150 (PMC4229970; doi:10.1186/1471-2105-15-150)
Supplement: Additional file 2 — Java code of ml2db. Additional file ml2db_code.tar.gz contains the Java source code to run the multi-label machine learning experiments and save the results to database. The code’s Javadoc is included. [file 1471-2105-15-150-S2.zip › additional file 2/ml2db/ecmulan/doc/uk/ac/ed/inf/mulanxml/ec/EcMulanXmlCreator.html]

EcMulanXmlCreator


JavaScript is disabled on your browser.


- Overview
- Package
- Class
- Use
- Tree
- Deprecated
- Index
- Help

- Prev Class
- Next Class

- Frames
- No Frames

- All Classes

- Summary:
- Nested |
- Field |
- Constr |
- Method

- Detail:
- Field |
- Constr |
- Method


uk.ac.ed.inf.mulanxml.ec

## Class EcMulanXmlCreator

- java.lang.Object
- - uk.ac.ed.inf.mulanxml.XmlCreator
  - - uk.ac.ed.inf.mulanxml.ec.EcFullXmlCreator
    - - uk.ac.ed.inf.mulanxml.ec.EcMulanXmlCreator

- ---

    

  ```
  public class EcMulanXmlCreator
  extends EcFullXmlCreator
  ```

  Creates a full XML hierarchical representation of Enzyme Commission numbers
  in Mulan format.

  Version:
  :   2 Jun 2010

  Author:
  :   Luna De Ferrari luna.deferrari-at-ed.ac.uk

- - ### Constructor Summary

    Constructors

    | Constructor and Description |
    | `EcMulanXmlCreator(XmlCreatorManager manager, java.util.TreeSet<java.lang.String> ecNumberStrings)` |
  - ### Method Summary

    Methods

    | Modifier and Type | Method and Description |
    | `java.lang.String` | `createXmlString()` Generate an XML representation of the Enzyme Commission number hierarchy in Mulan format. |
    | `static void` | `main(java.lang.String[] args)` |

    - ### Methods inherited from class uk.ac.ed.inf.mulanxml.ec.EcFullXmlCreator

      `getEcNumbers`
    - ### Methods inherited from class uk.ac.ed.inf.mulanxml.XmlCreator

      `getLabels, getManager, getXml, getXmlString, saveToFile`
    - ### Methods inherited from class java.lang.Object

      `equals, getClass, hashCode, notify, notifyAll, toString, wait, wait, wait`

- - ### Constructor Detail


    - #### EcMulanXmlCreator

      ```
      public EcMulanXmlCreator(XmlCreatorManager manager,
                       java.util.TreeSet<java.lang.String> ecNumberStrings)
      ```
  - ### Method Detail


    - #### createXmlString

      ```
      public java.lang.String createXmlString()
      ```

      **Description copied from class: `EcFullXmlCreator`**

      Generate an XML representation of the Enzyme Commission number hierarchy
      in Mulan format.

      **Overrides:**
      :   `createXmlString` in class `EcFullXmlCreator`

      Returns:
      :   an XML representation of the Enzyme Commission number hierarchy
          in Mulan format.


    - #### main

      ```
      public static void main(java.lang.String[] args)
      ```


- Overview
- Package
- Class
- Use
- Tree
- Deprecated
- Index
- Help

- Prev Class
- Next Class

- Frames
- No Frames

- All Classes

- Summary:
- Nested |
- Field |
- Constr |
- Method

- Detail:
- Field |
- Constr |
- Method
