# Supplementary material for: From sequence to enzyme mechanism using multi-label machine learning
Source: BMC Bioinformatics. 2014 May 19;15:150. doi: 10.1186/1471-2105-15-150 (PMC4229970; doi:10.1186/1471-2105-15-150)
Supplement: Additional file 2 — Java code of ml2db. Additional file ml2db_code.tar.gz contains the Java source code to run the multi-label machine learning experiments and save the results to database. The code’s Javadoc is included. [file 1471-2105-15-150-S2.zip › additional file 2/ml2db/ecmulan/doc/uk/ac/ed/inf/mulanxml/ec/EcNumber.html]

EcNumber


JavaScript is disabled on your browser.


- Overview
- Package
- Class
- Use
- Tree
- Deprecated
- Index
- Help

- Prev Class
- Next Class

- Frames
- No Frames

- All Classes

- Summary:
- Nested |
- Field |
- Constr |
- Method

- Detail:
- Field |
- Constr |
- Method


uk.ac.ed.inf.mulanxml.ec

## Class EcNumber

- java.lang.Object
- - uk.ac.ed.inf.mulanxml.ec.EcNumber

- All Implemented Interfaces:
  :   java.lang.Comparable<EcNumber>

  ---

    

  ```
  public class EcNumber
  extends java.lang.Object
  implements java.lang.Comparable<EcNumber>
  ```

  A class representing an Enzyme Commission (EC) number. The enzyme commission
  has set a standard nomenclature (4 level deep numerical hierarchy) of
  enzymatic reactions. More at: http://www.chem.qmul.ac.uk/iubmb/enzyme/
  An example of complete EC number is: 1.2.3.4 Incomplete EC numbers are, for
  example: 1.-.-.-, 1.2.-.-, 1.2.3.-

  Version:
  :   11 Nov 2010

  Author:
  :   Luna De Ferrari luna.deferrari-at-ed.ac.uk

- - ### Constructor Summary

    Constructors

    | Constructor and Description |
    | `EcNumber(java.lang.String ec)` |
  - ### Method Summary

    Methods

    | Modifier and Type | Method and Description |
    | `int` | `compareTo(EcNumber otherEc)` Order by EC string |
    | `java.lang.String[]` | `getAncestorsStrings()` Get a complete hierarchy of ancestor for the ec number. |
    | `java.lang.String[]` | `getBlocks()` |
    | `java.lang.String` | `getEcString()` |
    | `static java.lang.String` | `getEcStringFromBlocks(java.lang.String[] blocks)` |
    | `int` | `getHierarchyLevel()` |
    | `EcNumber` | `getParent()` |
    | `java.lang.String` | `getParentString()` |
    | `boolean` | `isComplete()` |
    | `boolean` | `isParent(EcNumber possibleChild)` Returns true if the node given is a direct child of this node: e.g: 1.2.3.4 is direct child of 1.2.3.-, but it is not direct child of 1.2.-.- |

    - ### Methods inherited from class java.lang.Object

      `equals, getClass, hashCode, notify, notifyAll, toString, wait, wait, wait`

- - ### Constructor Detail


    - #### EcNumber

      ```
      public EcNumber(java.lang.String ec)
      ```
  - ### Method Detail


    - #### compareTo

      ```
      public int compareTo(EcNumber otherEc)
      ```

      Order by EC string

      **Specified by:**
      :   `compareTo` in interface `java.lang.Comparable<EcNumber>`


    - #### getAncestorsStrings

      ```
      public java.lang.String[] getAncestorsStrings()
      ```

      Get a complete hierarchy of ancestor for the ec number. For EC 1.2.3.4 it
      is [1.-.-.-, 1.2.---, 1.2.3.-, 1.2.3.4] For EC 1.2.-.- it is [1.-.-.-,
      1.2.---, null, null]


    - #### getBlocks

      ```
      public java.lang.String[] getBlocks()
      ```


    - #### getEcString

      ```
      public java.lang.String getEcString()
      ```


    - #### getHierarchyLevel

      ```
      public int getHierarchyLevel()
      ```


    - #### getParent

      ```
      public EcNumber getParent()
      ```


    - #### getParentString

      ```
      public java.lang.String getParentString()
      ```


    - #### isComplete

      ```
      public boolean isComplete()
      ```


    - #### isParent

      ```
      public boolean isParent(EcNumber possibleChild)
      ```

      Returns true if the node given is a direct child of this node: e.g:
      1.2.3.4 is direct child of 1.2.3.-, but it is not direct child of 1.2.-.-

      Parameters:
      :   `possibleChild` -

      Returns:
      :   true if the given node is a direct child of this node


    - #### getEcStringFromBlocks

      ```
      public static java.lang.String getEcStringFromBlocks(java.lang.String[] blocks)
      ```


- Overview
- Package
- Class
- Use
- Tree
- Deprecated
- Index
- Help

- Prev Class
- Next Class

- Frames
- No Frames

- All Classes

- Summary:
- Nested |
- Field |
- Constr |
- Method

- Detail:
- Field |
- Constr |
- Method
