# Supplementary material for: From sequence to enzyme mechanism using multi-label machine learning
Source: BMC Bioinformatics. 2014 May 19;15:150. doi: 10.1186/1471-2105-15-150 (PMC4229970; doi:10.1186/1471-2105-15-150)
Supplement: Additional file 2 — Java code of ml2db. Additional file ml2db_code.tar.gz contains the Java source code to run the multi-label machine learning experiments and save the results to database. The code’s Javadoc is included. [file 1471-2105-15-150-S2.zip › additional file 2/ml2db/ecmulan/doc/uk/ac/ed/inf/mulanxml/ec/EcNumberGenerator.html]

EcNumberGenerator


JavaScript is disabled on your browser.


- Overview
- Package
- Class
- Use
- Tree
- Deprecated
- Index
- Help

- Prev Class
- Next Class

- Frames
- No Frames

- All Classes

- Summary:
- Nested |
- Field |
- Constr |
- Method

- Detail:
- Field |
- Constr |
- Method


uk.ac.ed.inf.mulanxml.ec

## Class EcNumberGenerator

- java.lang.Object
- - uk.ac.ed.inf.mulanxml.ec.EcNumberGenerator

- ---

    

  ```
  public class EcNumberGenerator
  extends java.lang.Object
  ```

  Given a string such as 1.2.3.4 or 1.2.-.-.- checks if it is a valid Enzyme
  commission number and generates the Java EcNumber object (including its
  parent EC numbers)

  Version:
  :   8 Jun 2010

  Author:
  :   Luna De Ferrari luna.deferrari-at-ed.ac.uk

- - ### Field Summary

    Fields

    | Modifier and Type | Field and Description |
    | `static java.lang.String` | `DASH` the substitute for an unknown digit blocks (a dash) |
    | `static java.lang.String` | `DOT` the separator for the digit blocks (dot) |
    | `static int` | `FULL_HIERARCHY_LENGHT` the number of levels in the EC number parent-child hierarchy (if -.-.-.- is included = 5, traditional ec hierarchy = 4 ) |
    | `static java.lang.String` | `MAX_LEVEL1_CLASS` maximum value for level 1 class: currently 6. |
    | `static java.lang.String` | `MAX_LEVEL1_REGEXP` regexp from 1 to 6 |
    | `static java.lang.String` | `MAX_LEVEL2_CLASS` maximum value for level 2 class: 99 in this implementation. |
    | `static java.lang.String` | `MAX_LEVEL2_REGEXP` regexp from 0 to 99 |
    | `static java.lang.String` | `MAX_LEVEL3_CLASS` maximum value for level 3 class: 99 in this implementation. |
    | `static java.lang.String` | `MAX_LEVEL3_REGEXP` regexp from 0 to 99 |
    | `static java.lang.String` | `MAX_LEVEL4_CLASS` maximum value for level 4 class: 999 in this implementation. |
    | `static java.lang.String` | `MAX_LEVEL4_REGEXP` regexp from 0 to 999, potentially prefixed with "n" in uniprot ("new" ec numbers) |
  - ### Constructor Summary

    Constructors

    | Constructor and Description |
    | `EcNumberGenerator(java.lang.String ec)` |
  - ### Method Summary

    Methods

    | Modifier and Type | Method and Description |
    | `static boolean` | `couldBeDashedEc(java.lang.String string)` Checks whether the string could contain a validly formatted ec number with dashes (1.-.-.-, 1.2.3.- etc., but not 1.-.3.4) |
    | `static boolean` | `couldBeEc(java.lang.String string)` Checks if the string could contain a validly formatted EC number. |
    | `static EcNumber` | `generateEcNumber(java.lang.String ec)` |
    | `static boolean` | `isValidEcBlock(java.lang.String block)` True if the string contains a dash '-' or a positive integer or a positive integer preceded by 'n' (new uniprot ec numbers) |

    - ### Methods inherited from class java.lang.Object

      `equals, getClass, hashCode, notify, notifyAll, toString, wait, wait, wait`

- - ### Field Detail


    - #### DASH

      ```
      public static final java.lang.String DASH
      ```

      the substitute for an unknown digit blocks (a dash)

      See Also:
      :   Constant Field Values


    - #### DOT

      ```
      public static final java.lang.String DOT
      ```

      the separator for the digit blocks (dot)

      See Also:
      :   Constant Field Values


    - #### MAX\_LEVEL1\_CLASS

      ```
      public static final java.lang.String MAX_LEVEL1_CLASS
      ```

      maximum value for level 1 class: currently 6. No EC number can start with
      7.-.-.-

      See Also:
      :   Constant Field Values


    - #### MAX\_LEVEL1\_REGEXP

      ```
      public static final java.lang.String MAX_LEVEL1_REGEXP
      ```

      regexp from 1 to 6

      See Also:
      :   Constant Field Values


    - #### MAX\_LEVEL2\_CLASS

      ```
      public static final java.lang.String MAX_LEVEL2_CLASS
      ```

      maximum value for level 2 class: 99 in this implementation. No EC number
      can have 1.100.-.-

      See Also:
      :   Constant Field Values


    - #### MAX\_LEVEL2\_REGEXP

      ```
      public static final java.lang.String MAX_LEVEL2_REGEXP
      ```

      regexp from 0 to 99

      See Also:
      :   Constant Field Values


    - #### MAX\_LEVEL3\_CLASS

      ```
      public static final java.lang.String MAX_LEVEL3_CLASS
      ```

      maximum value for level 3 class: 99 in this implementation. No EC number
      can have 1.1.100.-

      See Also:
      :   Constant Field Values


    - #### MAX\_LEVEL3\_REGEXP

      ```
      public static final java.lang.String MAX_LEVEL3_REGEXP
      ```

      regexp from 0 to 99

      See Also:
      :   Constant Field Values


    - #### MAX\_LEVEL4\_CLASS

      ```
      public static final java.lang.String MAX_LEVEL4_CLASS
      ```

      maximum value for level 4 class: 999 in this implementation.

      See Also:
      :   Constant Field Values


    - #### MAX\_LEVEL4\_REGEXP

      ```
      public static final java.lang.String MAX_LEVEL4_REGEXP
      ```

      regexp from 0 to 999, potentially prefixed with "n" in uniprot ("new" ec numbers)

      See Also:
      :   Constant Field Values


    - #### FULL\_HIERARCHY\_LENGHT

      ```
      public static final int FULL_HIERARCHY_LENGHT
      ```

      the number of levels in the EC number parent-child hierarchy (if -.-.-.-
      is included = 5, traditional ec hierarchy = 4 )

      See Also:
      :   Constant Field Values
  - ### Constructor Detail


    - #### EcNumberGenerator

      ```
      public EcNumberGenerator(java.lang.String ec)
      ```

      Parameters:
      :   `ec` - the ec number string
  - ### Method Detail


    - #### couldBeDashedEc

      ```
      public static boolean couldBeDashedEc(java.lang.String string)
      ```

      Checks whether the string could contain a validly formatted ec number
      with dashes (1.-.-.-, 1.2.3.- etc., but not 1.-.3.4)

      Parameters:
      :   `string` - the ec string

      Returns:
      :   true if the format is valid


    - #### couldBeEc

      ```
      public static boolean couldBeEc(java.lang.String string)
      ```

      Checks if the string could contain a validly formatted EC number.

      Parameters:
      :   `string` - the ec number string

      Returns:
      :   true if the format is compatible with an EC number


    - #### generateEcNumber

      ```
      public static EcNumber generateEcNumber(java.lang.String ec)
      ```


    - #### isValidEcBlock

      ```
      public static boolean isValidEcBlock(java.lang.String block)
      ```

      True if the string contains a dash '-' or a positive integer or a
      positive integer preceded by 'n' (new uniprot ec numbers)


- Overview
- Package
- Class
- Use
- Tree
- Deprecated
- Index
- Help

- Prev Class
- Next Class

- Frames
- No Frames

- All Classes

- Summary:
- Nested |
- Field |
- Constr |
- Method

- Detail:
- Field |
- Constr |
- Method
