# Supplementary material for: From sequence to enzyme mechanism using multi-label machine learning
Source: BMC Bioinformatics. 2014 May 19;15:150. doi: 10.1186/1471-2105-15-150 (PMC4229970; doi:10.1186/1471-2105-15-150)
Supplement: Additional file 2 — Java code of ml2db. Additional file ml2db_code.tar.gz contains the Java source code to run the multi-label machine learning experiments and save the results to database. The code’s Javadoc is included. [file 1471-2105-15-150-S2.zip › additional file 2/ml2db/ecmulan/doc/uk/ac/ed/inf/mulanxml/ec/EcTable.html]

EcTable


JavaScript is disabled on your browser.


- Overview
- Package
- Class
- Use
- Tree
- Deprecated
- Index
- Help

- Prev Class
- Next Class

- Frames
- No Frames

- All Classes

- Summary:
- Nested |
- Field |
- Constr |
- Method

- Detail:
- Field |
- Constr |
- Method


uk.ac.ed.inf.mulanxml.ec

## Class EcTable

- java.lang.Object
- - uk.ac.standrews.utils.main.database.Table
  - - uk.ac.ed.inf.mulanxml.ec.EcTable

- ---

    

  ```
  public class EcTable
  extends uk.ac.standrews.utils.main.database.Table
  ```

  The table to store Enzyme commission numbers and their ancestors.

  Version:
  :   27 Mar 2013

  Author:
  :   ldf

- - ### Field Summary

    Fields

    | Modifier and Type | Field and Description |
    | `static java.lang.String` | `EC_TABLE_NAME` the name of the table for ec and ancestors |

    - ### Fields inherited from class uk.ac.standrews.utils.main.database.Table

      `m_writer`
  - ### Constructor Summary

    Constructors

    | Constructor and Description |
    | `EcTable(uk.ac.standrews.utils.main.database.DbManager dbManager)` |
  - ### Method Summary

    Methods

    | Modifier and Type | Method and Description |
    | `static java.util.Vector<uk.ac.standrews.utils.main.database.TableColumn>` | `getColumnDefinitions()` Creates the table for the ec numbers and ancestors |
    | `int` | `saveRow(java.lang.String ec, java.lang.String ancestor)` Write one ec number + ancestor couple to table |

    - ### Methods inherited from class uk.ac.standrews.utils.main.database.Table

      `getColumnNames, getColumns, getDbManager, getTableCreator, getTableName, getTableReader, getTableRow, getTableWriter, hasKey, initialise, initialise, name, saveRow`
    - ### Methods inherited from class java.lang.Object

      `equals, getClass, hashCode, notify, notifyAll, toString, wait, wait, wait`

- - ### Field Detail


    - #### EC\_TABLE\_NAME

      ```
      public static final java.lang.String EC_TABLE_NAME
      ```

      the name of the table for ec and ancestors

      See Also:
      :   Constant Field Values
  - ### Constructor Detail


    - #### EcTable

      ```
      public EcTable(uk.ac.standrews.utils.main.database.DbManager dbManager)
      ```

      Parameters:
      :   `m_tableName` -
      :   `m_columns` -
      :   `m_dbManager` -
  - ### Method Detail


    - #### getColumnDefinitions

      ```
      public static java.util.Vector<uk.ac.standrews.utils.main.database.TableColumn> getColumnDefinitions()
      ```

      Creates the table for the ec numbers and ancestors

      Returns:
      :   the table


    - #### saveRow

      ```
      public int saveRow(java.lang.String ec,
                java.lang.String ancestor)
      ```

      Write one ec number + ancestor couple to table

      Parameters:
      :   `ec` - the enzyme commission number
      :   `ancestor` - the ec number ancestor


- Overview
- Package
- Class
- Use
- Tree
- Deprecated
- Index
- Help

- Prev Class
- Next Class

- Frames
- No Frames

- All Classes

- Summary:
- Nested |
- Field |
- Constr |
- Method

- Detail:
- Field |
- Constr |
- Method
