# Supplementary material for: From sequence to enzyme mechanism using multi-label machine learning
Source: BMC Bioinformatics. 2014 May 19;15:150. doi: 10.1186/1471-2105-15-150 (PMC4229970; doi:10.1186/1471-2105-15-150)
Supplement: Additional file 2 — Java code of ml2db. Additional file ml2db_code.tar.gz contains the Java source code to run the multi-label machine learning experiments and save the results to database. The code’s Javadoc is included. [file 1471-2105-15-150-S2.zip › additional file 2/ml2db/ecmulan/doc/uk/ac/ed/inf/mulanxml/ec/package-use.html]

Uses of Package uk.ac.ed.inf.mulanxml.ec


JavaScript is disabled on your browser.


- Overview
- Package
- Class
- Use
- Tree
- Deprecated
- Index
- Help

- Prev
- Next

- Frames
- No Frames

- All Classes

# Uses of Package uk.ac.ed.inf.mulanxml.ec

- Packages that use uk.ac.ed.inf.mulanxml.ec

  | Package | Description |
  |  |  |
  | --- | --- |
  | uk.ac.ed.inf.mulanxml.ec |  |
  | uk.ac.ed.inf.mulanxml.test.ec |  |
- Classes in uk.ac.ed.inf.mulanxml.ec used by uk.ac.ed.inf.mulanxml.ec

  | Class and Description |
  |  |
  | --- |
  | EcFullXmlCreator Creates a full XML hierarchical representation of Enzyme Commission numbers in Mulan format. |
  | EcNumber A class representing an Enzyme Commission (EC) number. |
  | EcTable The table to store Enzyme commission numbers and their ancestors. |
- Classes in uk.ac.ed.inf.mulanxml.ec used by uk.ac.ed.inf.mulanxml.test.ec

  | Class and Description |
  |  |
  | --- |
  | EcDbWriter Given a database and a list of Enzyme commission numbers, writes a 2 columns table containing: in column 1: the Ec number, in column 2: all the ancestors of that EC number, including itself. |
  | EcFullXmlCreator Creates a full XML hierarchical representation of Enzyme Commission numbers in Mulan format. |
  | EcMulanXmlCreator Creates a full XML hierarchical representation of Enzyme Commission numbers in Mulan format. |
  | EcNumber A class representing an Enzyme Commission (EC) number. |

- Overview
- Package
- Class
- Use
- Tree
- Deprecated
- Index
- Help

- Prev
- Next

- Frames
- No Frames

- All Classes
