# Supplementary material for: From sequence to enzyme mechanism using multi-label machine learning
Source: BMC Bioinformatics. 2014 May 19;15:150. doi: 10.1186/1471-2105-15-150 (PMC4229970; doi:10.1186/1471-2105-15-150)
Supplement: Additional file 2 — Java code of ml2db. Additional file ml2db_code.tar.gz contains the Java source code to run the multi-label machine learning experiments and save the results to database. The code’s Javadoc is included. [file 1471-2105-15-150-S2.zip › additional file 2/ml2db/ecmulan/doc/uk/ac/ed/inf/mulanxml/LocalDbReader.html]

LocalDbReader


JavaScript is disabled on your browser.


- Overview
- Package
- Class
- Use
- Tree
- Deprecated
- Index
- Help

- Prev Class
- Next Class

- Frames
- No Frames

- All Classes

- Summary:
- Nested |
- Field |
- Constr |
- Method

- Detail:
- Field |
- Constr |
- Method


uk.ac.ed.inf.mulanxml

## Class LocalDbReader

- java.lang.Object
- - uk.ac.standrews.utils.main.database.DbManaged
  - - uk.ac.standrews.utils.main.database.DbReader
    - - uk.ac.ed.inf.mulanxml.LocalDbReader

- ---

    

  ```
  public class LocalDbReader
  extends uk.ac.standrews.utils.main.database.DbReader
  ```

  Reads the full list of Enzyme Commission numbers from database

  Version:
  :   5 May 2010

  Author:
  :   Luna De Ferrari luna.deferrari-at-ed.ac.uk

- - ### Field Summary

    Fields

    | Modifier and Type | Field and Description |
    | `java.lang.String` | `m_ecSqlQuery` |
  - ### Constructor Summary

    Constructors

    | Constructor and Description |
    | `LocalDbReader(uk.ac.standrews.utils.main.database.DbManager dbManager, java.lang.String ecSqlQuery)` |
  - ### Method Summary

    Methods

    | Modifier and Type | Method and Description |
    | `java.lang.String` | `getEcSqlQuery()` |
    | `java.util.TreeSet<java.lang.String>` | `getLabelList()` |

    - ### Methods inherited from class uk.ac.standrews.utils.main.database.DbReader

      `executeQuery, getColumnsValues, getColumnValues, getDbMetaData, getListOfDbTables, getMap, getMap, getStatement, queryResultsColumnToCollection, queryResultsColumnToSet, queryResultsColumnToVector, tableExistsInDb`
    - ### Methods inherited from class uk.ac.standrews.utils.main.database.DbManaged

      `getDbConnection, getDbManager, getDbReader`
    - ### Methods inherited from class java.lang.Object

      `equals, getClass, hashCode, notify, notifyAll, toString, wait, wait, wait`

- - ### Field Detail


    - #### m\_ecSqlQuery

      ```
      public java.lang.String m_ecSqlQuery
      ```
  - ### Constructor Detail


    - #### LocalDbReader

      ```
      public LocalDbReader(uk.ac.standrews.utils.main.database.DbManager dbManager,
                   java.lang.String ecSqlQuery)
      ```
  - ### Method Detail


    - #### getLabelList

      ```
      public java.util.TreeSet<java.lang.String> getLabelList()
      ```


    - #### getEcSqlQuery

      ```
      public java.lang.String getEcSqlQuery()
      ```


- Overview
- Package
- Class
- Use
- Tree
- Deprecated
- Index
- Help

- Prev Class
- Next Class

- Frames
- No Frames

- All Classes

- Summary:
- Nested |
- Field |
- Constr |
- Method

- Detail:
- Field |
- Constr |
- Method
