# Supplementary material for: From sequence to enzyme mechanism using multi-label machine learning
Source: BMC Bioinformatics. 2014 May 19;15:150. doi: 10.1186/1471-2105-15-150 (PMC4229970; doi:10.1186/1471-2105-15-150)
Supplement: Additional file 2 — Java code of ml2db. Additional file ml2db_code.tar.gz contains the Java source code to run the multi-label machine learning experiments and save the results to database. The code’s Javadoc is included. [file 1471-2105-15-150-S2.zip › additional file 2/ml2db/ecmulan/doc/uk/ac/ed/inf/mulanxml/MulanLabel.html]

MulanLabel


JavaScript is disabled on your browser.


- Overview
- Package
- Class
- Use
- Tree
- Deprecated
- Index
- Help

- Prev Class
- Next Class

- Frames
- No Frames

- All Classes

- Summary:
- Nested |
- Field |
- Constr |
- Method

- Detail:
- Field |
- Constr |
- Method


uk.ac.ed.inf.mulanxml

## Class MulanLabel

- java.lang.Object
- - uk.ac.standrews.utils.main.webutils.simpledomparser.XmlNode
  - - uk.ac.ed.inf.mulanxml.MulanLabel

- ---

    

  ```
  public class MulanLabel
  extends uk.ac.standrews.utils.main.webutils.simpledomparser.XmlNode
  ```

  A node in the Mulan XML (a label for machine learning) \*

  Version:
  :   5 May 2010

  Author:
  :   Luna De Ferrari luna.deferrari-at-ed.ac.uk

- - ### Constructor Summary

    Constructors

    | Constructor and Description |
    | `MulanLabel(java.lang.String labelName)` |
  - ### Method Summary

    Methods

    | Modifier and Type | Method and Description |
    | `void` | `addChildElement(MulanLabel label)` Add a child label to a label |
    | `void` | `addChildElement(java.lang.String labelName)` Add a child label to a label |

    - ### Methods inherited from class uk.ac.standrews.utils.main.webutils.simpledomparser.XmlNode

      `addChildNode, getAttributeValue, getChildElements, getTagName, getText, getXmlTreeFromString, getXmlTreeFromUrl, hasChildren, nodeHeadToString, removeAttribute, removeChildNode, setAttribute, setTagName, setText, toString`
    - ### Methods inherited from class java.lang.Object

      `equals, getClass, hashCode, notify, notifyAll, wait, wait, wait`

- - ### Constructor Detail


    - #### MulanLabel

      ```
      public MulanLabel(java.lang.String labelName)
      ```
  - ### Method Detail


    - #### addChildElement

      ```
      public void addChildElement(MulanLabel label)
      ```

      Add a child label to a label

      Parameters:
      :   `label` - the child label (with its children etc.)


    - #### addChildElement

      ```
      public void addChildElement(java.lang.String labelName)
      ```

      Add a child label to a label

      Parameters:
      :   `labelName` - the name of the label to be added


- Overview
- Package
- Class
- Use
- Tree
- Deprecated
- Index
- Help

- Prev Class
- Next Class

- Frames
- No Frames

- All Classes

- Summary:
- Nested |
- Field |
- Constr |
- Method

- Detail:
- Field |
- Constr |
- Method
