# Supplementary material for: From sequence to enzyme mechanism using multi-label machine learning
Source: BMC Bioinformatics. 2014 May 19;15:150. doi: 10.1186/1471-2105-15-150 (PMC4229970; doi:10.1186/1471-2105-15-150)
Supplement: Additional file 2 — Java code of ml2db. Additional file ml2db_code.tar.gz contains the Java source code to run the multi-label machine learning experiments and save the results to database. The code’s Javadoc is included. [file 1471-2105-15-150-S2.zip › additional file 2/ml2db/ecmulan/doc/uk/ac/ed/inf/mulanxml/MulanXml.html]

MulanXml


JavaScript is disabled on your browser.


- Overview
- Package
- Class
- Use
- Tree
- Deprecated
- Index
- Help

- Prev Class
- Next Class

- Frames
- No Frames

- All Classes

- Summary:
- Nested |
- Field |
- Constr |
- Method

- Detail:
- Field |
- Constr |
- Method


uk.ac.ed.inf.mulanxml

## Class MulanXml

- java.lang.Object
- - uk.ac.ed.inf.mulanxml.MulanXml

- ---

    

  ```
  public class MulanXml
  extends java.lang.Object
  ```

  Generates an XML file for labels in the Mulan format
  http://mulan.sourceforge.net/ http://mlkd.csd.auth.gr/multilabel.html

  Example of Mulan XML format for labels:

  ```
   labels xmlns="http://mulan.sourceforge.net/labels"> 
   <label name="label1">  
   	<label name="label12"></label>  
   	<label name="label13"></label>  
   	<label name="label14"></label>  
   	<label name="label15"></label> 
   </label>
   /labels>
  ```

  Version:
  :   30 Apr 2010

  Author:
  :   Luna De Ferrari luna.deferrari-at-ed.ac.uk

- - ### Field Summary

    Fields

    | Modifier and Type | Field and Description |
    | `static java.lang.String` | `LABEL_NAME_ATTRIBUTE` the label tag name attribute |
    | `static java.lang.String` | `LABEL_XML_TAG` the label tag |
    | `MulanLabel` | `m_root` the xml tree root |
    | `static java.lang.String` | `MULAN_XML_ROOT_TAG` |
    | `static java.lang.String` | `MULAN_XML_ROOT_TAG_ATTRIBUTE_NAME` |
    | `static java.lang.String` | `MULAN_XML_ROOT_TAG_ATTRIBUTE_VALUE` |
  - ### Constructor Summary

    Constructors

    | Constructor and Description |
    | `MulanXml()` |
  - ### Method Summary

    Methods

    | Modifier and Type | Method and Description |
    | `MulanLabel` | `findNode(java.lang.String label)` Find a node by label |
    | `MulanLabel` | `getRoot()` |
    | `java.lang.String` | `toString()` public MulanLabel removeNode(String label) { // find node MulanLabel node = this.findNode(label); // get parent MulanLabel parent = node. |

    - ### Methods inherited from class java.lang.Object

      `equals, getClass, hashCode, notify, notifyAll, wait, wait, wait`

- - ### Field Detail


    - #### LABEL\_NAME\_ATTRIBUTE

      ```
      public static final java.lang.String LABEL_NAME_ATTRIBUTE
      ```

      the label tag name attribute

      See Also:
      :   Constant Field Values


    - #### LABEL\_XML\_TAG

      ```
      public static final java.lang.String LABEL_XML_TAG
      ```

      the label tag

      See Also:
      :   Constant Field Values


    - #### MULAN\_XML\_ROOT\_TAG

      ```
      public static final java.lang.String MULAN_XML_ROOT_TAG
      ```

      See Also:
      :   Constant Field Values


    - #### MULAN\_XML\_ROOT\_TAG\_ATTRIBUTE\_NAME

      ```
      public static final java.lang.String MULAN_XML_ROOT_TAG_ATTRIBUTE_NAME
      ```

      See Also:
      :   Constant Field Values


    - #### MULAN\_XML\_ROOT\_TAG\_ATTRIBUTE\_VALUE

      ```
      public static final java.lang.String MULAN_XML_ROOT_TAG_ATTRIBUTE_VALUE
      ```

      See Also:
      :   Constant Field Values


    - #### m\_root

      ```
      public MulanLabel m_root
      ```

      the xml tree root
  - ### Constructor Detail


    - #### MulanXml

      ```
      public MulanXml()
      ```
  - ### Method Detail


    - #### findNode

      ```
      public MulanLabel findNode(java.lang.String label)
      ```

      Find a node by label

      Parameters:
      :   `label` - the node label (the name attribute value), such as 'abc' in

          ```
           <label name="abc"></label>
          ```

      Returns:
      :   the node with that label, or null if not found


    - #### getRoot

      ```
      public MulanLabel getRoot()
      ```


    - #### toString

      ```
      public java.lang.String toString()
      ```

      public MulanLabel removeNode(String label) { // find node MulanLabel node
      = this.findNode(label); // get parent MulanLabel parent = node. // remove
      child node }

      **Overrides:**
      :   `toString` in class `java.lang.Object`


- Overview
- Package
- Class
- Use
- Tree
- Deprecated
- Index
- Help

- Prev Class
- Next Class

- Frames
- No Frames

- All Classes

- Summary:
- Nested |
- Field |
- Constr |
- Method

- Detail:
- Field |
- Constr |
- Method
