# Supplementary material for: From sequence to enzyme mechanism using multi-label machine learning
Source: BMC Bioinformatics. 2014 May 19;15:150. doi: 10.1186/1471-2105-15-150 (PMC4229970; doi:10.1186/1471-2105-15-150)
Supplement: Additional file 2 — Java code of ml2db. Additional file ml2db_code.tar.gz contains the Java source code to run the multi-label machine learning experiments and save the results to database. The code’s Javadoc is included. [file 1471-2105-15-150-S2.zip › additional file 2/ml2db/ecmulan/doc/uk/ac/ed/inf/mulanxml/package-summary.html]

uk.ac.ed.inf.mulanxml


JavaScript is disabled on your browser.


- Overview
- Package
- Class
- Use
- Tree
- Deprecated
- Index
- Help

- Prev Package
- Next Package

- Frames
- No Frames

- All Classes

# Package uk.ac.ed.inf.mulanxml

- Class Summary

  | Class | Description |
  |  |  |
  | --- | --- |
  | LocalDbReader | Reads the full list of Enzyme Commission numbers from database |
  | MulanLabel | A node in the Mulan XML (a label for machine learning) \* |
  | MulanXml | Generates an XML file for labels in the Mulan format http://mulan.sourceforge.net/ http://mlkd.csd.auth.gr/multilabel.html |
  | XmlCreator | Class |
  | XmlCreatorManager | Checks the nature of the data: if the labels are EC numbers, it generates an ECNumber xml creator, if not, it generates a plain xml creator. |
  | XmlCreatorManagerTest | Class |
  | XmlCreatorTest | Class |

- Overview
- Package
- Class
- Use
- Tree
- Deprecated
- Index
- Help

- Prev Package
- Next Package

- Frames
- No Frames

- All Classes
