# Supplementary material for: From sequence to enzyme mechanism using multi-label machine learning
Source: BMC Bioinformatics. 2014 May 19;15:150. doi: 10.1186/1471-2105-15-150 (PMC4229970; doi:10.1186/1471-2105-15-150)

This graph represents as red squares the proteins predicted as false positive when training on the mechanism dataset and testing on the negative set (using InterPro attributes, shown here as green rectangles).
The green oval represents the protein true mechanism, while the red oval is the mistaken prediction.
The yellow squares are neighbour proteins (proteins sharing some of the attributes) which caused the mis-prediction.
Authors: Luna De Ferrari and John Mitchell (2014)

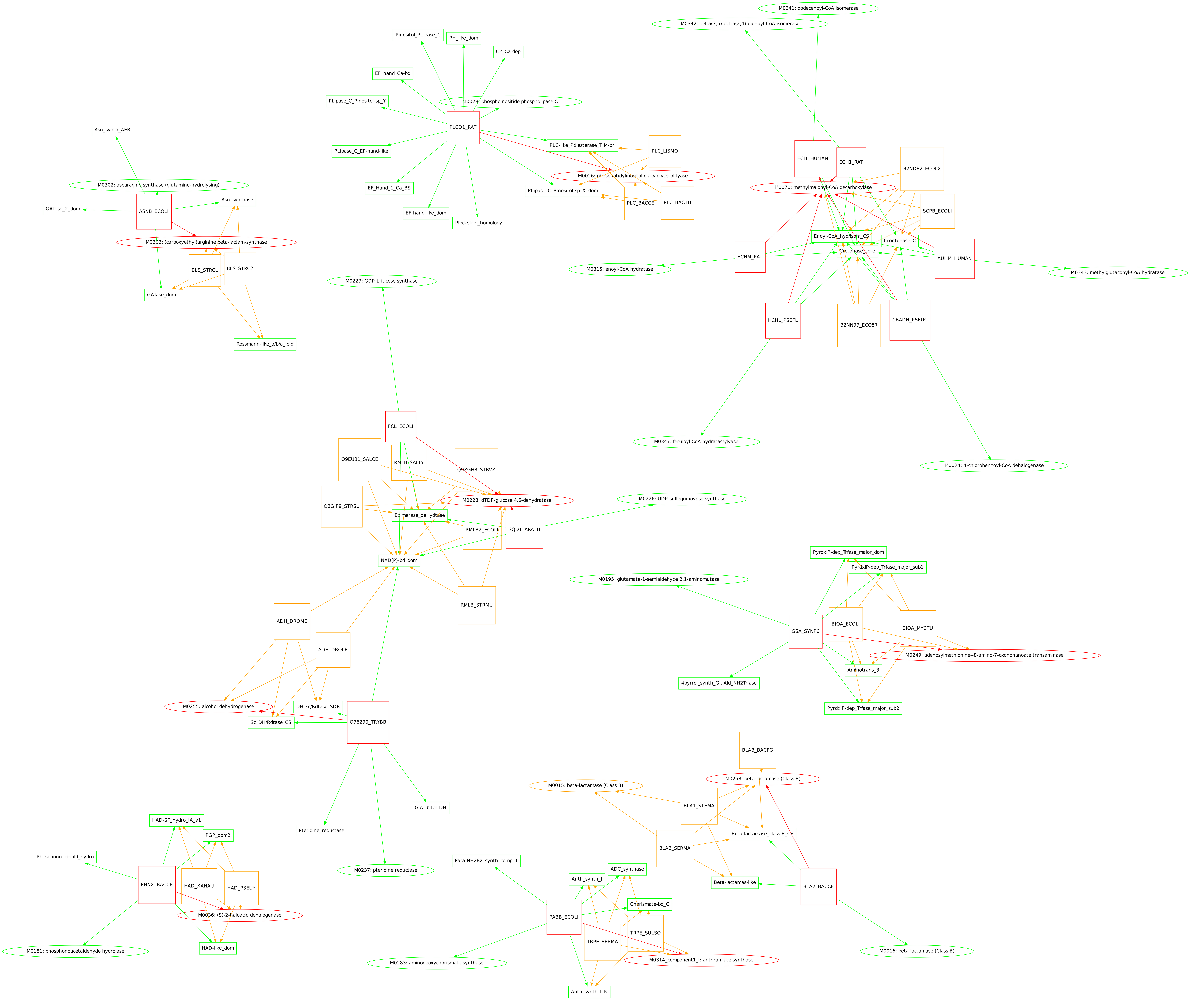

Supplement: Additional file 5 — Neighbours clusters of the false positive predictions when training on the mechanism set and testing on the negative set. Additional file graph_training_on_mechanism_testing_on_negative.pdf is a graph showing as red squares the proteins’ false positive labels when training on the mechanism set and testing on the negative set (using InterPro attributes, shown here as green rectangles). The green ovals represent the protein’s true mechanism, while the red ovals are the mistaken predictions. The yellow squares are neighbour proteins (proteins sharing some of the attributes) which caused the misprediction. The graph was generated with PyGraphviz, a Python interface to Graphviz. [file 1471-2105-15-150-S5.pdf]
